# Behavioral determinants of condom use and HIV/STI testing in Chile: a theory-driven mixed-methods study

**DOI:** 10.1038/s41598-026-43017-6

**Published:** 2026-03-06

**Authors:** Giuliano Duarte-Anselmi, Susana Sanduvete-Chaves, Daniel López-Arenas, Salvador Chacón-Moscoso

**Affiliations:** 1 Universitat de Barcelona, Barcelona, Spain; 2 Universidad de Santiago de Chile, Santiago, Chile; 3 Facultad de Psicología, Universidad de Sevilla, Sevilla, Spain; 4 Universidad Autónoma de Chile, Santiago, Chile

**Keywords:** HIV prevention, condom use, STI testing, COM-B, Theoretical domains framework (TDF), mixed methods, Diseases, Health care, Medical research, Psychology, Psychology

## Abstract

Condom use and HIV/STI testing are essential prevention behaviors, yet uptake remains low in Chile. Using data from the 2022–2023 National Health, Sexuality, and Gender Survey (ENSSEX), this study applied a theory-driven mixed-methods approach to identify behavioral determinants influencing these practices among Chilean adults. Considering sexually active respondents, only 23.4% reported consistent condom use and 16.8% reported HIV/STI testing in the past 12 months. Behavioral barriers to condom use were primarily related to perceived reduction in sexual pleasure, social norms within stable relationships, and limited behavioral regulation, while HIV/STI testing was more strongly facilitated by knowledge-related factors but constrained by low-risk perception and structural access barriers. Integrating quantitative classification with theory-informed interpretation using the COM-B model and the Theoretical Domains Framework enabled the identification of key motivational, emotional, and contextual drivers underlying these behaviors. These findings highlight the need for prevention strategies that move beyond information provision to address emotional, social, and structural determinants of behavior. This approach provides actionable guidance for designing behaviorally informed HIV/STI prevention policies and interventions, and demonstrates the value of applying behavioral science frameworks to national survey data in Chile and other middle-income settings.

**Supplementary Information:**

The online version contains supplementary material available at 10.1038/s41598-026-43017-6.

## Introduction

Sexually transmitted infections (STIs), including HIV, continue to pose a significant public health burden globally, with more than one million new infections reported daily^1^. In Chile, protective behaviors such as consistent condom use and routine testing remain suboptimal despite sustained national efforts to reduce STI and HIV incidence^2,3^. This behavioral gap highlights the critical need to understand not only what people do, but why they behave the way they do^1,2,4,5^.

Behavioral science provides robust frameworks for examining the cognitive, emotional, social, and environmental determinants that shape health-related decision-making^6,7^. The Capability, Opportunity, Motivation - Behavior (COM-B) model^8^ and the Theoretical Domains Framework (TDF)^9^ are among the most widely applied frameworks in implementation and behavioral research. These complementary models enable the systematic identification of modifiable factors that facilitate or impede behavior change.

According to the COM-B model, behavior results from the dynamic interaction of three components: Capability, Opportunity, and Motivation. Each component encompasses two sub-constructs, yielding six total: physical capability (physical skills and stamina) and psychological capability (knowledge and self-regulation skills); physical opportunity (access, time, and service availability) and social opportunity (social norms and interpersonal context); and reflective motivation (deliberate intentions and evaluations) and automatic motivation (emotional or habitual responses).The TDF builds upon these components by providing 14 theoretical domains derived from 33 behavior change theories, offering a more granular assessment of behavioral influences^9–11^.

**Fig. 1:**
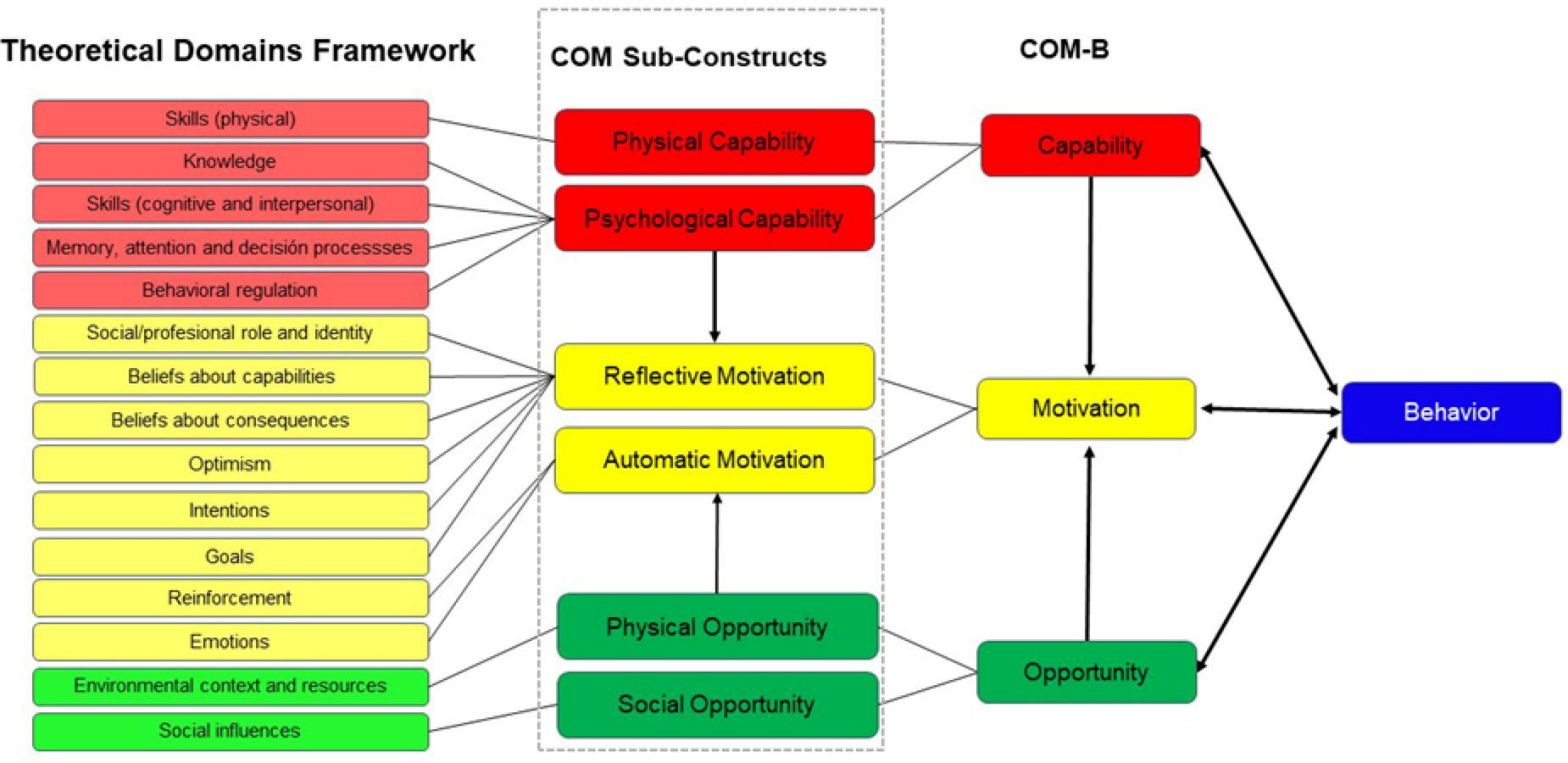
The Theoretical Domains Framework (TDF) mapped to the COM-B (Capability, Opportunity, Motivation - Behavior) model Adapted from Chater et al.^10^.

Together, the COM-B model and the Theoretical Domains Framework provide a structured approach for organizing behavioral correlates and linking them to intervention design^9,10^. Prior work has demonstrated how TDF domains can be systematically mapped onto COM-B components to support behavioral diagnosis and intervention development^12–14^. Figure 1 illustrates this integration.

Despite their widespread use in high-income countries, behavioral frameworks such as COM-B and TDF have rarely been applied in Latin American studies analyzing population-level health data. In Chile, national surveys capture a wide range of psychological, social, and structural variables related to sexual health, yet these data are often analyzed descriptively or in isolation. The 2022–2023 National Health, Sexuality, and Gender Survey (ENSSEX) provides a unique opportunity to apply these frameworks to identify behavioral determinants of condom use and HIV/STI testing within the sociocultural and structural conditions shaping sexual health behaviors in Chile^15^.

This study aims to apply a theory-informed, mixed-methods approach to explore behavioral determinants of condom use and HIV/STI testing among Chilean adults. Using COM-B, TDF, and AACTT frameworks, we systematically map relevant survey items and integrate quantitative analysis with qualitative interpretation to inform context-specific, behaviorally grounded intervention strategies.

### Research questions

This study employed a sequential mixed-methods design incorporating deductive theoretical mapping, quantitative analysis, and integrative interpretation, guided by the COM-B model, the Behavior Change Wheel (BCW), and the Theoretical Domains Framework (TDF).

### Primary research question

What are the barriers and facilitators influencing condom use and HIV/STI testing behaviors among Chilean adults, as identified in the National Health, Sexuality and Gender Survey (ENSSEX), using the COM-B, BCW, and TDF frameworks?

### Quantitative research questions

- What are the associations between consistent condom use and HIV/STI testing behaviors and psychological, social, emotional, and environmental factors identified in the ENSSEX survey?
- How do these behavioral outcomes vary according to capability, opportunity, and motivation, as defined by the COM-B model?

### Qualitative research question (deductive theoretical mapping and interpretation)

How do survey items mapped to TDF and COM-B framework domains reflect perceived barriers to and facilitators of condom use and HIV/STI testing behaviors among Chilean adults?

### Mixed-methods research question

How can the integration of theory-based quantitative findings and qualitative interpretation inform the design of behaviorally grounded strategies to promote condom use and HIV/STI testing in Chile?

### Research paradigm and theoretical framework

This study adopts a mixed-methods approach that integrates the generalizability and statistical rigor of quantitative analysis with the depth of qualitative inquiry to comprehensively examine behavioral determinants related to condom use and HIV/STI testing among Chilean adults^16–18^. From a pragmatic paradigm, the study explores “what works” by leveraging the complementary strengths of both methodological approaches. Quantitative data from the ENSSEX survey were deductively mapped using the TDF to identify behavior-related barriers and facilitators through a structured theoretical lens, which was further enriched through qualitative interpretation^9,12,19^.

Data transformation, which involves “quantitizing” qualitative data and “qualitizing” quantitative data, and other advanced integration strategies enhance the analytic scope and provide a more nuanced understanding of findings, particularly in population-based surveys^20^. These strategies align with Creswell and Plano-Clark’s integration framework, specifically “connecting” (where mapping results inform theoretical interpretation) and “embedding” (where theoretical frameworks like the TDF and COM-B model serve as organizing structures for quantitative analysis)^18^. This methodological approach strengthens the study’s capacity to generate context-sensitive evidence for the design of effective, culturally relevant, and evidence-based public health interventions.

## Methods

### Study design

A deductive mixed-methods design was employed, integrating quantitative and qualitative components to identify behavioral determinants of condom use and HIV/STI testing in Chile. The design was guided by the TDF, COM-B model, and AACTT framework to support systematic classification of behavioral influences. The study follows established best practices for mixed-methods research^18,21^, and draws on recent theory-informed syntheses in implementation science^14,22,23^.

This study adhered to the American Psychological Association’s Mixed Methods Article Reporting Standards (MMARS) to ensure transparent reporting of the rationale, sequencing, and integration of methods^21^, as detailed in Supplementary Appendix 1. Integration strategies were based on established typologies including connecting and embedding^24^, linking quantitative item classification with theory-driven qualitative interpretation and reinforcing alignment with the COM-B, TDF, and BCW frameworks.

Sex and gender considerations were incorporated throughout, following the Sex and Gender Equity in Research (SAGER) guidelines^25^. Further details are included in Supplementary Appendix 2.

The mixed-methods approach was motivated by the need to move beyond descriptive epidemiological patterns and toward a theory-informed behavioral diagnosis of HIV/STI prevention behaviors. While national surveys such as ENSSEX provide rich population-level data on behaviors and related factors, descriptive statistics alone are insufficient to explain why individuals engage in or avoid protective practices. By integrating quantitative classification of survey items with theory-driven interpretive synthesis, this study identifies the motivational, emotional, social, and contextual mechanisms underlying condom use and HIV/STI testing. This approach enables population data to be translated into intervention-relevant insights, supporting the design of behaviorally grounded public health strategies.

### Data source

Data were obtained from the Encuesta Nacional de Salud, Sexualidad y Género [National Survey on Health, Sexuality and Gender] (ENSSEX) 2022–2023^15^, a nationally representative, cross-sectional survey coordinated by the Chilean Ministry of Health and implemented by DESUC, Pontificia Universidad Católica de Chile. The survey was conducted using computer-assisted personal interviews (CAPI) between August and December 2022 and included over 20,000 participants ages 18 and over.

The survey employed a multistage, stratified sampling, yielding a weighted sample representative of approximately 13.4 million adults residing in urban areas of Chile. Oversampling of the 18–34 and 60 + age groups ensured analytic power for age-disaggregated insights.

### Analysis

**Fig. 2:**
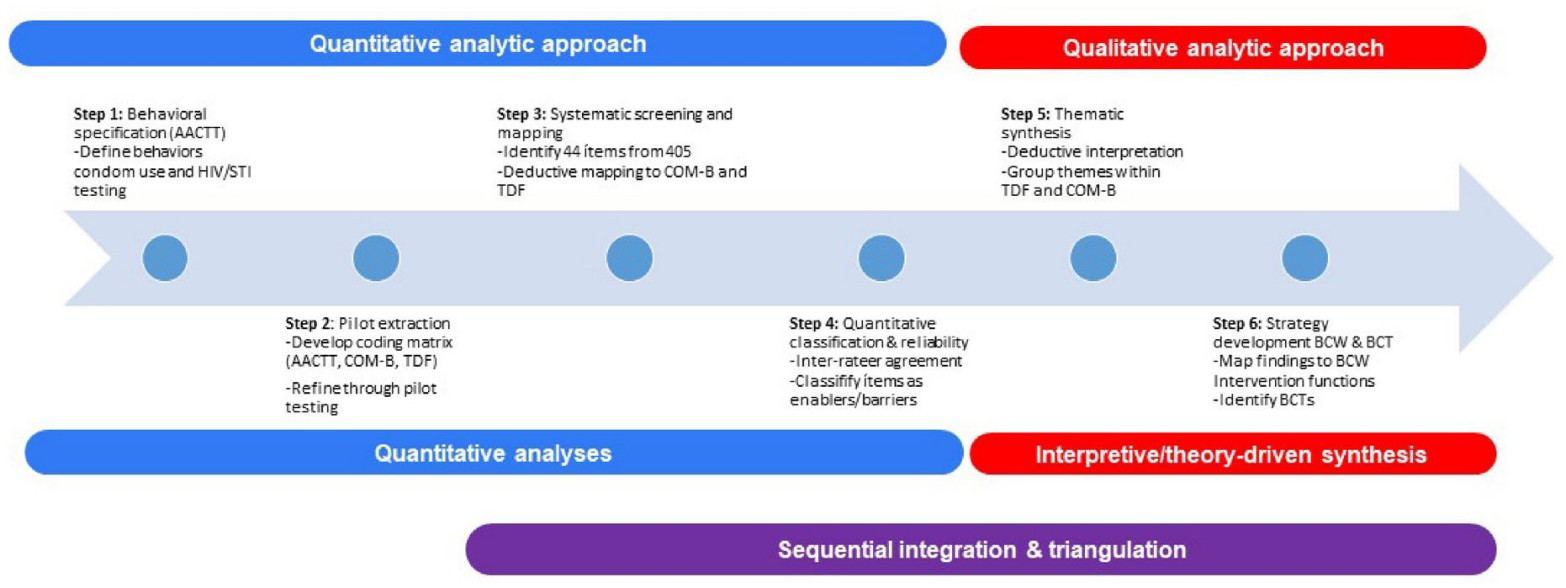
Six-step analytic strategy using a sequential mixed-methods framework to identify behavioral determinants of condom use and HIV/STI testing.

This study employed an explanatory sequential design guided by the COM-B model, the Theoretical Domains Framework (TDF), and the Action, Actor, Context, Target, Time (AACTT) framework. These theoretical models enabled the systematic identification, classification, and interpretation of behavioral determinants related to condom use and HIV/STI testing among Chilean adults. The analytic procedure comprised six structured steps integrated across four methodological components. Figure 2 depicts the six analytic steps conducted in the study, which integrated quantitative and qualitative methods using a theory-driven sequential design.

Figure 2 provides a visual overview of the sequential mixed-methods analytic strategy applied in this study. Steps 1–4 correspond to the quantitative analytic approach, including behavioral specification, item screening, and theory-driven classification, while Steps 5–6 represent the qualitative analytic approach focused on thematic synthesis and strategy development. It also illustrates how both strands were integrated through sequential integration and triangulation to support a comprehensive behavioral diagnosis. The final synthesis triangulated findings across both strands. Behavioral mapping was guided by the AACTT framework, COM-B model, TDF, the Behavior Change Wheel (BCW), and the Behavior Change Technique Taxonomy (BCTTv1). For clarity and readability, detailed item-level wording, descriptive statistics, and classification thresholds are reported in the Supplementary Materials, while the main manuscript presents condensed and illustrative summaries.

Detailed item wording, coding matrices, classification thresholds, interrater reliability procedures, extended tables, and supplementary figures are provided in the Supplementary Materials (Appendices 1–9 and Tables S1–S3). These materials support transparency and replicability but are not required for interpretation of the main findings.

### Quantitative

To precisely define the target behaviors —condom use and HIV/STI testing— we applied the Action, Actor, Context, Target, Time (AACTT) framework, which facilitates the explicit specification of health behaviors in survey-based research. The behaviors were defined as follows:

#### Step 1: behavioral specification using AACTT

- **Action:** Condom use and HIV/STI testing.
- **Actor: **Sexually active individuals participating in the ENSSEX survey.
- **Context: **Sexual activity or healthcare interactions.
- **Target: **The respondent or their sexual partner(s).
- **Time: **During or prior to sexual activity, or during health-related consultations.

This behavioral definition guided the selection of relevant items for theoretical mapping.

#### Step 2: pilot extraction and development of coding matrix

A structured coding matrix was developed to organize each selected item by variable name, full item wording, AACTT specification, COM-B component(s), TDF domain(s), and justification for inclusion. A subset of items was independently piloted by two researchers, GDA and DLA, to refine the coding logic. The final matrix incorporated feedback from senior authors SSC and SCM and is included in Supplementary Appendix 3.

Mapping procedures were based on established methodological applications in behavioral research and implementation science^14,22,23^. Each selected item was assigned to one or more TDF domains and COM-B components using a coding matrix that included survey item wording, variable name, AACTT specification, justification for inclusion, TDF classification, and COM-B mapping. This matrix is presented in Supplementary Appendix 4.

#### Step 3: Systematic screening and theoretical mapping

All 305 structured items in the ENSSEX 2022–2023 questionnaire (405 individual variables, including sub-items) were independently reviewed by two researchers (GDA, DLA). Using a deductive approach, 44 items were identified as relevant to the psychological, social, or contextual determinants of the target behaviors. Each item was then mapped to one or more domains of the TDF and corresponding components of the COM-B model, based on operational definitions from established sources^8,9^.

Although Steps 1–4 are grouped as the quantitative analytic approach in Fig. 2, this stage incorporated a qualitative, interpretive component. Specifically, survey items were deductively mapped to COM-B components and TDF domains using a structured coding matrix. This represents a theory-driven coding procedure applied to structured quantitative data, consistent with explanatory sequential mixed-methods designs. GDA and DLA independently performed the classification, resulting in excellent interrater reliability (Cohen’s Kappa = 0.96)^26,27^. Any discrepancies were resolved through discussions in which SSC mediated to reach consensus (see Supplementary Appendix 5).

#### Step 4: Quantitative classification and inter-coder reliability

Each item was grouped into behavioral themes (e.g., Beliefs about Consequences, Social Norms, Emotional Drivers). Descriptive statistics (means, standard deviations, frequencies, and percentages) were calculated for all items to characterize response distributions prior to classification, consistent with survey-based approaches applied in behavioral health research^23^. Likert-type ordinal variables were summarized using means and standard deviations, following established practice in behavioral research that treats such data as approximately interval-scaled for descriptive and classificatory purposes. Although this practice has been debated^28^, it has been adopted and defended in behavioral and public health research, particularly when justified by large sample sizes and theory-driven analytic objectives^29–31^. Following this rationale, means and standard deviations were used to describe item distributions, consistent with recommendations in behavioral survey research^32,33^. Although medians and interquartile ranges are sometimes reported for ordinal data, the use of means and standard deviations was considered appropriate here given the analytic objective of applying theory-informed classification thresholds to behavioral determinants, rather than conducting distributional comparisons across items. Reversed scoring was applied to negatively worded items so that lower scores consistently reflected higher barriers, ensuring directional consistency across measures.

GDA and DLA independently analyzed 100% of items. Likert-scale items were recoded into three categories (major barrier, moderate barrier, or enabler) using predefined thresholds informed by prior research and theory-driven classification criteria.

For 7-point Likert items, responses were categorized based on frequency distribution: ≥70% of responses in the upper range (scores 5–7) were classified as enablers, 40–69% as moderate barriers and < 40% as major barriers. For 5-point Likert items, classification was based on mean values, where a mean ≥ 4.00 classified as enablers, mean between 3.00 and 3.99 as moderate barriers, and mean ≤ 2.99 as major barriers. Thresholds for classifying items as enablers, moderate barriers, or major barriers were defined a priori based on their interpretive usefulness and alignment with prior theory-based COM-B/TDF applications. Similar heuristic cut-offs have been used in previous survey-based behavioral diagnosis studies to support prioritization and intervention planning, rather than to imply strict psychometric boundaries (e.g., Shapoval et al.2025)^23^.

For dichotomous and categorical variables, the same percentage-based thresholds were applied (e.g., ≥ 70% classified as enablers, < 40% as major barriers), guided by theoretical alignment with COM-B and TDF domains and response distribution patterns. These thresholds were theory-informed and consistent with prior behavioral research^14,23^. Results are color-coded in Supplementary Table 1 (green = enabler, gray = moderate barrier, red = major barrier) (see Supplementary Appendix 6). All quantitative analyses and descriptive statistics were conducted using Stata version 17 (StataCorp LLC, College Station, TX).

### Qualitative

Narrative responses were evaluated using deductive thematic analysis. Responses were coded to the corresponding TDF domain and grouped into behavioral themes.

#### Step 5: Thematic synthesis of behavioral determinants

**Fig. 3:**
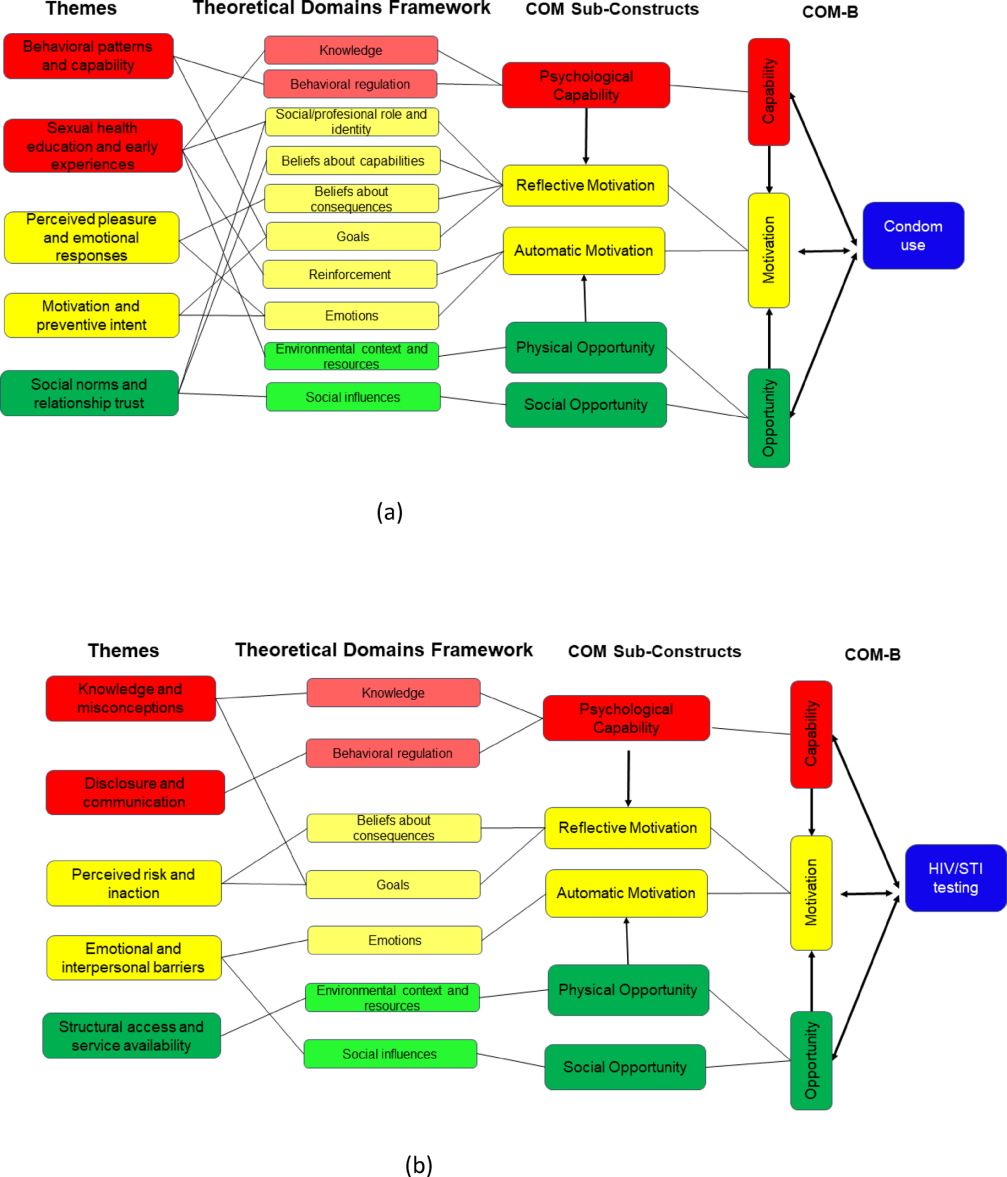
(**a**) Summary of the themes related to behavioral determinants of condom use within TDF and COM-B domains. (**b**) Summary of the themes related to behavioral determinants of HIV/STI testing within TDF and COM-B domains.

To complement the quantitative findings, we conducted a deductive narrative synthesis of the 44 mapped items. Each item was interpreted within its assigned TDF domain and COM-B component and then grouped into themes reflecting key behavioral constructs (e.g., risk perception, emotional discomfort, social influence). This interpretive step applied qualitative reasoning to structured data and followed established approaches to theory-driven synthesis in behavior change research (see Supplementary Appendices 7 and 8). Behavior-specific COM-B diagrams were created to illustrate the main drivers and barriers for condom use and HIV/STI testing behaviors (Fig. 3a and b) (Tables 1, 2, 3 and 4).

### Triangulation of quantitative and qualitative results

Quantitative and qualitative findings were integrated sequentially to enhance interpretative depth. Themes identified in Step 5 were cross-referenced with the item classifications from Step 4, allowing triangulation across methods and assessment of convergence or divergence between behavioral constructs. For example, items identified quantitatively as major barriers (e.g., beliefs about reduced pleasure, fear of partner judgment) were further elaborated through narrative synthesis as indicating low reflective motivation and restricted social opportunity, respectively. This approach followed best practices for mixed-methods integration^18^.

#### Step 6: Strategy development and linking behavioral determinants to intervention functions and BCTs

In the final step, the behavioral diagnosis was translated into theoretically grounded, actionable intervention strategies. This process followed the Behavior Change Wheel (BCW) framework^10^, which links behavioral determinants to evidence-based intervention functions.

Each barrier or enabler identified in the previous steps was mapped to:

- the corresponding COM-B component (e.g., reflective motivation, social opportunity).
- one or more TDF domains (e.g., Beliefs about Consequences, Social Influences).
- relevant BCW intervention functions (e.g., education, persuasion, enablement).
- specific Behavior Change Techniques (BCTs) from the BCT Taxonomy v1^34,35^.
- concrete examples of strategies to encourage condom use or HIV/STI testing in Chile.

This mapping produced a set of theory-informed recommendations, presented in Table 5.

### Data note

Only closed-ended questions from the ENSSEX 2022–2023 dataset were analyzed. Open-ended questions were excluded, as they were incompatible with the deductive coding framework used.

## Results

### Overview of the dataset

A total of 405 items from the ENSSEX 2022–2023 national survey—including primary questions and sub-items—were reviewed. Of these, 44 items were identified as relevant to condom use and HIV/STI testing and were deductively mapped to domains of the TDF, guided by the AACTT framework.

The represented TDF domains among the selected items were Environmental Context and Resources (11 items), Knowledge (9), Behavioral Regulation (8), Beliefs about Consequences (5), Social Influences (4), Beliefs about Capabilities (3), Goals (3), and Reinforcement (1). Interrater agreement for TDF coding was excellent (Cohen’s κ = 0.96). Full details are provided in Supplementary Materials.

### Participant characteristics

The ENSSEX survey included 20,392 adults ages 18 and older (M = 44.9, SD = 18.1), selected via a four-stage, stratified sampling design. After applying sample weights, the data represent approximately 13.4 million urban-dwelling adults across Chile. The sex assigned at birth distribution was skewed toward females (66.47% female; 33.53% male), with the majority of participants ages 35 to 59.

Among respondents, 62.7% reported being sexually active in the previous 12 months. Within this group, 23.4% reported consistent condom use and 16.8% reported having undergone HIV/STI testing.

Several contextual indicators provided further insight into the behavioral environment: 71.2% reported that sexual topics were not discussed within their families during childhood; 33.4% had discussed STI or pregnancy prevention with their partner before their first sexual encounter; and more than 50% rated school sex education as poor or very poor. Additional descriptive data are available in Supplementary Materials.

While these contextual variables were not formally mapped as behavioral determinants, they informed the triangulation and theoretical interpretation of findings. The 44 selected items were mapped to the COM-B model and TDF domains and subsequently classified using theory-driven thresholds.

### Quantitative classification of behavioral determinants

The 44 behaviorally relevant items were categorized into four groups based on predefined response criteria aligned with COM-B and TDF constructs:

- 11 items (25%) were classified as enablers.
- 13 items (30%) were classified as moderate barriers.
- 5 items (11%) were classified as major barriers.
- 15 items (34%) were retained for descriptive use only.

Table 1 presents a condensed, high-level summary of key behavioral determinants of condom use and HIV/STI testing, classified as enablers, moderate barriers, or major barriers within COM-B and TDF domains. To enhance clarity and readability, the table includes only summary labels, agreed TDF domains, COM-B components, and final classifications. Full item wording, descriptive statistics, classification thresholds, and detailed COM-B/TDF mappings for all 44 ENSSEX items are provided in Supplementary Tables S1–S3 and Supplementary Appendices 5–8.

**Table 1:** Summary of key behavioral determinants of condom use and HIV/STI testing classified as enablers or barriers within COM-B and TDF domains.

Variable	Questionnaire item	Summary label	Agreed TDF domain	COM-B	Final classification
p56	In that first sexual intercourse, did you use any contraceptive method?	Contraceptive use at first sex	Behavioral regulation	Capability	Major barrier
p73	In your sexual relationships over the past year, how often did you use condoms?	Condom use frequency (last year)	Behavioral regulation	Capability	Major barrier
p89	In the first sexual encounter you had again with that person after the separation, did you use a condom?	Condom use after reconciliation	Behavioral regulation	Capability	Major barrier
p119	In that last sexual encounter, did you use any contraceptive method?	Contraceptive use in last sex	Behavioral regulation	Capability	Major barrier
p208	For any reason, have you had an HIV test in the last 12 months?	HIV test in past 12 months	Behavioral regulation	Capability	Major barrier
p34	When you were a child, did your family talk about sexual topics?	Family discussed sexual topics in childhood	Social influences	Opportunity	Major barrier
t_p36_1	In your school, when you were a student, was sex education taught in primary school?	Sex education in primary school	Environmental context and resources	Opportunity	Major barrier
p38	How would you evaluate the sexuality education you received in school?	Perceived quality of sex education	Beliefs about capacity	Motivation	Major barrier
p55	Before your first sexual intercourse, did you and your partner talk about how to avoid STIs…?	Communication before first sex	Social influences	Motivation	Major barrier
i_4_p33	According to what you believe, does using condoms enhance sexual play?	condom use enhances sexual play	Beliefs about consequences	Motivation	Major barrier
i_2_p33	According to what you believe, how much do you agree that using condoms reduces men’s sexual pleasure?	Condoms reduce sexual pleasure in men	Beliefs about consequences	Motivation	Major barrier
i_3_p33	According to what you believe, is it necessary to use condoms even when in a stable relationship?	condom use necessary even in stable relationships	Knowledge	Capability	Major barrier
i_1_p212	Can the risk of HIV transmission be reduced by having sex with one faithful partner who does not have HIV/AIDS?	Knowledge of condom efficacy for HIV	Knowledge	Capability	Enabler
i_2_p212	Can the risk of HIV transmission be reduced by using condoms every time you have sex?	Knowledge of condom efficacy for HIV	Knowledge	Capability	Enabler
i_3_p212	Can a healthy-looking person have HIV?	Knowledge that HIV may be asymptomatic	Knowledge	Capability	Enabler
i_5_p33	According to what you believe, are condoms too expensive to use regularly?	condoms are too expensive	Environmental context and resources	Opportunity	Enabler
p35	And how often did you participate when sexual topics were discussed?	Participation in sexual topic conversations	Social influences	Opportunity	Enabler
i_1_p33	According to what you believe, how much do you agree that using condoms reduces women’s sexual pleasure?	Condoms reduce sexual pleasure in women	Beliefs about consequences	Motivation	Enabler

Table 1 presents a condensed, theory-oriented summary of key behavioral determinants. Full item-level details, including item wording, scales, descriptive statistics, and classification thresholds, are provided in Supplementary Table S1.

#### Behavior-specific patterns

When disaggregated by target behavior, distinct patterns emerged across the 44 classified items (see Table 2).

**Table 2:** Classification of behavioral determinants by target behavior.

Target Behavior	Enablers	Moderate barriers	Major Barriers	Descriptive use only	Total items
Condom use	5	3	6	8	22
HIV/STI testing	7	4	1	10	22
Total	12 (27%)	7 (16%)	7 (16%)	18 (41%)	44 (100%)

Condom use-related items (*n* = 22) were more frequently classified as barriers (9 of 22; 41%), particularly those mapped to the domains of Beliefs about Consequences, Social Influences, and Behavioral Regulation. These items reflected challenges such as perceived reduction in sexual pleasure, lack of early sexual health education, and inconsistent condom use. Five items (23%) were classified as enablers, while eight (36%) were retained for descriptive use only due to categorical response formats. To illustrate how survey items were theoretically mapped and classified, Table 3 presents a selected subset of condom use–related items, showing their alignment with TDF domains, COM-B components, and final classification. Full item-level mappings and classifications for all condom use items are provided in Supplementary Table S1 and S2.

HIV/STI testing-related items (*n* = 22). Seven of 22 (32%) were classified as enablers, especially items associated with the domains of Knowledge, Goals, and Behavioral Regulation. One item (5%) was classified as a major barrier, while four (18%) were categorized as moderate barriers, and ten items (45%) were retained for descriptive use only due to nominal scaling. Table 4 provides an illustrative subset of HIV/STI testing–related items, demonstrating the application of COM-B and TDF to item classification. Complete item-level results are reported in Supplementary Table S3.

**Table 3:** Illustrative classification of selected condom use–related survey items according to COM-B and TDF domains.

Item	Variable	Summary label	Agreed TDF domain	COM Sub-Constructs	COM-B	Final classification
1	i_1_p33	Condoms reduce sexual pleasure in women	Beliefs about Consequences	Reflective Motivation	Motivation	Enabler
2	i_2_p33	Condoms reduce sexual pleasure in men	Beliefs about Consequences	Reflective Motivation	Motivation	Moderate barrier
3	i_3_p33	Condom use necessary even in stable relationships	Knowledge	Psychological Capability	Capability	Moderate barrier
4	i_4_p33	Condom use enhances sexual enjoyment	Beliefs about Consequences	Reflective Motivation	Motivation	Moderate barrier
5	i_5_p33	Condoms are too expensive	Environmental Context and Resources	Physical Opportunity	Opportunity	Enabler
6	p34	Family discussed sexuality in childhood	Social Influences	Social Opportunity	Opportunity	Moderate barrier
7	p35	Participation in family conversations about sexuality	Social Influences	Social Opportunity	Opportunity	Enabler

**Table 4:** Illustrative classification of selected HIV/STI testing–related survey items according to COM-B and TDF domains.

Item	Variable	Summary label	Agreed TDF domain	COM sub-constructs	COM-B	Final classification
1	p37	Perceived adequacy of sex education	Beliefs about Capacity	Reflective Motivation	Motivation	Major barrier
2	p38	Perceived quality of sex education	Beliefs about Capacity	Reflective Motivation	Motivation	Major barrier
3	i_2_p39	Knowledge of STI prevention	Knowledge	Psychological Capability	Capability	Major barrier
9	p119	Contraceptive use in last sex	Behavioral Regulation	Psychological Capability	Capability	Major barrier
10	p151	Ever sought sexual health consultation (women only)	Environmental Context and Resources	Physical Opportunity	Opportunity	Major barrier
13	p208	HIV test in past 12 months	Behavioral Regulation	Psychological Capability	Capability	Major barrier
16	i_1_p212	*Knowledge of condom efficacy for HIV*	Knowledge	Psychological Capability	Capability	Enabler
18	i_3_p212	Knowledge that HIV may be asymptomatic	Knowledge	Psychological Capability	Capability	Enabler
19	i_4_p212	Rejects HIV transmission via mosquitos	Knowledge	Psychological Capability	Capability	Enabler
22	p213	Awareness of PrEP for HIV prevention	Knowledge	Psychological Capability	Capability	Major Barrier

### Qualitative results

#### Thematic synthesis of behavioral determinants

A theory-informed narrative synthesis was conducted to complement the quantitative classification. The 44 structured items were grouped according to behavioral relevance and mapped to domains of the TDF and COM-B model. Themes and subthemes were identified deductively based on conceptual alignment with the frameworks. To maintain behavioral specificity, results are presented separately for condom use and HIV/STI testing (Fig. 3a and b). Figure 3a provides an integrative summary of the main behavioral drivers and barriers related to condom use, synthesizing quantitative item classification with theory-informed qualitative interpretation across COM-B and TDF domains. Figure 3b summarizes the key behavioral determinants of HIV/STI testing, illustrating how quantitative classifications and qualitative thematic interpretations converge across COM-B and TDF domains.

### Condom use

#### Theme 1: Perceived pleasure and emotional responses

Participants expressed ambivalence about the sensory and emotional impact of condom use. The belief that condoms reduce sexual pleasure for men was moderately endorsed, with 41.6% of respondents agreeing or strongly agreeing with the statement (M = 3.02, SD = 1.07), leading to its classification as a moderate barrier. In contrast, only 31.2% of participants endorsed the belief that condoms reduce women’s pleasure (M = 2.79, SD = 1.04), which was classified as an enabler. Furthermore, only 30.1% of respondents agreed or strongly agreed that condom use enhances sexual enjoyment, while 43.8% disagreed or strongly disagreed. This item was classified as a major barrier (M = 2.83, SD = 0.98). These findings align with the TDF domains Beliefs about Consequences and Emotion, within the COM-B motivation component, that is, both conscious decision-making and more automatic emotional or habitual responses. The data suggest that perceived reduction in pleasure, especially among men, along with limited positive framing of condom use, may hinder consistent use.

#### Theme 2: Social norms and relationship trust

Social norms that discourage condom use within stable relationships emerged as significant behavioral barriers. When asked whether condom use is necessary even in a stable relationship, only 45.5% of participants agreed or strongly agreed, while 25.7% disagreed or strongly disagreed, and nearly one-third (28.8%) remained ambivalent (M = 3.09, SD = 1.07). This item was classified as a moderate barrier. These findings suggest that trust within relationships may serve as a perceived substitute for protection, reflecting social norms in which emotional bonds are presumed to guarantee safety, thus discouraging condom use. This aligns with the Social Influences and Beliefs about Capabilities domains of the TDF, and maps to social opportunity in the COM-B model. Interpersonal scripts and implicit assumptions about fidelity may weaken the perceived need for condom use and protection in long-term relationships.

#### Theme 3: Sexual health education and early experiences

Gaps in early sexual health education and limited communication within families emerged as significant barriers to consistent condom use. Only 7.2% of participants reported that their families regularly discussed sexual topics during their childhood (p34), making this item as a major barrier. However, among those who reported some degree of discussion in their family, 89.9% indicated they at least occasionally engaged in the discussions (p35), suggesting variability in the depth and continuity. Major shortcomings were also detected in sex education: only 30.3% reported having sex education during elementary school (t_p36_1) and 52.1% during high school (t_p36_2). When asked to evaluate the adequacy of that education, just 7.1% rated it as “more than needed” (p37), and only 23.0% considered it “good” or “very good” (p38). When mapped to the Knowledge, Environmental Context and Resources, and Beliefs about Capabilities domains, these findings reflect substantial limitations in both psychological capability and structural opportunity for early sexual health literacy.

#### Theme 4: Behavioral patterns and capability

Condom use behaviors across different sexual contexts revealed low levels of self-regulation and intentional control. Only 33.4% of respondents reported using a contraceptive method during their first sexual experience (p56), and just 17.5% reported always using condoms in the past year (p73). Similarly, 35.9% indicated they used condoms after reconciling with a previous partner (p89), and 36.9% reported using a contraceptive method in their most recent sexual encounter (p119). All these items were classified as major barriers and reflect persistent challenges in behavioral regulation. These findings map to the Behavioral Regulation and Goals domains of the TDF and correspond to psychological capability in the COM-B model, specifically gaps in self-regulation skills such as planning and follow-through.

#### Theme 5: Motivation and intention to prevent STIs

Although a large majority (79.9%) of respondents who reported condom use stated that their main motivation was the prevention of STIs or HIV (p121), actual use remained circumstantial. As reported previously, only 17.5% used condoms consistently over the past year (p73), and fewer than 37% did so in their most recent or reconciliatory sexual encounters (p119, p89). This disconnect between strong intentions to prevent STIs and inconsistent use points to gaps in behavioral enactment. These findings reflect tensions between the Goals and Emotion domains of the TDF, and highlight how reflective motivation fails to drive behavior in the COM-B model. Fear of rejection, emotional discomfort, or perceived loss of spontaneity may override intentions to use a condom during sexual encounters.

### HIV/STI testing

#### Theme 1: Perceived risk and inaction

A perception of low risk and low testing uptake emerged as major barriers to HIV/STI screening. Only 22.6% of respondents reported having undergone an HIV test in the past 12 months (p208), indicating limited engagement in preventive health actions. Even when respondents reported being sexually active and having previous encounters without condoms, they tested infrequently. This finding reflects a potential underestimation of personal risk and aligns with the Beliefs about Consequences and Goals domains of the TDF. Within the COM-B framework, this reflects low perceived urgency and weak intention to test, indicating limited motivation to seek preventive care.

#### Theme 2: Structural access and service availability

Persistent structural barriers in the Chilean health system were reflected in limited engagement with sexual health services and prevention tools. Among women, 68.7% had consulted a healthcare provider for topics related to sexuality or STI prevention, compared to just 24.3% of men—highlighting a substantial gender gap in healthcare access (p151, p152). In addition, awareness of pre-exposure prophylaxis (PrEP) was extremely low: only 10.5% of respondents reported knowing about this preventive measure (p213). These determinants, mapped to the Environmental Context and Resources and Knowledge domains of the TDF, limit physical opportunity in the COM-B model. While the survey did not directly measure time constraints or logistical difficulties, the findings point to structural inequities that may hinder timely and proactive STI testing, particularly among men and those unaware of biomedical prevention options.

#### Theme 3: Knowledge and misconceptions

General knowledge about HIV transmission and prevention was high across multiple domains. Most respondents correctly recognized that the risk of HIV transmission can be reduced through monogamy with a partner who does not have HIV (76.4%) and consistent condom use (85.1%); the majority were also aware that healthy-looking individuals can still carry the virus (85.7%) (i_1_p212 to i_3_p212). Additionally, most participants rejected common misconceptions: 74.6% correctly identified mosquito transmission as false, 82.3% knew that HIV is not transmitted through sharing food, and 82.0% were aware of the risk of mother-to-child transmission during pregnancy or childbirth (i_4_p212 to i_6_p212). All of these items were classified as enablers. These findings span the Knowledge and Goals domains of the TDF and map to strong knowledge and basic prevention understanding (knowledge and skills) within COM-B capability.

#### Theme 4: Emotional and interpersonal barriers

Emotional and interpersonal dynamics significantly influenced HIV/STI testing behavior. Among those who had not been tested in the last year, 68.3% cited a lack of perceived risk (p211) and 25.4% either did not consider testing necessary or simply had not thought about it. These findings suggest motivational inaction, where emotional reassurance and cognitive avoidance replace proactive decision-making. Although relatively few reported an explicit fear of a positive result (0.24%) or of the stigma associated with testing (0.37%), these barriers remain present. Notably, among those diagnosed with an STI, 61.3% only became aware of the infection after experiencing symptoms (p206), further reinforcing that testing is often reactive rather than preventive. These patterns map to the Emotion and Social Influences domains of the TDF, and reflect both automatic motivation and shaped by stigma-related social norms and interpersonal concerns (COM-B social opportunity).

#### Theme 5: Disclosure and communication

Participants with STI diagnoses frequently indicated they had informed their partners: 70.3% answered affirmatively (p207), classifying this behavior as an enabler. This pattern, categorized under the Behavioral Regulation domain of the TDF, suggests a promising entry point for interventions aimed at strengthening interpersonal communication and normalizing partner notification practices. It reflects a level of psychological capability within the COM-B model, and offers an opportunity to reinforce positive disclosure behaviors as part of routine STI prevention strategies.

### Triangulation of quantitative and qualitative results

The quantitative strand constituted the primary empirical backbone of the analysis, systematically identifying and classifying behavioral determinants across 44 survey items using theory-driven thresholds. The qualitative narrative synthesis did not function as an independent analytic strand, but rather as an interpretive layer applied at the triangulation stage (Step 5) to contextualize, explain, and theoretically situate quantitatively identified patterns.

To enhance the depth of findings, we triangulated the quantitative classifications (Step 4) and the narrative synthesis (Step 5) using the theory as a guide. Each behavioral determinant was examined across both approaches to assess convergence, complementarity, or divergence. This integrative process allowed for a nuanced understanding of whether statistical patterns align with theoretically derived themes and if so, how.

For instance, the quantitative classification identified beliefs about reduced sexual pleasure (items i_1_p33 and i_2_p33) as moderate or major barriers. These items were further interpreted qualitatively within the theme perceived pleasure and emotional responses, aligned with the Beliefs about Consequences and Emotions domains, and mapped to reflective and automatic motivation in the COM-B model. Similarly, low frequencies of condom use after partner reconciliation (item p89) were quantitatively classified as a major barrier and qualitatively understood as a pattern of diminished self-regulation and goal-setting capacity.

This cross-validation also revealed areas of partial divergence. While HIV-related knowledge items (e.g., i_1_p212–i_6_p212) were quantitatively categorized as enablers, the qualitative synthesis identified gaps in awareness of biomedical prevention tools—most notably PrEP (item p213)—highlighting persistent knowledge gaps despite general cognitive capability.

The integration resulted in a theory-driven translation of key barriers and enablers into actionable strategies (Step 6), using the Behavior Change Wheel (BCW) framework. Each determinant was mapped to its corresponding COM-B component and TDF domain, linked to suitable intervention functions (e.g., education, enablement), and paired with recommended behavior change techniques (BCTs) such as “Information about Health Consequences” or “Credible Source.” Table 5 summarizes this mapping for both condom use and HIV/STI testing behaviors.

This final stage of the analysis yielded targeted, theory-informed recommendations for behavior change interventions, tailored to the Chilean context and supported by behavioral science evidence.

Taken together, the triangulation of quantitative classifications and qualitative synthesis generated a more nuanced understanding of sexual health behaviors than either approach alone. Quantitative analyses identified the relative weight and distribution of behavioral determinants, highlighting where barriers and enablers clustered across domains. Qualitative interpretation clarified why these patterns occurred, revealing how beliefs, emotions, social norms, and contextual constraints interacted in lived experiences. Importantly, integration exposed key tensions not evident in single-method analyses—such as high levels of factual HIV knowledge coexisting with low uptake of testing or biomedical prevention, and perceived reductions in sexual pleasure overriding risk awareness in condom use decisions. This combined perspective shifted the analytic focus from isolated determinants to interrelated behavioral mechanisms, helping distinguish surface-level knowledge gaps from deeper motivational and contextual barriers. As a result, the mixed-methods integration informed not only the identification of priority determinants, but also the selection of intervention strategies aligned with how individuals interpret, negotiate, and act upon sexual health information in real-world contexts.

### Strategy development and behavioral mapping

Based on the triangulated findings, we developed a set of actionable, theory-informed strategies to address the key behavioral determinants identified. Table 5 presents the mapping of representative barriers and enablers to corresponding COM-B components, TDF domains, BCW intervention functions, and recommended Behavior Change Techniques (BCTs) based on the BCT Taxonomy v1. When relevant, we specify whether the strategy targets condom use, HIV/STI testing, or both. Each row represents a prioritized behavioral determinant identified through the triangulated analysis of survey data and narrative synthesis. Strategies are tailored to either condom use or HIV/STI testing behaviors and aligned with the Chilean public health context.

For example, the belief that condoms reduce pleasure (classified as a major barrier under Beliefs about Consequences) was addressed through Persuasion, using BCTs such as 5.1 Information about health consequences and 9.1 Credible source. In contrast, low perceived risk of HIV/STI (mapped to goals and reflective motivation) was targeted through education and modeling strategies.

This final mapping serves as a blueprint for culturally adapted, behaviorally grounded interventions to promote condom use and HIV/STI testing. It bridges theoretical diagnosis and applied strategy, fulfilling the objectives of the mixed-methods approach.

**Table 5:** Linking prioritized behavioral determinants to COM-B, TDF domains, intervention functions, BCTs, and suggested strategies for condom use and HIV/STI testing.

Prioritized barrier/enabler	Target behavior	COM-B component	TDF domain(s)	Intervention function(s) BCW	BCT(s) (Examples)	Suggested strategy (Chile)
Belief that condoms reduce sexual pleasure (more frequently reported by men than women)	Condom Use	Reflective Motivation	Beliefs about Consequences	Education, Persuasion	5.1 Information about Health Consequences; 9.1 Credible Source	Develop culturally resonant campaigns using peer educators to explain how sex can be pleasurable with condom use
Fear of partner judgment or rejection when suggesting condom use	Condom Use	Social Opportunity	Social Influences	Enablement, Modeling	3.1 Social Support (unspecified); 6.1 Demonstration of Behavior	Train youth influencers to share information about condom use in relationships through videos or workshops
Low perceived susceptibility to HIV/STIs	HIV/STI testing	Reflective Motivation	Beliefs about Consequences	Education, Persuasion	5.1 Information about Health Consequences; 9.2 Pros and Cons	Target misconceptions about not being at risk in youth-focused digital content
Lack of knowledge on where to get tested	HIV/STI testing	Physical Opportunity	Environmental Context and Resources	Environmental Restructuring, Education	12.1 Restructuring the Physical Environment; 4.1 Instruction on How to Perform the Behavior	Create geolocated mobile resources indicating testing centers and walk-in sites
Limited self-efficacy to negotiate condom use	Condom Use	Psychological Capability	Beliefs about Capabilities	Training, Enablement	1.4 Action Planning; 1.2 Problem Solving	Implement school-based role play activities to improve condom negotiation skills
Social norms that undermine condom use in steady relationships	Condom Use	Social Opportunity	Social Influences	Modeling, Persuasion	6.2 Social Comparison; 13.2 Framing/Reframing	Promote positive narratives of condom use within long-term relationships via testimonials or media
Perception that testing is only for symptomatic people or certain groups	HIV/STI testing	Reflective Motivation	Beliefs about Consequences	Education, Persuasion	5.6 Information about Emotional Consequences; 9.1 Credible Source	Develop messages that promote routine testing for everyone as normal, regardless of symptoms or group
Perceived high cost or time investment for testing	HIV/STI testing	Physical Opportunity	Environmental Context and Resources	Enablement, Environmental Restructuring	12.5 Adding Objects to the environment; 7.1 Prompts/Cues	Provide self-testing kits with clear instructions in pharmacies or community spaces
Feeling judged or stigmatized when using testing services	HIV/STI testing	Social Opportunity	Social/Professional Role and Identity; Social Influences	Environmental Restructuring, Enablement	11.2 Reduce Negative Emotions; 13.3 Incompatible Beliefs	Train health personnel in stigma-free communication and create welcoming, youth-friendly spaces for testing
High knowledge of condom effectiveness and access (facilitator)	Condom Use	Psychological Capability	Knowledge	Education	4.1 Instruction on How to Perform the Behavior; 2.2 Feedback on Behavior	Strengthen condom promotion in health curricula and social media with accurate, empowering content

COM-B = Capability, Opportunity, Motivation – Behavior; TDF = Theoretical Domains Framework; BCW = Behavior Change Wheel; BCT = Behavior Change Technique.

## Discussion

This study provides a theory-informed, mixed-methods interpretation of the behavioral determinants shaping condom use and HIV/STI testing among Chilean adults, using nationally representative survey data. By applying the COM-B model, the TDF, and the Behavior Change Wheel, we identified distinct yet overlapping patterns of barriers and enablers across both preventive behaviors, highlighting how knowledge, access to services, emotional responses, intentions, and social norms jointly influence sexual health practices.

Overall, the findings indicate that condom use is more strongly constrained by motivational, emotional, and social barriers, whereas HIV/STI testing is more often facilitated by knowledge-related factors but remains limited by structural and motivational constraints. Rather than reflecting simple information deficits, these patterns highlight persistent intention–behavior gaps and contextual barriers that help explain why preventive behaviors remain suboptimal despite relatively high awareness of HIV and STI risks. The sections below situate these behavior-specific findings within the broader literature and regional context. To our knowledge, this is the first study to apply the COM-B model and the TDF to a nationally representative dataset in Latin America to systematically identify behavioral determinants of condom use and HIV/STI testing.

### Behavioral determinants of condom use in context

Barriers to condom use were most commonly associated with beliefs about reduced pleasure, trust in stable relationships, and inconsistent behavioral regulation. These findings align with prior evidence indicating that emotional discomfort and normative expectations reduce condom uptake in long-term partnerships^36–38^. The role of sex education and early socialization emerged as a major structural gap, consistent with Latin American studies highlighting insufficient curricula and little family discussion about sexuality^4^. These limitations are further supported by findings from Armayones-Ruiz et al.^3^, who emphasized the need for campaigns to move beyond traditional prevention narratives. Using the COM-B and Behavior Change Wheel frameworks, they demonstrated that co-designed interventions incorporating education, persuasion, and training components were perceived as more acceptable and effective than traditional prevention campaigns in promoting safe sex behaviors among Chilean university students. Notably, although respondents expressed a desire to prevent HIV/STIs, this rarely translated into a consistent practice, a disconnect observed in similar behavioral studies across the region. A recent overview of systematic reviews further confirmed that, despite improvements in knowledge and self-efficacy, digital behavioral interventions often fail to produce sustained condom use, likely due to variability in design and inadequate application of behavior change frameworks^39,40^.

Similar patterns have been reported across Latin America, where condom use is frequently constrained by social norms, limited sexual communication, and perceptions of reduced pleasure. Studies from Chile and neighboring countries indicate that insufficient early sex education and persistent gender norms continue to shape condom-related decision-making well into adulthood^41^. These convergent findings suggest that the barriers identified in this study reflect broader regional patterns rather than context-specific anomalies.

### Behavioral determinants of HIV/STI testing in context

Testing behavior was facilitated by strong foundational knowledge of HIV transmission and condom efficacy. These items reflected strong awareness of HIV transmission routes and motivations for preventing STIs, consistent with global health literacy surveys^2^. However, low awareness of PrEP and structural barriers—such as access and time constraints—continue to undermine routine testing. Emotional and interpersonal barriers, including stigma and anticipated regret, also mirrored global patterns observed in populations with limited access to supportive health systems. The high willingness to disclose STI diagnoses suggests social responsibility may be leveraged in future interventions.

Consistent with our findings, studies from Central and South America have documented that HIV testing behaviors are strongly influenced by structural access barriers, stigma, and social norms rather than by knowledge alone. For example, research among key populations in El Salvador and Brazil has highlighted how limited service availability, fear of disclosure, and gendered expectations constrain testing uptake, despite relatively high awareness of HIV transmission^42,43^. These parallels reinforce the relevance of addressing opportunity- and motivation-related determinants when designing HIV/STI testing interventions in Latin American settings. Taken together, these comparisons suggest that the behavioral determinants identified through COM-B and TDF mapping in this national Chilean survey align closely with patterns observed across diverse Latin American contexts, underscoring the broader applicability of theory-informed behavioral diagnosis for HIV/STI prevention.

### Cross-cutting determinants

Two theoretical domains consistently emerged across both behaviors: Behavioral Regulation and Environmental Context and Resources. These findings suggest that interventions that target planning, habit formation, and service access may improve both condom use and testing. Moreover, though behavior-specific, emotional and social dimensions represent key leverage points for future interventions.

Importantly, several contextual indicators from the national survey—such as the absence of family-based sexual communication (71.2%), poor evaluations of sex education (over 50%), and limited partner discussion prior to first sex (33.4%)—highlight structural influences that were not formally coded as behavioral determinants but are likely to shape norms and motivation. These factors provide critical background for understanding the persistence of behavioral gaps despite high baseline knowledge.

### Cultural context and behavioral determinants

The identified behavioral determinants should be interpreted in light of Chile’s sociocultural context. Chile has a strong Catholic cultural heritage and traditionally conservative norms around sexuality, which have historically shaped sex education, family communication, and attitudes toward pleasure and risk. These norms may contribute to limited early sexual communication within families, discomfort discussing sexuality, and moralized views of condom use—patterns reflected in barriers related to perceived pleasure, social influences, and behavioral regulation. Additionally, gender norms emphasizing male sexual initiative and female responsibility for contraception may partly explain asymmetric perceptions of risk, negotiation difficulties, and inconsistent condom use, even within stable relationships. Together, these contextual factors likely interact with individual knowledge and motivation, reinforcing structural and interpersonal barriers to condom use and HIV/STI testing observed in this study.

### Strengths and limitations

To our knowledge, this is the first study that systematically applies the COM-B model and TDF to a nationally representative health survey in Latin America to assess behavioral determinants of STI prevention. The mixed-methods design added explanatory power and depth to the quantitative findings.

Nonetheless, limitations include the cross-sectional nature of the data, which precludes causal inference, and the reliance on self-reported behaviors, which may be influenced by recall and social desirability biases. Additionally, though informed by theory, the classification thresholds used to determine barriers and enablers may require validation in other contexts. As in prior COM-B–informed survey studies, the cut-offs used to classify items into enablers or barriers should be interpreted as heuristic tools to support synthesis and prioritization, rather than definitive thresholds, and results should therefore be interpreted with appropriate caution. Importantly, key conclusions were supported by consistent patterns across multiple items and domains, reducing reliance on any single threshold decision.

As with most population-based sexual health surveys, reliance on self-reported behaviors may be affected by recall bias and social desirability, particularly for sensitive outcomes such as condom use, HIV testing, and sexual communication. These biases may lead to underreporting of risk behaviors or overreporting of preventive practices. While theory-informed mapping (COM-B and TDF) does not eliminate these biases—and may, in some cases, reflect participants’ post hoc rationalizations—it can also mitigate interpretive bias by organizing responses into conceptually coherent domains and emphasizing consistent patterns across multiple items rather than relying on single indicators.

### Public health implications for research, practice, and policy

#### Implications for research

- Future studies should build on this approach by conducting subgroup analyses (e.g., by gender, age, and sexual orientation) to explore differential behavioral determinants.
- There is a need to empirically test interventions targeting the specific COM-B and TDF-informed barriers and enablers identified in this study.
- Extending behavioral diagnosis frameworks to other protective health behaviors captured in national surveys may further enhance their analytical and policy value.

#### Implications for practice

- Health promotion strategies should move beyond cognitive-based education to incorporate emotional and interpersonal skill-building components.
- Tailored messaging is needed for individuals in stable relationships who may underestimate their risk of HIV/STIs.
- Public health practice should actively promote communication and mutual disclosure within sexual partnerships as part of routine prevention.

#### Policy implications for national HIV/STI prevention

From a policy perspective, these findings highlight several actionable priorities for strengthening national HIV/STI prevention strategies in Chile.

- National sexual education policies should strengthen curricula to address not only knowledge gaps but also emotional, relational, and behavioral dimensions of sexual health.
- The lack of early sexual communication within families highlights upstream structural deficits that may be addressed through curriculum reform, educator training, and family-based interventions.
- Expanding access to HIV/STI testing through community-based services, digital platforms, and self-testing modalities should be prioritized.
- National prevention strategies would benefit from systematically adopting behavioral science frameworks to guide program design, implementation, and evaluation.

#### Future directions

Future research should examine these findings in key subpopulations, such as LGBTQ+ communities and adolescents, where distinct determinants may emerge. In Chile, longitudinal studies and experimental trials testing COM-B-informed interventions are warranted. Furthermore, efforts to expand awareness and access to PrEP and integrate emotional support into testing pathways may prove particularly impactful.

## Conclusion

This study applied a theory-driven mixed-methods approach to identify and classify behavioral determinants of condom use and HIV/STI testing among Chilean adults using a nationally representative survey. By integrating quantitative classification with theory-informed qualitative interpretation, the study provides a comprehensive behavioral diagnosis of the psychological, social, and structural factors shaping HIV/STI prevention behaviors in a real-world population context.

Rather than focusing solely on knowledge deficits, the findings demonstrate that prevention behaviors are strongly influenced by emotional responses, relationship dynamics, social norms, and access-related constraints. The systematic application of the COM-B model, the TDF, and the Behavior Change Wheel enabled the translation of population-level survey data into actionable, theory-based intervention targets.

This behavioral diagnosis lays the foundation for designing culturally relevant and theoretically grounded HIV/STI prevention interventions in Chile. By extending COM-B and TDF–based analysis to a nationally representative population survey, this study demonstrates the feasibility, scalability, and added value of applying behavioral science frameworks within routine public health data systems in Latin America, with clear implications for future research, policy, and program design in similar settings.

## Supplementary Information

Below is the link to the electronic supplementary material.

Supplementary Material 1

## Data Availability

All data analyzed in this study are publicly available through the ENSSEX 2022–2023 national survey [https://datos.gob.cl/dataset/encuesta-nacional-de-salud-sexualidad-y-genero-enssex-2022-2023/resource/ed81f50c-1c7d-43d9-9083-dfc161e0cd66] . No additional data were generated.

## Abbreviations

AACTT: Action, Actor, Context, Target, Time
BCT: Behavior change technique
BCTT_v_1: Behavior change technique taxonomy version 1
BCW: Behavior change wheel
COM-B: Capability, opportunity, motivation - behavior
DBCI: Digital behavior change intervention
ENSSEX: Chilean national health, sexuality, and gender survey
FBM: Fogg behavior model
HIV: Human immunodeficiency virus
LGBTQ+: Lesbian, gay, bisexual, transgender, queer, and other sexual orientations and gender identities
MMARS: Mixed methods article reporting standards
P_r_EP: Pre-exposure prophylaxis
STI: Sexually transmitted infection
TDF: Theoretical domains framework

## References

[1] World Health Organization. Sexually transmitted infections (STIs). World Health Organization. https://www.who.int/news-room/fact-sheets/detail/sexually-transmitted-infections-(stis). (2024).

[2] UNAIDS. UNAIDS report shows that upholding human rights is vital for ending the AIDS pandemic | UNAIDS. https://www.unaids.org/en/resources/presscentre/pressreleaseandstatementarchive/2024/november/20241126_world-aids-day-report (2024).

[3] Armayones Ruiz, M. et al. Barriers and facilitators for safe sex behaviors in students from universidad de Santiago de Chile (USACH) through the COM-B model. *BMC Public. Health*. **23** (1), 677. 10.1186/s12889-023-15489-y (2023). 37041528 10.1186/s12889-023-15489-y PMC10088188

[4] Duarte-Anselmi, G., Leiva-Pinto, E., Vanegas-López, J. & Thomas-Lange, J. Experiences and perceptions on sexuality, risk and STI/HIV prevention campaigns by university students. Designing a digital intervention. *Cienc. Saude Coletiva*. **27** (3), 909–920. 10.1590/1413-81232022273.05372021 (2022). 10.1590/1413-81232022273.05372021 35293468

[5] World Health Organization WHO. Biobehavioral survey guidelines for populations at risk for HIV. Published online 2017. https://stacks.cdc.gov/view/cdc/50667 (2024).

[6] Duarte, G. et al. Introduction to behavioral science and its practical applications in public health. *Medwave* **25** (1), e3017. 10.5867/medwave.2025.01.3017 (2025). 39836869 10.5867/medwave.2025.01.3017

[7] Duarte-Anselmi, G., Crane, S. M., Ruiz, M. A. & Dintrans, P. V. Behavioral science meets public health: a scoping review of the fogg behavior model in behavior change interventions. *BMC Public. Health*. **25** (1), 3468. 10.1186/s12889-025-24525-y (2025). 41088011 10.1186/s12889-025-24525-y PMC12522219

[8] Michie, S., van Stralen, M. M. & West, R. The behaviour change wheel: A new method for characterising and designing behaviour change interventions. *Implement. Sci.* **6** (1), 42. 10.1186/1748-5908-6-42 (2011). 21513547 10.1186/1748-5908-6-42 PMC3096582

[9] Cane, J., O’Connor, D. & Michie, S. Validation of the theoretical domains framework for use in behaviour change and implementation research. *Implement. Sci.* **7** (1), 37. 10.1186/1748-5908-7-37 (2012). 22530986 10.1186/1748-5908-7-37 PMC3483008

[10] Michie, S., Atkins, L. & West, R. *The Behaviour Change Wheel Book - A Guide To Designing Interventions*. http://www.behaviourchangewheel.com/ (2014).

[11] Anselmi, G. D. et al. Overview of systematic reviews on behavioral determinants of physical activity and healthy eating in schoolchildren. *Sci. Rep.* **15** (1), 35379. 10.1038/s41598-025-19332-9 (2025). 41068188 10.1038/s41598-025-19332-9 PMC12511304

[12] Atkins, L. et al. A guide to using the Theoretical Domains Framework of behaviour change to investigate implementation problems. *Implement. Sci.* **12** (1), 77. 10.1186/s13012-017-0605-9 (2017). 28637486 10.1186/s13012-017-0605-9 PMC5480145

[13] Presseau, J. et al. Action, actor, context, target, time (AACTT): a framework for specifying behaviour. *Implement. Sci.* **14** (1), 102. 10.1186/s13012-019-0951-x (2019). 31806037 10.1186/s13012-019-0951-x PMC6896730

[14] Chater, A. M. et al. Influences on nurses’ engagement in antimicrobial stewardship behaviours: a multi-country survey using the Theoretical Domains Framework. *J. Hosp. Infect.* **129**, 171–180. 10.1016/j.jhin.2022.07.010 (2022). 35843415 10.1016/j.jhin.2022.07.010

[15] Ministerio de Salud de Chile. Encuesta Nacional de Salud, Sexualidad y Género (ENSSEX) 2022–2023 - ENSSEX 2022–2023 - Portal de Datos Abiertos. https://datos.gob.cl/dataset/encuesta-nacional-de-salud-sexualidad-y-genero-enssex-2022-2023/resource/ed81f50c-1c7d-43d9-9083-dfc161e0cd66 (2024).

[16] Anguera, M. T., Portell, M., Chacón-Moscoso, S. & Sanduvete-Chaves, S. Indirect Observation in Everyday Contexts: Concepts and Methodological Guidelines within a Mixed Methods Framework. *Front. Psychol.* **9**10.3389/fpsyg.2018.00013 (2018). 10.3389/fpsyg.2018.00013 PMC5797623 29441028

[17] Clark, V. L. P. & Ivankova, N. V. *Mixed Methods Research: A Guide to the Field* (SAGE, 2016).

[18] Creswell, J. W. & Clark, V. L. P. *Designing and Conducting Mixed Methods Research* (SAGE, 2011).

[19] Huijg, J. M., Gebhardt, W. A., Crone, M. R., Dusseldorp, E. & Presseau, J. Discriminant content validity of a theoretical domains framework questionnaire for use in implementation research. *Implement. Sci.* **9** (1), 11. 10.1186/1748-5908-9-11 (2014). 24423394 10.1186/1748-5908-9-11 PMC3896680

[20] Tashakkori, A. & Teddlie, C. *Mixed Methodology: Combining Qualitative and Quantitative Approaches* (SAGE, 1998).

[21] Levitt, H. M. et al. Journal article reporting standards for qualitative primary, qualitative meta-analytic, and mixed methods research in psychology: The APA Publications and Communications Board task force report. *Am. Psychol.* **73** (1), 26–46. 10.1037/amp0000151 (2018). 29345485 10.1037/amp0000151

[22] McKinlay, A. R. et al. Theoretical mapping of the barriers and enablers to having blood pressure checked among adults without a hypertension diagnosis: a systematic review and theoretical synthesis using behaviour change frameworks. *Health Psychol. Rev.* **0** (0), 1–31. 10.1080/17437199.2025.2485094 (2025). 10.1080/17437199.2025.2485094 40237390

[23] Shapoval, V. et al. Barriers to Deprescribing Benzodiazepines in Older Adults in a Survey of European Physicians. *JAMA Netw. Open.* **8** (3), e2459883. 10.1001/jamanetworkopen.2024.59883 (2025). 40029661 10.1001/jamanetworkopen.2024.59883 PMC11877185

[24] Leech, N. L. & Onwuegbuzie, A. J. Guidelines for Conducting and Reporting Mixed Research in the Field of Counseling and Beyond. *J. Couns. Dev.* **88** (1), 61–69. 10.1002/j.1556-6678.2010.tb00151.x (2010).

[25] Heidari, S., Babor, T. F., De Castro, P., Tort, S. & Curno, M. Sex and Gender Equity in Research: rationale for the SAGER guidelines and recommended use. *Res. Integr. Peer Rev.* **1** (1), 2. 10.1186/s41073-016-0007-6 (2016). 29451543 10.1186/s41073-016-0007-6 PMC5793986

[26] Byrt, T., Bishop, J. & Carlin, J. B. Bias, prevalence and kappa. *J. Clin. Epidemiol.* **46** (5), 423–429. 10.1016/0895-4356(93)90018-V (1993). 8501467 10.1016/0895-4356(93)90018-v

[27] Viera, A. J. & Garrett, J. M. Understanding interobserver agreement: the kappa statistic. *Fam Med.* **37** (5), 360–363 (2005). 15883903

[28] Jamieson, S. Likert scales: how to (ab)use them. *Med. Educ.* **38** (12), 1217–1218. 10.1111/j.1365-2929.2004.02012.x (2004). 15566531 10.1111/j.1365-2929.2004.02012.x

[29] Carifio, J. & Perla, R. Resolving the 50-year debate around using and misusing Likert scales. *Med. Educ.* **42** (12), 1150–1152. 10.1111/j.1365-2923.2008.03172.x (2008). 19120943 10.1111/j.1365-2923.2008.03172.x

[30] Norman, G. Likert scales, levels of measurement and the laws of statistics. *Adv. Health Sci. Educ.* **15** (5), 625–632. 10.1007/s10459-010-9222-y (2010). 10.1007/s10459-010-9222-y 20146096

[31] DeCastellarnau, A. A classification of response scale characteristics that affect data quality: a literature review. *Qual. Quant.* **52** (4), 1523–1559. 10.1007/s11135-017-0533-4 (2018). 29937582 10.1007/s11135-017-0533-4 PMC5993837

[32] Sullivan, G. M. & Artino, A. R. Jr Analyzing and Interpreting Data From Likert-Type Scales. *J. Grad Med. Educ.* **5** (4), 541–542. 10.4300/JGME-5-4-18 (2013). 24454995 10.4300/JGME-5-4-18 PMC3886444

[33] Keyworth, C., Epton, T., Goldthorpe, J., Calam, R. & Armitage, C. J. Acceptability, reliability, and validity of a brief measure of capabilities, opportunities, and motivations (COM-B). *Br. J. Health Psychol.* **25** (3), 474–501. 10.1111/bjhp.12417 (2020). 32314500 10.1111/bjhp.12417

[34] Michie, S. et al. The Behavior Change Technique Taxonomy (v1) of 93 Hierarchically Clustered Techniques: Building an International Consensus for the Reporting of Behavior Change Interventions. *Ann. Behav. Med.* **46** (1), 81–95. 10.1007/s12160-013-9486-6 (2013). 23512568 10.1007/s12160-013-9486-6

[35] Castro, O. et al. Translating the behaviour change technique taxonomy version 1 into Spanish: Methodology and validation. *Wellcome Open. Res.* **9**, 298. 10.12688/wellcomeopenres.21388.1 (2024). 39323609 10.12688/wellcomeopenres.21388.1 PMC11422758

[36] Higgins, J. A. & Wang, Y. The Role of Young Adults’ Pleasure Attitudes in Shaping Condom Use. *Am. J. Public. Health*. **105** (7), 1329–1332. 10.2105/AJPH.2015.302567 (2015). 25973832 10.2105/AJPH.2015.302567 PMC4458205

[37] Lindberg, L. D., Bell, D. L. & Kantor, L. M. The Sexual and Reproductive Health of Adolescents and Young Adults During the COVID-19 Pandemic. *Perspect. Sex. Reprod. Health*. **52** (2), 75–79. 10.1363/psrh.12151 (2020). 32537858 10.1363/psrh.12151 PMC7323157

[38] Marston, C. & King, E. Factors that shape young people’s sexual behaviour: a systematic review. *Lancet* **368** (9547), 1581–1586. 10.1016/S0140-6736(06)69662-1 (2006). 17084758 10.1016/S0140-6736(06)69662-1

[39] Duarte-Anselmi, G., Sanduvete-Chaves, S., Chacón-Moscoso, S. & López-Arenas, D. Behavioral determinants and effectiveness of digital interventions for STI/HIV prevention: an overview of systematic reviews. *Preprint posted*. 10.2196/preprints.74201 (2025). 10.2196/74201 PMC12902757 41610417

[40] Duarte, G., Sanduvete-Chaves, S., López-Arenas, D. & Chacón-Moscoso, S. Digital strategies and behavior change techniques for preventing sexually transmitted infections: Protocol for an overview of systematic reviews. *Medwave* **25** (2), e3020. 10.5867/medwave.2025.02.3020 (2025). 40063926 10.5867/medwave.2025.02.3020

[41] Olivera, M. P., Salinas-Oñate, N. & de la Hoz, S. [Condom use among young Chileans: The role of social determinants, gender roles and mental health]. *Rev. Med. Chil.* **151** (10), 1309–1318. 10.4067/s0034-98872023001001309 (2023). 39093135 10.4067/s0034-98872023001001309

[42] Paz-Bailey, G. et al. How many men who have sex with men and female sex workers live in El Salvador? Using respondent-driven sampling and capture–recapture to estimate population sizes. *Sex. Trans. Infect.* **87** (4), 279–282. 10.1136/sti.2010.045633 (2011). 21385892 10.1136/sti.2010.045633

[43] de Freitas Vale, J. et al. Access to HIV testing and factors associated among sexual minority women in a metropolitan region of the Brazilian Amazon. *Sci. Rep.* **15** (1), 3176. 10.1038/s41598-025-87580-w (2025). 39863757 10.1038/s41598-025-87580-w PMC11762723

