# Supplementary material for: Behavioral determinants of condom use and HIV/STI testing in Chile: a theory-driven mixed-methods study
Source: Sci Rep. 2026 Mar 6;16:12290. doi: 10.1038/s41598-026-43017-6 (PMC13079757; doi:10.1038/s41598-026-43017-6)
Supplement: Supplementary file 1 — Supplementary Material 1 [file 41598_2026_43017_MOESM1_ESM.docx]

**Behavioral Determinants of Condom Use and HIV/STI Testing in Chile: A Theory-Driven Mixed-Methods Study-Multimedia Appendices**

Contents

[Appendix 1 – JARS–Mixed | Table S1. Compliance with the Mixed Methods Article Reporting Standards (MMARS) 1](#_Toc218502382)

[Appendix 2. Sex and Gender Equity in Research SAGER Guidelines 2](#_Toc218502383)

[Appendix 3 – Structured Coding Matrix of Selected ENSSEX Survey Items: Variable Labels, Theoretical Mapping, and Justification 3](#_Toc218502384)

[Appendix 4 –. Final Coding Matrix: Mapping of ENSSEX Survey Items to TDF, and COM-B Frameworks 12](#_Toc218502385)

[Appendix 5 – Intercoder Reliability Calculation Report 16](#_Toc218502386)

[Appendix 6. Supplementary Material – Structured Quantitative Analysis 17](#_Toc218502387)

[Appendix 7. 405 items Included in the National Survey 62](#_Toc218502388)

[Appendix 8 – Supplementary Table X. Full Classification of Survey Items by COM-B, TDF Domains, Response Thresholds, and Behavioral Outcomes 76](#_Toc218502389)

[Appendix 9 – Supplementary Table S2. Classification of Analyzed vs. Contextual Variables 78](#_Toc218502390)

[Appendix Supplementary Table S1 78](#_Toc218502391)

[Appendix Supplementary Table S2 83](#_Toc218502392)

[Appendix Supplementary Table S3 84](#_Toc218502393)

Appendix 1 – JARS–Mixed | Table S1. Compliance with the Mixed Methods Article Reporting Standards (MMARS)

| **JARS–Mixed \| Table S1. Mixed Methods Article Reporting Standards (MMARS)  Information Recommended for Inclusion in Manuscripts That Report the Collection and Integration of Qualitative and Quantitative Data (Levitt et al., 2018)** | | | | |
| --- | --- | --- | --- | --- |
|  | **Standard** | **Reported** | **Location in Manuscript** | **Location Page #** |
| **Title Page** | Clearly identify the article as reporting a mixed methods study | Yes | Title, Abstract | #1 |
| ***Abstract*** | Specify design type, data sources, analytic approach, and main findings | Yes | Abstract | #2 |
| ***Introduction*** | State the research problem and rationale for using mixed methods | Yes | Introduction | #3 |
| ***Introduction*** | Describe qualitative, quantitative, and mixed-methods goals | Yes | Research Questions | #4 |
| ***Method*** |  |  |  |  |
| ***Research Design Overview*** | Define and justify the mixed methods design | Yes | Methods - Study Design | #5 |
| ***Participants or Other Data Sources*** | Describe qualitative and quantitative data sources and how they relate | Yes | Data Source | #6 |
| ***Participant Recruitment Participant Sampling or Selection*** | Describe participant selection separately for each method | Yes | Participant Characteristics | #6 and #10 |
| ***Data Analysis*** | Describe analyses for each method and how results were integrated | Yes | Analysis | #6-8 and supplementary appendices 4-7 |
| ***Validity, Reliability, and Methodological Integrity*** | Describe validity, reliability, and integration quality | Yes | Methodological Rigor Section | #8 and 9 |
| ***Findings/Results*** | Present findings from each method and integration strategy | Yes | Results - Quantitative, Qualitative, Triangulation | #9-20 |
| ***Discussion*** | Interpret integrated findings and discuss design implications | Yes | Discussion - Implications | #21 |

From: Levitt, H. M., Bamberg, M., Creswell, J. W., Frost, D. M., Josselson, R., & Suárez-Orozco, C. (2018). Journal article reporting standards for qualitative primary, qualitative meta-analytic, and mixed methods research in psychology: The APA Publications and Communications Board task force report. American Psychologist, 73(1), 26–46. <https://doi.org/10.1037/amp0000151>

Appendix 2. Sex and Gender Equity in Research SAGER Guidelines

| Recommendations per section of the article | | Page # |
| --- | --- | --- |
| Title and abstract | If only one sex is included in the study, or if the results of the study are to be applied to only one sex or gender, the title and the abstract should specify the sex of animals or any cells, tissues and other material derived from these and the sex and gender of human participants. | NA, all genders included |
| Introduction | Authors should report, where relevant, whether sex and/or gender differences may be expected. | NA, all genders included |
| Methods | Authors should report how sex and gender were taken into account in the design of the study, whether they ensured adequate representation of males and females, and justify the reasons for any exclusion of males or females. | 6 |
| Results | Where appropriate, data should be routinely presented disaggregated by sex and gender. Sex- and gender-based analyses should be reported regardless of positive or negative outcome. In clinical trials, data on withdrawals and dropouts should also be reported disaggregated by sex. | 9 |
| Discussion | The potential implications of sex and gender on the study results and analyses should be discussed. If a sex and gender analysis was not conducted, the rationale should be given. Authors should further discuss the implications of the lack of such analysis on the interpretation of the results. | 21 |

From: (Heidari S, Babor TF, De Castro P, Tort S, Curno M. Sex and Gender Equity in Research: rationale for the SAGER guidelines and recommended use. Research Integrity and Peer Review. 2016;1: 2. doi:10.1186/s41073-016-0007-6)

Appendix 3 – Structured Coding Matrix of Selected ENSSEX Survey Items: Variable Labels, Theoretical Mapping, and Justification

| **N°** | **VARIABLE** | **Label ( questionnaire Item)** | **Justification** | **Coder 1 vote TDF** | **Coder 2 vote TDF** |
| --- | --- | --- | --- | --- | --- |
| 1 | p1 | ¿Cuál es su sexo asignado al nacer? [What is your sex assigned at birth?] | Do not code. Category does not meet the objective. | Not applicable | Not applicable |
| 2 | p2 | ¿Es usted una persona intersex? [Are you an intersex person?] | Do not code. Category does not meet the objective. | Not applicable | Not applicable |
| 3 | p3 | ¿Cuál es el género con el que Usted se identifica? [What gender do you identify with?] | Do not code. Category does not meet the objective. | Not applicable | Not applicable |
| 4 | p4 | ¿Qué edad tiene? [How old are you?] | Do not code. Category does not meet the objective. | Not applicable | Not applicable |
| 5 | p5 | ¿Cuál es su nivel educacional más alto alcanzado o su nivel educacional actual? [What is your highest level of education attained or your current educational level?] | Do not code. Category does not meet the objective. | Not applicable | Not applicable |
| 6 | p7 | ¿Cuál es su estado conyugal o civil actual? [What is your current marital or civil status?] | Do not code. Category does not meet the objective. | Not applicable | Not applicable |
| 7 | p8 | ¿Cómo calificaría su calidad de vida? [How would you rate your quality of life?] | Do not code. Category does not meet the objective. | Not applicable | Not applicable |
| 8 | i_1_p9 | (Con la privacidad que tiene donde vive) Ahora le preguntaré acerca de cómo se s [Regarding the privacy where you live, I will now ask how you feel about it] | Do not code. Category does not meet the objective. | Not applicable | Not applicable |
| 9 | i_3_p9 | (Con su vida amorosa) Ahora le preguntaré acerca de cómo se [Regarding your love life, I will now ask how you feel about it] | Do not code. Category does not meet the objective. | Not applicable | Not applicable |
| 10 | i_4_p9 | (Con la cantidad de diversión que tiene en su vida) Ahora le preguntaré acerca d [Regarding the amount of fun you have in your life, I will now ask how you feel about it] | Do not code. Category does not meet the objective. | Not applicable | Not applicable |
| 11 | i_5_p9 | (Con su vida familiar) Ahora le preguntaré acerca de cómo se siente Usted en dis [Regarding your family life, I will now ask how you feel about it] | Do not code. Category does not meet the objective. | Not applicable | Not applicable |
| 12 | i_6_p9 | (Con su vida sexual) Ahora le preguntaré acerca de cómo se siente Usted en disti [Regarding your sex life, I will now ask how you feel about it] | Do not code. Category does not meet the objective. | Not applicable | Not applicable |
| 13 | p10 | En general Usted diría que su salud es... [In general, would you say your health is...] | Do not code. Category does not meet the objective. | Not applicable | Not applicable |
| 14 | p22 | ¿Me podría decir cuánto pesa aproximadamente actualmente? [Could you tell me approximately how much you currently weigh?] | Do not code. Category does not meet the objective. | Not applicable | Not applicable |
| 15 | p23 | ¿Me podría decir cuánto mide aproximadamente actualmente? [Could you tell me approximately how tall you are currently?] | Do not code. Category does not meet the objective. | Not applicable | Not applicable |
| 16 | i_1_p25 | (Cocaína) ¿Ha probado Ud. alguna de las siguientes sustancias alguna vez en su vida? [Have you ever tried any of the following substances? (Cocaine)] | Do not code. Category does not meet the objective. | Not applicable | Context and resources |
| 17 | i_1_p26 | (Cocaína) ¿Cuándo fue la última vez que consumió? [When was the last time you used it? (Cocaine)] | Do not code. Category does not meet the objective. | Not applicable | Context and resources |
| 18 | i_2_p25 | (Marihuana) ¿Ha probado Ud. alguna de las siguientes sustancias alguna vez en su vida? [Have you ever tried any of the following substances? (Marijuana)] | Do not code. Category does not meet the objective. | Not applicable | Context and resources |
| 19 | i_2_p26 | (Marihuana) ¿Cuándo fue la última vez que consumió? [When was the last time you used it? (Marijuana)] | Do not code. Category does not meet the objective. | Not applicable | Context and resources |
| 20 | i_3_p25 | (Drogas inyectables) ¿Ha probado Ud. alguna de las siguientes sustancias alguna vez en su vida? [Have you ever tried any of the following substances? (Injectable drugs)] | Do not code. Category does not meet the objective. | Not applicable | Context and resources |
| 21 | i_3_p26 | (Drogas inyectables) ¿Cuándo fue la última vez que consumió? [When was the last time you used it? (Injectable drugs)] | Do not code. Category does not meet the objective. | Not applicable | Context and resources |
| 22 | i_4_p25 | (Tranquilizantes, ansiolíticos o antidepresivo) ¿Ha probado Ud. alguna de las siguientes sustancias alguna vez en su vida? [Have you ever tried any of the following substances? (Tranquilizers, anxiolytics or antidepressants)] | Do not code. Category does not meet the objective. | Not applicable | Context and resources |
| 23 | i_4_p26 | (Tranquilizantes, ansiolíticos o antidepresivo) ¿Cuándo fue la última vez que consumió? [When was the last time you used it? (Tranquilizers, anxiolytics or antidepressants)] | Do not code. Category does not meet the objective. | Not applicable | Context and resources |
| 24 | i_5_p25 | (Alcohol) ¿Ha probado Ud. alguna de las siguientes sustancias alguna vez en su vida? [Have you ever tried any of the following substances? (Alcohol)] | Do not code. Category does not meet the objective. | Not applicable | Context and resources |
| 25 | i_5_p26 | ¿Cuándo fue la última vez que consumió? [When was the last time you used it? (Alcohol)] | Do not code. Category does not meet the objective. | Not applicable | Context and resources |
| 26 | i_6_p25 | (Alucinógenos, hongos) ¿Ha probado Ud. alguna de las siguientes sustancias alguna vez en su vida? [Have you ever tried any of the following substances? (Hallucinogens, mushrooms)] | Do not code. Category does not meet the objective. | Not applicable | Context and resources |
| 27 | i_6_p26 | (Alucinógenos, hongos) ¿Cuándo fue la última vez que consumió? [When was the last time you used it? (Hallucinogens, mushrooms)] | Do not code. Category does not meet the objective. | Not applicable | Context and resources |
| 28 | i_7_p25 | (Poppers) ¿Ha probado Ud. alguna de las siguientes sustancias alguna vez en su vida? [Have you ever tried any of the following substances? (Poppers)] | Do not code. Category does not meet the objective. | Not applicable | Context and resources |
| 29 | i_7_p26 | (Poppers) ¿Cuándo fue la última vez que consumió? [When was the last time you used it? (Poppers)] | Do not code. Category does not meet the objective. | Not applicable | Context and resources |
| 30 | i_1_p33 | (Usar preservativos o condón disminuye el placer de las mujeres.) ¿Qué tan de acuerdo está con esta afirmación? [Using condoms decreases women's pleasure. How much do you agree with this statement?] | Investigates perceived barriers related to the impact of condom use on female sexual pleasure. | Beliefs about consequences | Beliefs about consequences |
| 31 | i_2_p33 | (Usar preservativos o condón disminuye el placer de los hombres.) ¿Qué tan de acuerdo está con esta afirmación? [Using condoms decreases men's pleasure. How much do you agree with this statement?] | Evaluates how condom use can be perceived as a barrier in male sexual experience. | Beliefs about consequences | Beliefs about consequences |
| 32 | i_3_p33 | (Es necesario ocupar preservativo o condón incluso si se tiene pareja estable) [It is necessary to use condoms even if you have a stable partner.] | Measures knowledge about the relevance of condom use in STI prevention, even among stable couples. | Knowledge | ~~Social Role & Identity~~ |
| 33 | i_4_p33 | (Usar preservativo o condón estimula el juego sexual.) [Using condoms enhances sexual play.] | Explores beliefs linking condom use with more positive sexual experiences. | Beliefs about consequences | Beliefs about consequences |
| 34 | i_5_p33 | (Los preservativos o condones son demasiado caros para usarlos regularmente) [Condoms are too expensive to use regularly.] | Analyzes economic barriers related to access and regular use of condoms. | Environmental context and resources | Context and resources |
| 35 | p34 | Cuando usted era niño/a, ¿En su familia se conversaban temas sexuales? [When you were a child, did your family talk about sexual topics?] | Examines the influence of early family socialization on sexuality and preventive practices. | Social influences | Social influences |
| 36 | p35 | (CONTESTAN P34=2,3) ¿Y con qué frecuencia usted participaba cuando se conversaban temas sexuales? [(If answered P34=2 or 3) How often did you participate in those conversations about sexual topics?] | Investigates the level of participation in family discussions related to sexuality and prevention. | Social influences | Social influences |
| 37 | t_p36_1 | En su escuela, cuándo usted era estudiante, ¿se impartía educación sexual en Enseñanza básica? [In your school, when you were a student, was sexual education provided in primary school?] | Evaluates the availability of formal sex education during primary education. | Environmental context and resources | Context and resources |
| 38 | t_p36_2 | En su escuela, cuándo usted era estudiante, ¿se impartía educación sexual en Enseñanza media? [In your school, when you were a student, was sexual education provided in secondary school?] | Investigates the continuity of sex education in higher educational levels. | Environmental context and resources | Context and resources |
| 39 | p37 | Y respecto de esa educación sexual, en general, usted cree que... [And regarding that sexual education, in general, you think that...] | Explores perceptions of the quality and impact of received sex education. | ~~Beliefs about consequences~~ | ~~Beliefs about capacity~~ |
| 40 | p38 | ¿Cómo evaluaría EN GENERAL la FORMACIÓN EN SEXUALIDAD que recibió en su colegio o escuela? [How would you rate, IN GENERAL, the SEXUALITY EDUCATION you received in your school?] | Evaluates general perceptions about sexuality education, without direct relation to specific behaviors. | ~~Beliefs about consequences~~ | ~~Beliefs about capacity~~ |
| 41 | i_2_p39 | (Métodos para prevenir infecciones de transmisión sexual) ¿Qué nota le pondría a.…? [Methods to prevent sexually transmitted infections: What grade would you give it?] | Measures knowledge level about STI preventive methods. | Knowledge | Knowledge |
| 42 | i_1_p40_o1 | Cuando usted era adolescente, a raíz de alguna duda sobre los siguientes temas, ¿A quién recurrió para resolverlas? Relaciones sexuales [When you were a teenager, if you had doubts about the following topics, who did you turn to? Sexual relations] | Identifies support networks available to resolve doubts about sexual relationships. | Environmental context and resources | Context and resources |
| 43 | i_3_p40_o1 | Cuando usted era adolescente, a raíz de alguna duda sobre los siguientes temas, ¿A quién recurrió para resolverlas? (Métodos preventivos de infecciones de transmisión sexual, como el VIH) [When you were a teenager, if you had doubts about the following topics, who did you turn to? (Preventive methods for sexually transmitted infections, such as HIV)] | Evaluates access to specific information about STI preventive methods. | Environmental context and resources | Context and resources |
| 44 | i_4_p41 | (Que enseñe a poner un preservativo o condón) [That teaches how to put on a condom] | Do not code. Category does not meet the objective. | Not applicable | Not applicable |
| 45 | p49 | ¿A qué edad tuvo su primera relación sexual voluntaria? (SI NO SABE LA EDAD EXACTA, SEÑALE EDAD APROXIMADA. ANOTAR LA EDAD DE LA PRIMERA RELACIÓN SEXUAL VOLUNTARIA) [At what age did you have your first voluntary sexual intercourse? (If you don't know the exact age, indicate an approximate age)] | Do not code. Category does not meet the objective. | Not applicable | Not applicable |
| 46 | p55 | Antes de su primera relación sexual, ¿Usted y esa persona hablaron de cómo evitar una ITS…? [Before your first sexual intercourse, did you and that person talk about how to avoid STIs...?] | Explores partner communication about STIs before first sexual intercourse. | Social influences | Social influences |
| 47 | p56 | En esa primera relación sexual, ¿Ustedes usaron algún método anticonceptivo? [In that first sexual intercourse, did you use any contraceptive method?] | Measures adoption of preventive practices in the first sexual intercourse. | Behavioral regulation | ~~Intentions~~ |
| 48 | p57 | ¿Cuál o cuáles métodos anticonceptivos usaron en esa primera relación sexual? MARQUE TODAS LAS QUE CORRESPONDA. [Which contraceptive methods did you use in that first sexual encounter? MARK ALL THAT APPLY.] | Records which specific contraceptive methods were used in the first sexual intercourse. | Behavioral regulation | ~~Intentions~~ |
| 49 | p58 | ¿Cuál fue el principal motivo por el cual usaron preservativo o condón? [What was the main reason for using a condom?] | Explores motivations for condom use in a preventive context. | ~~Reinforcement~~ | Goals |
| 50 | p59 | ¿Dónde obtuvo el método o los métodos mencionados anteriormente? [Where did you obtain the method(s) mentioned above?] | Investigates access to contraceptive methods within the social environment. | Environmental context and resources | Context and resources |
| 51 | p60 | ¿En qué lugar tuvo su primera relación sexual? [Where did you have your first sexual intercourse?] | Do not code. Category does not meet the objective. | Not applicable | Context and resources |
| 52 | p69 | En el transcurso de toda su vida, ¿podría indicar con cuántas personas ha tenido relaciones sexuales? [...] [Over your lifetime, how many people have you had sexual intercourse with? Approximate number.] | Do not code. Category does not meet the objective. | Not applicable | Not applicable |
| 53 | p73 | (CONTESTAN P71>0) En las relaciones con esas parejas sexuales del último año, ¿con qué frecuencia usted usaba condón o preservativo? [In relationships with those sexual partners in the past year, how often did you use a condom?] | Measures frequency of condom use in recent sexual relationships. | Behavioral regulation | ~~Intentions~~ |
| 54 | p89 | Y en la primera relación sexual que volvió a tener con (NOMBRE PAREJA SEPARACIÓN) después de la separación ¿usaron condón o preservativo? [In the first sexual intercourse after reuniting with (PARTNER'S NAME) post-separation, did you use a condom?] | Assesses condom use following significant changes in civil or emotional status. | Behavioral regulation | ~~Intentions~~ |
| 55 | p103 | (CONTESTAN SI P74 >0) Justo antes o al momento de las relaciones sexuales que ha tenido en el último mes, ¿usted consumió alguna de las siguientes sustancias? [Just before or during sexual intercourse in the last month, did you consume any of the following substances?] | Analyzes how substance use affects sexual decision-making. | ~~Emotion~~ | Reinforcement |
| 56 | p104 | (CONTESTAN SI P74 >0) Y alguna de las personas con la que usted estaba, ¿había consumido alguna de las siguientes sustancias? (MÚLTIPLE) [And any of the people you were with, had they consumed any of the following substances? (MULTIPLE)] | Explores how partner decisions influence the sexual context. | ~~Emotion~~ | Social influences |
| 57 | p119 | En esa última relación sexual, ¿Ustedes usaron alguno método anticonceptivo? [In that last sexual encounter, did you use any contraceptive method?] | Measures contraceptive use in recent sexual encounters. | Behavioral regulation | ~~Intentions~~ |
| 58 | p120 | ¿Cuál o cuáles métodos anticonceptivos utilizaron? MARQUE TODAS LAS QUE CORRESPONDAN [Which contraceptive methods did you use? MARK ALL THAT APPLY] | Records preventive methods used in recent sexual relationships. | Behavioral regulation | ~~Intentions~~ |
| 59 | p121 | (CONTESTAN P120=1,2) ¿Por qué motivo usaron preservativo o condón? [Why did you use a condom?] | Explores specific reasons behind condom use. | ~~Reinforcement~~ | Goals |
| 60 | p122 | ¿Dónde obtuvo el método o los métodos mencionados anteriormente? [Where did you obtain the method(s) mentioned above?] | Investigates access routes to preventive methods. | Environmental context and resources | Context and resources |
| 61 | p123 | (CONTESTAN SI P119=2) ¿Por qué razón no usó ningún método anticonceptivo? [Why didn’t you use any contraceptive method?] | Identifies perceived barriers to contraceptive use. | Beliefs about capabilities | ~~Goals~~ |
| 62 | p133 | Durante su vida, usted se ha sentido atraído/a sexualmente por… (ENCUESTADOR: SOLO PIDA NÚMERO DE RESPUESTA) [Throughout your life, who have you felt sexually attracted to...? (INTERVIEWER: ONLY REQUEST RESPONSE NUMBER)] | Do not code. Category does not meet the objective. | Not applicable | Not applicable |
| 63 | p134 | Usted actualmente se identifica cómo: [You currently identify as:] | Do not code. Category does not meet the objective. | Not applicable | Not applicable |
| 64 | p151 | (RESPONDE SEXO = MUJER) ¿Alguna vez ha ido a una consulta con un profesional de la salud por temas propios de ginecología, pubertad, sexualidad...? [For women: Have you ever consulted a health professional for personal topics such as gynecology, puberty, sexuality...?] | Investigates access to medical services related to sexuality (women). | Environmental context and resources | Context and resources |
| 65 | p152 | (RESPONDE SEXO = HOMBRE) ¿Alguna vez en su vida ha ido a una consulta o donde algún profesional de la salud para tratar asuntos médicos PROPIOS relacionados con la urología, pubertad, sexualidad, métodos preventivos del embarazo o infecciones de transmisión sexual? [MEN ONLY: Have you ever gone to a medical consultation or seen a health professional for PERSONAL medical issues related to urology, puberty, sexuality, contraceptive methods, or sexually transmitted infections?] | Investigates access to medical services related to sexuality (men). | Environmental context and resources | Context and resources |
| 66 | p154 | ¿Por qué motivo fue esa primera vez? (MARQUE TODAS LAS QUE CONSIDERE) [What was the reason for that first visit? (CHECK ALL THAT APPLY)] | Explores motivations for attending the first medical consultation. | ~~Environmental context and resources~~ | Goals |
| 67 | p155 | ¿A qué lugar fue? (LEA ALTERNATIVAS. CIRCULE SOLO UNO) [Where did you go? (READ OPTIONS. CIRCLE ONLY ONE)] | Assesses physical access to health services during the first consultation. | Environmental context and resources | Context and resources |
| 68 | p202 | ¿Alguna vez en su vida, un doctor o médico le ha dicho que tiene o que padece de...? [Has a doctor ever told you that you have or suffer from...?] | Do not code. Category does not meet the objective. | Not applicable | Not applicable |
| 69 | p203 | ¿A qué edad se lo diagnosticaron por última vez? [How old were you the last time you were diagnosed?] | Do not code. Category does not meet the objective. | Not applicable | Not applicable |
| 70 | p206 | ¿Cómo descubrió que tenía alguna de esas infecciones? Si ha tenido más de una vez una infección, piense en la última. [How did you discover that you had any of those infections? If you’ve had more than one, refer to the most recent.] | Explores how external circumstances, such as access to resources or services, influence STI discovery. | Environmental context and resources | Context and resources |
| 71 | p207 | La última vez que fue diagnosticado de alguna infección de transmisión sexual, ¿se lo informó a su o sus parejas sexuales? [The last time you were diagnosed with an STI, did you inform your partner(s)?] | Measures communication about diagnoses with sexual partners. | ~~Social influences~~ | Behavioral regulation |
| 72 | p208 | [RESPONDEN P202.10 = 2] Por cualquier razón, ¿Se ha hecho el examen del VIH o Sida en los últimos 12 meses? [[ONLY IF P202.10 = 2] For any reason, have you been tested for HIV/AIDS in the past 12 months?] | Assesses uptake of HIV preventive testing in recent months. | ~~Beliefs about consequences~~ | Behavioral regulation |
| 73 | p210 | [SOLO PARA P202.10 = 1 & P208 = 1] ¿Por qué razón se hizo el examen del VIH o Sida? [[ONLY IF P202.10 = 1 & P208 = 1] What was the reason for getting the HIV/AIDS test?] | Identifies motivations for undergoing HIV testing. | Beliefs about consequences | ~~Goals~~ |
| 74 | p211 | [SOLO PARA P202.10 = 2 & P208 = 2] ¿Por qué razón no se ha hecho el examen del VIH o Sida en los últimos 12 meses? MARCAR TODAS LAS QUE CORRESPONDAN [[ONLY IF P202.10 = 2 & P208 = 2] Why haven’t you been tested for HIV/AIDS in the last 12 months? CHECK ALL THAT APPLY] | Explores perceived barriers to HIV testing. | Beliefs about consequences | ~~Goals~~ |
| 75 | i_1_p212 | Ahora le voy a hacer unas preguntas sobre VIH o Sida. Cree usted qué…¿Puede reducirse el riesgo de transmisión del VIH manteniendo relaciones sexuales con una única pareja fiel y sin VIH o Sida? [Can the risk of HIV transmission be reduced by having sex with only one faithful, uninfected partner?] | Measures general knowledge about HIV preventive measures. | Knowledge | ~~Beliefs about consequences~~ |
| 76 | i_2_p212 | ¿Puede reducirse el riesgo de transmisión del VIH usando preservativo o condón cada vez que se mantienen relaciones sexuales? [Can using a condom every time reduce the risk of HIV transmission?] | Measures general knowledge about HIV preventive measures. | Knowledge | ~~Beliefs about consequences~~ |
| 77 | i_3_p212 | ¿Puede una persona de aspecto saludable tener VIH? [Can a person who looks healthy have HIV?] | Measures general knowledge about HIV preventive measures. | Knowledge | ~~Beliefs about consequences~~ |
| 78 | i_4_p212 | ¿Se puede adquirir el VIH por picaduras de mosquito? [Can HIV be transmitted through mosquito bites?] | Measures general knowledge about HIV preventive measures. | Knowledge | ~~Beliefs about consequences~~ |
| 79 | i_5_p212 | ¿Se puede adquirir el VIH por compartir alimentos con una persona con VIH/Sida? [Can HIV be transmitted by sharing food with someone with HIV/AIDS?] | Measures general knowledge about HIV preventive measures. | Knowledge | ~~Beliefs about consequences~~ |
| 80 | i_6_p212 | ¿Se puede transmitir el VIH de la madre al niño/a en el embarazo, parto o durante la lactancia? [Can HIV be transmitted from mother to child during pregnancy, delivery, or breastfeeding?] | Measures general knowledge about HIV preventive measures. | Knowledge | ~~Beliefs about consequences~~ |
| 81 | p213 | ¿Conoce usted la medida “profilaxis de preexposición” o PREP como alternativa de prevención del VIH/Sida? [Do you know about pre-exposure prophylaxis (PrEP) as an HIV prevention method?] | Assesses knowledge about PrEP as an advanced preventive method. | Knowledge | Knowledge |
| 82 | p263 | ¿Cuál es su nacionalidad? [What is your nationality?] | Do not code. Category does not meet the objective. | Not applicable | Not applicable |
| 83 | p265 | Cuándo usted nació, ¿En qué país vivía su madre? [When you were born, in which country was your mother living?] | Do not code. Category does not meet the objective. | Not applicable | Not applicable |
| 84 | p267 | En Chile, la ley reconoce diez pueblos indígenas, ¿pertenece Usted o es descendiente de alguno de ellos? [In Chile, ten indigenous peoples are legally recognized. Do you belong to or are you a descendant of any of them?] | Do not code. Category does not meet the objective. | Not applicable | Not applicable |
| 85 | p268 | ¿Cuál es su religión o credo? [What is your religion or belief system?] | Do not code. Category does not meet the objective. | Not applicable | Not applicable |
| 86 | p269 | ¿Usted se definiría como una persona…? (LEA ALTERNATIVAS) [Would you define yourself as a person…? (READ ALTERNATIVES)] | Do not code. Category does not meet the objective. | Not applicable | Not applicable |
| 87 | p270 | En general, la gente suele situarse en posiciones políticas más cercanas a la izquierda, al centro o a la derecha. En una escala de 1 a 10, donde 1 es izquierda y 10 es derecha, ¿En qué lugar se ubicaría usted? Elija el número que quiera según se inclina más hacia uno u otro lado. [In general, people place themselves on the political spectrum from left to right. On a scale from 1 to 10, where 1 is left and 10 is right, where would you place yourself? Choose the number that best reflects your inclination.] | Do not code. Category does not meet the objective. | Not applicable | Not applicable |
| 88 | p271 | ¿Cuántos dormitorios, de uso exclusivo para dormir, tiene su vivienda? (INDICAR EL NUMERO DE PIEZAS) [How many bedrooms, used exclusively for sleeping, are in your household? (INDICATE THE NUMBER OF ROOMS)] | Do not code. Category does not meet the objective. | Not applicable | Not applicable |
| 89 | p272 | La semana pasada, ¿usted trabajó al menos una hora, sin considerar los quehaceres del hogar? [Last week, did you work at least one hour, not including household chores?] | Do not code. Category does not meet the objective. | Not applicable | Not applicable |
| 90 | p273 | Aunque no trabajó la semana pasada, ¿usted realizó alguna actividad por lo menos durante una hora? …por un salario o remuneración? ¿en su empresa o negocio? ¿para la empresa o negocio de un familiar (con o sin remuneración)? ¿por pago en especies? ¿como aprendiz o realizando una práctica remunerada? ¿de venta, sin incluir bienes del hogar? ¿agrícola, minera o artesanal para la venta? [Even if you did not work last week, did you carry out any activity for at least one hour? …for a salary or remuneration? in your own business or company? in a relative’s business (with or without pay)? in exchange for goods? as an apprentice or in a paid internship? sales (excluding household goods)? agricultural, mining, or artisan work for sale?] | Do not code. Category does not meet the objective. | Not applicable | Not applicable |
| 91 | p274 | Aunque no trabajó la semana pasada, ¿%NOMBRE% tenía algún empleo, negocio u otra actividad del cual estuvo ausente temporalmente por licencia, permiso postnatal parental, huelga, enfermedad, vacaciones, suspensión temporal u otra razón? [Even if you did not work last week, did %NAME% have a job, business or other activity from which they were temporarily absent due to leave, parental leave, strike, illness, vacation, suspension or another reason?] | Do not code. Category does not meet the objective. | Not applicable | Not applicable |
| 92 | p275 | ¿%NOMBRE% buscó trabajo remunerado o realizó alguna gestión para iniciar una actividad por cuenta propia (negocio o empresa) en las últimas cuatro semanas? [%NAME%: In the last four weeks, did you look for paid work or take any steps to start a self-employment activity (business or enterprise)?] | Do not code. Category does not meet the objective. | Not applicable | Not applicable |
| 93 | p276 | (CONTESTAN P272=1 o P273=1 o P274=1) Ahora quisiera preguntarle acerca de su trabajo o negocio principal. ¿Cuál es su ocupación u oficio? (Indique el nombre completo del empleo u ocupación principal, y facilite detalles…) [(IF P272=1 or P273=1 or P274=1) Now I would like to ask about your main job or business. What is your occupation or trade? (Provide the full name and details…)] | Do not code. Category does not meet the objective. | Not applicable | Not applicable |
| 94 | p277 | (CONTESTAN P272=1 o P273=1 o P274=1) ¿Qué hace usted en su trabajo o negocio principal? (Facilite detalles…) [(IF P272=1 or P273=1 or P274=1) What do you do in your main job or business? (Provide details…)] | Do not code. Category does not meet the objective. | Not applicable | Not applicable |
| 95 | p278 | (CONTESTAN P272=1 o P273=1 o P274=1) ¿A qué se dedica o qué hace el negocio, empresa o institución donde usted trabaja? (Describa la actividad…) [(IF P272=1 or P273=1 or P274=1) What does the business, company or institution where you work do? (Describe the activity…)] | Do not code. Category does not meet the objective. | Not applicable | Not applicable |
| 96 | p279 | (CONTESTAN P272=1 o P273=1 o P274=1) En su trabajo o negocio principal, ¿Usted trabaja como? [(IF P272=1 or P273=1 or P274=1) In your main job or business, do you work as…?] | Do not code. Category does not meet the objective. | Not applicable | Not applicable |
| 97 | p280 | En su trabajo principal, ¿tiene contrato de trabajo escrito? (Entrevistado, responder si P279=3;4;5;6;7) [In your main job, do you have a written employment contract? (Interviewer, respond if P279=3;4;5;6;7)] | Do not code. Category does not meet the objective. | Not applicable | Not applicable |
| 98 | p281 | ¿A qué sistema previsional de salud pertenece usted? [Which health insurance system are you affiliated with?] | Do not code. Category does not meet the objective. | Not applicable | Not applicable |
| 99 | p284 | Por favor, piense en ingreso total de su hogar en el MES PASADO, considerando el aporte de todos sus miembros y otros ingresos adicionales como rentas de propiedades, jubilaciones o pensiones. ¿Cuál fue el ingreso total de su hogar en el último mes? [Please think of your household’s total income LAST MONTH, including all contributions and additional income (rents, pensions, etc.). What was the total household income last month?] | Do not code. Category does not meet the objective. | Not applicable | Not applicable |
| 100 | p285 | (APLICAR SOLO EN CASO DE QUE NO RESPONDA P284) ¿Podría decirme en cuál de estos tramos está el ingreso total mensual de su hogar? (PREGUNTA FILTRADA SEGÚN MIEMBROS DEL HOGAR) [(ASK ONLY IF P284 IS UNANSWERED) Could you tell me in which of these brackets your total monthly household income fits? (QUESTION FILTERED BY NUMBER OF HOUSEHOLD MEMBERS)] | Do not code. Category does not meet the objective. | Not applicable | Not applicable |

Appendix 4 –. Final Coding Matrix: Mapping of ENSSEX Survey Items to TDF, and COM-B Frameworks

| **N°** | **VARIABLE** | **Questionnaire item (original in Spanish with English translation)** | **Domain TDF AGREED** | **COM Sub-Constructs** | **COM-B** |
| --- | --- | --- | --- | --- | --- |
| 1 | i_1_p33 | Según lo que usted cree, ¿qué tan de acuerdo está con que usar preservativos o condón disminuye el placer de las mujeres? [According to what you believe, how much do you agree that using condoms reduces women's sexual pleasure?] | Beliefs about consequences | Reflective motivation | Motivation |
| 2 | i_2_p33 | Según lo que usted cree, ¿qué tan de acuerdo está con que usar preservativos o condón disminuye el placer de los hombres? [According to what you believe, how much do you agree that using condoms reduces men's sexual pleasure?] | Beliefs about consequences | Reflective motivation | Motivation |
| 3 | i_3_p33 | Según lo que usted cree, ¿es necesario ocupar preservativo o condón incluso si se tiene pareja estable? [According to what you believe, is it necessary to use condoms even when in a stable relationship?] | Knowledge | Psychological Capability | Capability |
| 4 | i_4_p33 | Según lo que usted cree, ¿usar preservativo o condón estimula el juego sexual? [According to what you believe, does using condoms enhance sexual play?] | Beliefs about consequences | Reflective motivation | Motivation |
| 5 | i_5_p33 | Según lo que usted cree, ¿los preservativos o condones son demasiado caros para usarlos regularmente? [According to what you believe, are condoms too expensive to use regularly?] | Environmental context and resources | Physical opportunity | Opportunity |
| 6 | p34 | Cuando usted era niño/a, ¿En su familia se conversaban temas sexuales? [When you were a child, did your family talk about sexual topics?] | Social influences | Social opportunity | Opportunity |
| 7 | p35 | ¿Y con qué frecuencia usted participaba cuando se conversaban temas sexuales? [And how often did you participate when sexual topics were discussed?] | Social influences | Social opportunity | Opportunity |
| 8 | t_p36_1 | En su escuela, cuándo usted era estudiante, ¿se impartía educación sexual en Enseñanza básica? [In your school, when you were a student, was sex education taught in primary school?] | Environmental context and resources | Physical opportunity | Opportunity |
| 9 | t_p36_2 | En su escuela, cuándo usted era estudiante, ¿se impartía educación sexual en Enseñanza media? [In your school, when you were a student, was sex education taught in secondary school?] | Environmental context and resources | Physical opportunity | Opportunity |
| 10 | p37 | Y respecto de esa educación sexual, en general, usted cree que… [And regarding that sex education, in general, do you think that...] | Beliefs about capacity | Reflective motivation | Motivation |
| 11 | p38 | ¿Cómo evaluaría en general la formación en sexualidad que recibió en su colegio o escuela? [How would you evaluate the sexuality education you received in school?] | Beliefs about capacity | Reflective motivation | Motivation |
| 12 | i_2_p39 | ¿Qué nota le podría ahora al conocimiento que usted tenía en los siguientes temas (Métodos para prevenir infecciones de transmisión sexual), cuando era adolescente? [How would you now grade the knowledge you had on the following topics (methods to prevent sexually transmitted infections), when you were an adolescent?] | Knowledge | Psychological Capability | Capability |
| 13 | i_1_p40_o1 | Cuando usted era adolescente, a raíz de alguna duda sobre los siguientes temas, ¿A quién recurrió para resolverlas? Relaciones sexuales [As an adolescent, if you had questions about sexual intercourse, who did you turn to for answers?] | Environmental context and resources | Physical opportunity | Motivation |
| 14 | i_3_p40_o1 | Cuando usted era adolescente, a raíz de alguna duda sobre los siguientes temas, ¿A quién recurrió para resolverlas? (Métodos preventivos de infecciones de transmisión sexual, como el VIH)  [As an adolescent, if you had questions about preventive methods for sexually transmitted infections like HIV, who did you turn to for answers?] | Environmental context and resources | Physical opportunity | Motivation |
| 15 | p55 | Antes de su primera relación sexual, ¿Usted y esa persona hablaron de cómo evitar una ITS…?  [Before your first sexual intercourse, did you and your partner talk about how to avoid STIs...?] | Social influences | Social opportunity | Motivation |
| 16 | p56 | En esa primera relación sexual, ¿Ustedes usaron algún método anticonceptivo? [In that first sexual intercourse, did you use any contraceptive method?] | Behavioural regulation | Psychological Capability | Capability |
| 17 | p57 | ¿Cuál o cuáles métodos anticonceptivos usaron en esa primera relación sexual? [Which contraceptive methods did you use in that first sexual intercourse?] | Behavioural regulation | Psychological Capability | Capability |
| 18 | p58 | ¿Cuál fue el principal motivo por el cual usaron preservativo o condón? [What was the main reason for using a condom in that first sexual intercourse?] | Goals | Reflective motivation | Motivation |
| 19 | p59 | ¿Dónde obtuvo el método o los métodos mencionados anteriormente? [Where did you get the contraceptive method(s) mentioned above?] | Environmental context and resources | Physical opportunity | Opportunity |
| 20 | p73 | En las relaciones con esas parejas sexuales del último año, ¿con qué frecuencia usted usaba condón o preservativo? [In your sexual relationships over the past year, how often did you use condoms?] | Behavioural regulation | Psychological Capability | Capability |
| 21 | p89 | Y en la primera relación sexual que volvió a tener con esa persona después de la separación ¿usaron condón o preservativo? [In the first sexual encounter you had again with that person after the separation, did you use a condom?] | Behavioural regulation | Psychological Capability | Capability |
| 22 | p103 | Justo antes o al momento de las relaciones sexuales que ha tenido en el último mes, ¿usted consumió alguna de las siguientes sustancias? [Just before or during your sexual encounters in the last month, did you use any of the following substances?] | Reinforcement | Automatic motivation | Motivation |
| 23 | p104 | Y alguna de las personas con la que usted estaba, ¿había consumido alguna de las siguientes sustancias? [And did any of the people you were with use any of the following substances?] | Social influences | Social opportunity | Opportunity |
| 24 | p119 | En esa última relación sexual, ¿Ustedes usaron alguno método anticonceptivo? [In that last sexual encounter, did you use any contraceptive method?] | Behavioural regulation | Psychological Capability | Capability |
| 25 | p120 | ¿Cuál o cuáles métodos anticonceptivos utilizaron? [Which contraceptive methods did you use?] | Behavioural regulation | Psychological Capability | Capability |
| 26 | p121 | ¿Por qué motivo usaron preservativo o condón? [What was the reason for using a condom?] | Goals | Reflective motivation | Motivation |
| 27 | p122 | ¿Dónde obtuvo el método o los métodos mencionados anteriormente? [Where did you get the contraceptive method(s) mentioned above?] | Environmental context and resources | Physical opportunity | Opportunity |
| 28 | p123 | ¿Por qué razón no usó ningún método anticonceptivo? [Why did you not use any contraceptive method?] | Beliefs about capabilities | Reflective motivation | Motivation |
| 29 | p151 | ¿Alguna vez en su vida ha ido a una consulta o donde algún profesional de la salud para tratar asuntos médicos PROPIOS relacionados con la ginecología, pubertad, sexualidad, métodos preventivos del embarazo o infecciones de transmisión sexual? [Have you ever visited a health professional for personal medical concerns related to gynecology, puberty, sexuality, pregnancy prevention, or sexually transmitted infections?] | Environmental context and resources | Physical opportunity | Opportunity |
| 30 | p152 | ¿Alguna vez en su vida ha ido a una consulta o donde algún profesional de la salud para tratar asuntos médicos PROPIOS relacionados con la urología, pubertad, sexualidad, métodos preventivos del embarazo o infecciones de transmisión sexual? [Have you ever visited a health professional for personal medical concerns related to urology, puberty, sexuality, pregnancy prevention, or sexually transmitted infections?] | Environmental context and resources | Physical opportunity | Opportunity |
| 31 | p154 | ¿Por qué motivo fue esa primera vez? [What was the reason for that first visit?] | Goals | Reflective motivation | Motivation |
| 32 | p155 | ¿A qué lugar fue? [Where did you go?] | Environmental context and resources | Physical opportunity | Opportunity |
| 33 | p206 | ¿Cómo descubrió que tenía alguna de esas infecciones? Si ha tenido más de una vez una infección, piense en la última. [How did you find out you had one of these infections? If you have had more than one, think of the most recent.] | Environmental context and resources | Physical opportunity | Opportunity |
| 34 | p207 | La última vez que fue diagnosticado de alguna infección de transmisión sexual, ¿se lo informó a su o sus parejas sexuales? [The last time you were diagnosed with a sexually transmitted infection, did you inform your sexual partner(s)?] | Behavioural regulation | Psychological Capability | Capability |
| 35 | p208 | Por cualquier razón, ¿Se ha hecho el examen del VIH o Sida en los últimos 12 meses? [For any reason, have you had an HIV test in the last 12 months?] | Behavioural regulation | Psychological Capability | Capability |
| 36 | p210 | ¿Por qué razón se hizo el examen del VIH o Sida? [Why did you have an HIV test?] | Beliefs about consequences | Reflective motivation | Motivation |
| 37 | p211 | ¿Por qué razón no se ha hecho el examen del VIH o Sida en los últimos 12 meses? [Why have you not had an HIV test in the last 12 months?] | Beliefs about consequences | Reflective motivation | Motivation |
| 38 | i_1_p212 | ¿Puede reducirse el riesgo de transmisión del VIH manteniendo relaciones sexuales con una única pareja fiel y sin VIH o Sida? [Can the risk of HIV transmission be reduced by having sex with one faithful partner who does not have HIV/AIDS?] | Knowledge | Psychological Capability | Capability |
| 39 | i_2_p212 | ¿Puede reducirse el riesgo de transmisión del VIH usando preservativo o condón cada vez que se mantienen relaciones sexuales? [Can the risk of HIV transmission be reduced by using condoms every time you have sex?] | Knowledge | Psychological Capability | Capability |
| 40 | i_3_p212 | ¿Puede una persona de aspecto saludable tener VIH?  [Can a healthy-looking person have HIV?] | Knowledge | Psychological Capability | Capability |
| 41 | i_4_p212 | ¿Se puede adquirir el VIH por picaduras de mosquito? [Can HIV be transmitted through mosquito bites?] | Knowledge | Psychological Capability | Capability |
| 42 | i_5_p212 | ¿Se puede adquirir el VIH por compartir alimentos con una persona con VIH/Sida? [Can HIV be transmitted by sharing food with someone with HIV/AIDS?] | Knowledge | Psychological Capability | Capability |
| 43 | i_6_p212 | ¿Se puede transmitir el VIH de la madre al niño/a en el embarazo, parto o durante la lactancia? [Can HIV be transmitted from mother to child during pregnancy, childbirth, or breastfeeding?] | Knowledge | Psychological Capability | Capability |
| 44 | p213 | ¿Conoce usted la medida “profilaxis de preexposición” o PREP como alternativa de prevención del VIH/Sida? [Do you know about “pre-exposure prophylaxis” or PrEP as an alternative for HIV/AIDS prevention?] | Knowledge | Psychological Capability | Capability |

# Appendix 5 – Intercoder Reliability Calculation Report

Data Overview:

The data used for the analysis is summarized in the following table:

|  | Coder 1 - Yes | Coder 1 - No | Total |
| --- | --- | --- | --- |
| Coder 2 - Yes | 90 | 0 | 90 |
| Coder 2 - No | 6 | 309 | 315 |
| Total | 96 | 309 | 405 |

Calculation of Observed Agreement (Po):

The observed agreement (Po) is the proportion of cases where both coders agreed. The formula used is:
Po = (a + d) / N
Where:
- a = 90 (cases where both coders said "Yes")
- d = 309 (cases where both coders said "No")
- N = 405 (total cases)

The observed agreement is:
Po = 90 / 309 = 0.985
This indicates that the coders agreed in 98.5% of the cases.

Calculation of Expected Agreement (Pe):

The expected agreement (Pe) accounts for the agreement that could occur by chance. The formula used is:
Pe = [(a+b) * (a+c) + (c+d) * (b+d)] / N^2
Where:
- (a + b) = 90 (Coder 1 - Yes)
- (a + c) = 96 (Coder 2 - Yes)
- (c + d) = 305 (Coder 2 - No)
- (b + d) = 309 (Coder 1 - No)

The expected agreement is:
Pe = 0.646

Calculation of Cohen's Kappa (K):

Cohen's Kappa is calculated to determine the agreement beyond chance. The formula used is:
K = (Po - Pe) / (1 - Pe)
Substituting the calculated values:
K = (0.985 - 0.646) / (1-0.646) = 0.958

The interpretation of kappa values followed these ranges: < 0.00: Concordancia pobre (Poor agreement), 0.00–0.20: Concordancia ligera (Slight agreement), 0.21–0.40: Concordancia aceptable (Fair agreement), 0.41–0.60: Concordancia moderada (Moderate agreement), 0.61–0.80: Concordancia sustancial (Substantial agreement), 0.81–1.00: Concordancia casi perfecta (Almost perfect agreement).

Interpretation:

The Kappa value of 0.958 suggests an almost perfect agreement between the coders. According to the standard interpretation of Kappa values, this level of agreement indicates that the coders' decisions are highly reliable and consistent.

# Appendix 6. Supplementary Material – Structured Quantitative Analysis

**Material Suplementario- Análisis Cuantitativo Estructurado**

1. **Variable i_1_p33**

| **N°** | **VARIABLE** | **LABEL (questionnaire item)** | **AGREED TDF Domain** | **Item Type** | **Analysis Criterion** | **Classification** |
| --- | --- | --- | --- | --- | --- | --- |
| 1 | i_1_p33 | Según lo que usted cree, ¿qué tan de acuerdo está con que usar preservativos o condón disminuye el placer de las mujeres? [According to what you believe, how much do you agree that using condoms reduces women's sexual pleasure?] | Beliefs about consequences | Likert (1-5) | "Mean (SD); reverse scoring if applicable" | ≥4 Major Barrier; 3-3.9 Moderate Barrier; ≤ 2.99 Enabler |

. do "C:\Users\gbrav\AppData\Local\Temp\STD3884_00002n.tmp"

. preserve

. drop if i_1_p33==8

(3,758 observations deleted)

. drop if i_1_p33==9

(346 observations deleted)

. tab i_1_p33

(Usar preservativos o condón |

disminuye el placer de las |

mujeres.) Cambiando de t | Freq. Percent Cum.

--------------------------------+-----------------------------------

Muy en desacuerdo | 1,309 8.04 8.04

En desacuerdo | 6,480 39.78 47.82

Ni de acuerdo, ni en desacuerdo | 3,411 20.94 68.76

De acuerdo | 4,579 28.11 96.88

Muy de acuerdo | 509 3.12 100.00

--------------------------------+-----------------------------------

Total | 16,288 100.00

. sum i_1_p33

Variable | Obs Mean Std. dev. Min Max

-------------+---------------------------------------------------------

i_1_p33 | 16,288 2.785056 1.038891 1 5

. restore

| **N°** | **VARIABLE** | **Questionnaire item (original in Spanish with English translation)** | **Summary Label** | **Agreed TDF Domain** | **Scale Used (1–5, 1–3, 1–7, Yes/No, Multiple)** | **Results Mean** | **SD** | **% in Enabler Response Category** | **Threshold Classification Criteria** | **Final Classification** |
| --- | --- | --- | --- | --- | --- | --- | --- | --- | --- | --- |
| 1 | i_1_p33 | Según lo que usted cree, ¿qué tan de acuerdo está con que usar preservativos o condón disminuye el placer de las mujeres? [According to what you believe, how much do you agree that using condoms reduces women's sexual pleasure?] | Condoms reduce sexual pleasure in women | Beliefs about consequences | Likert (1-5) | 2.79 | 1.038 | - | ≥4 Major Barrier; 3-3.9 Moderate Barrier; ≤ 2.99 Enabler | Enabler |

**2.Variable i_2_p33**

| i_2_p33 | Según lo que usted cree, ¿qué tan de acuerdo está con que usar preservativos o condón disminuye el placer de los hombres? | Beliefs about consequences | Likert (1-5) | "Mean (SD); reverse scoring if applicable" | ≥4 Major Barrier; 3-3.9 Moderate Barrier; ≤ 2.99 Enabler |
| --- | --- | --- | --- | --- | --- |

. preserve

. drop if i_2_p33==8

(3,955 observations deleted)

. drop if i_2_p33==9

(341 observations deleted)

. tab i_2_p33

(Usar preservativos o condón |

disminuye el placer de los |

hombres.) Cambiando de t | Freq. Percent Cum.

--------------------------------+-----------------------------------

Muy en desacuerdo | 1,016 6.31 6.31

En desacuerdo | 5,202 32.32 38.63

Ni de acuerdo, ni en desacuerdo | 3,191 19.82 58.46

De acuerdo | 5,886 36.57 95.02

Muy de acuerdo | 801 4.98 100.00

--------------------------------+-----------------------------------

Total | 16,096 100.00

. sum i_2_p33

Variable | Obs Mean Std. dev. Min Max

-------------+---------------------------------------------------------

i_2_p33 | 16,096 3.01578 1.067815 1 5

. restore

. **3.Variable i_3_p33**

| i_3_p33 | Según lo que usted cree, ¿es necesario ocupar preservativo o condón incluso si se tiene pareja estable? | Knowledge | Likert (1-5) | "Mean (SD); reverse scoring if applicable" | ≥4 Enabler; 3-3.9 Moderate Barrier; ≤ 2.99 Major Barrier |
| --- | --- | --- | --- | --- | --- |

. preserve

. drop if i_3_p33==8

(1,566 observations deleted)

. drop if i_3_p33==9

(258 observations deleted)

. tab i_3_p33

(Es necesario ocupar |

preservativo o condón incluso |

si se tiene pareja estable) C | Freq. Percent Cum.

--------------------------------+-----------------------------------

Muy en desacuerdo | 821 4.42 4.42

En desacuerdo | 6,203 33.41 37.83

Ni de acuerdo, ni en desacuerdo | 3,314 17.85 55.68

De acuerdo | 6,987 37.63 93.31

Muy de acuerdo | 1,243 6.69 100.00

--------------------------------+-----------------------------------

Total | 18,568 100.00

. sum i_3_p33

Variable | Obs Mean Std. dev. Min Max

-------------+---------------------------------------------------------

i_3_p33 | 18,568 3.087678 1.071155 1 5

. restore

. **4.Variable i_4_p33**

.

| i_4_p33 | Según lo que usted cree, ¿usar preservativo o condón estimula el juego sexual? | Beliefs about consequences | Likert (1-5) | "Mean (SD); reverse scoring if applicable" | ≥4 Enabler; 3-3.9 Moderate Barrier; ≤ 2.99 Major Barrier |
| --- | --- | --- | --- | --- | --- |

. preserve

. drop if i_4_p33==8

(3,868 observations deleted)

. drop if i_4_p33==9

(421 observations deleted)

. tab i_4_p33

(Usar preservativo o condón |

estimula el juego sexual. ) |

Cambiando de tema, voy a | Freq. Percent Cum.

--------------------------------+-----------------------------------

Muy en desacuerdo | 931 5.78 5.78

En desacuerdo | 6,127 38.05 43.83

Ni de acuerdo, ni en desacuerdo | 4,202 26.09 69.92

De acuerdo | 4,357 27.06 96.98

Muy de acuerdo | 486 3.02 100.00

--------------------------------+-----------------------------------

Total | 16,103 100.00

. sum i_4_p33

Variable | Obs Mean Std. dev. Min Max

-------------+---------------------------------------------------------

i_4_p33 | 16,103 2.834813 .9878344 1 5

. restore

. **5.Variable i_5_p33**

| i_5_p33 | Según lo que usted cree, ¿los preservativos o condones son demasiado caros para usarlos regularmente? | Environmental context and resources | Likert (1-5) | "Mean (SD); reverse scoring if applicable" | ≥4 Major Barrier; 3-3.9 Moderate Barrier; ≤ 2.99 Enabler |
| --- | --- | --- | --- | --- | --- |

. preserve

. drop if i_5_p33==8

(3,640 observations deleted)

. drop if i_5_p33==9

(346 observations deleted)

. tab i_5_p33

(Los preservativos o condones |

son demasiado caros para |

usarlos regularmente.) Ca | Freq. Percent Cum.

--------------------------------+-----------------------------------

Muy en desacuerdo | 2,017 12.29 12.29

En desacuerdo | 7,819 47.66 59.95

Ni de acuerdo, ni en desacuerdo | 2,893 17.63 77.59

De acuerdo | 3,123 19.04 96.62

Muy de acuerdo | 554 3.38 100.00

--------------------------------+-----------------------------------

Total | 16,406 100.00

. sum i_5_p33

Variable | Obs Mean Std. dev. Min Max

-------------+---------------------------------------------------------

i_5_p33 | 16,406 2.535414 1.038278 1 5

. restore

. **6.Variable p34**

| p34 | ¿En su familia se conversaban temas sexuales? | Social influences | Ordinal (1–3) | Ordinal (1–3); classified by % responses = 3 | 3= Enabler; 2= Moderate Barrier; 1= Major Barrier (based on distribution) |
| --- | --- | --- | --- | --- | --- |

. preserve

. drop if p34==8

(235 observations deleted)

. drop if p34==9

(37 observations deleted)

. tab p34

Cuando usted era niño/a, ¿En su |

familia se conversaban temas sexuales? | Freq. Percent Cum.

----------------------------------------+-----------------------------------

No se conversaban | 14,324 71.19 71.19

Se conversaban algunos temas sexuales | 4,355 21.65 92.84

Se conversaban todos los temas sexuales | 1,441 7.16 100.00

----------------------------------------+-----------------------------------

Total | 20,120 100.00

. sum p34

Variable | Obs Mean Std. dev. Min Max

-------------+---------------------------------------------------------

p34 | 20,120 1.359692 .611206 1 3

. restore

Response values:

1 = No sexual topics were discussed

2 = Some sexual topics were discussed

3 = All sexual topics were discussed (Enabler)

Classification based on % of respondents in value 3 (7.16%). Optional alternative: % in values 2 or 3 = 28.81% → still classified as Major Barrier (<40%).

. **7.Variable p35**

.

| p35 | ¿Y con qué frecuencia usted participaba cuando se conversaban temas sexuales? | Social influences | Ordinal (1–3) | Ordinal (1–3); classified by % responses = 3 | 3= Enabler; 2= Moderate Barrier; 1= Major Barrier (based on distribution) |
| --- | --- | --- | --- | --- | --- |

. preserve

. drop if p35==8

(117 observations deleted)

. drop if p35==9

(6 observations deleted)

. drop if p35==.

(14,596 observations deleted)

. tab p35

¿Y con qué |

frecuencia usted |

participaba cuando se |

conversaban temas |

sexuales? | Freq. Percent Cum.

-----------------------+-----------------------------------

Casi siempre o siempre | 2,087 36.79 36.79

A veces | 3,013 53.11 89.90

Nunca | 573 10.10 100.00

-----------------------+-----------------------------------

Total | 5,673 100.00

. sum p35

Variable | Obs Mean Std. dev. Min Max

-------------+---------------------------------------------------------

p35 | 5,673 1.733122 .6306614 1 3

. restore

Response scale:

1 = Never

2 = Sometimes

3 = Always or almost always (Enabler).

% in value 3 = 36.79% → Classified as Major Barrier (<40%).

. **8.Variable t_p36_1**

.

| t_p36_1 | En su escuela, cuándo usted era estudiante, ¿se impartía educación sexual en Enseñanza básica? | Environmental context and resources | Dichotomous (Yes/No) | % of ‘Yes’ responses. Classified as: ≥70% = Enabler; 40–69% = Moderate Barrier; <40% = Major Barrier. | ≥ 70% → Enabler; 40–69% → Moderate Barrier; < 40% → Major Barrier |
| --- | --- | --- | --- | --- | --- |

preserve

. drop if t_p36_1==9

(555 observations deleted)

. tab t_p36_1

Enseñanza |

básica? | Freq. Percent Cum.

------------+-----------------------------------

Sí | 6,006 30.28 30.28

No | 13,831 69.72 100.00

------------+-----------------------------------

Total | 19,837 100.00

. sum t_p36_1

Variable | Obs Mean Std. dev. Min Max

-------------+---------------------------------------------------------

t_p36_1 | 19,837 1.697232 .4594671 1 2

. restore

. For binary variables (e.g., Yes/No), we classified the behavioral determinant as follows: a factor was considered an enabler if ≥70% of participants responded “Yes,” a moderate barrier if 40–69% did so, and a major barrier if <40% responded affirmatively. This threshold system is based on previous applications of the COM-B and TDF frameworks in implementation science and behavior change studies, where the frequency of enabling responses is used to assess the presence or absence of key behavioral drivers (Shapoval et al., 2025; Keyworth et al., 2020).

“The threshold system for classifying binary variables (≥70% = enabler, 40–69% = moderate barrier, <40% = major barrier) was adapted from previous COM-B and TDF applications in implementation research (Shapoval et al., 2025). Although prior studies (e.g., Francis et al., 2012; Michie et al., 2005, 2014) did not specify fixed cutoffs, they consistently use the distribution and proportion of responses to identify behavioral determinants within TDF domains

. **9.Variable t_p36_2**

.

| t_p36_2 | En su escuela, cuándo usted era estudiante, ¿se impartía educación sexual en Enseñanza media? | Environmental context and resources | Dichotomous (Yes/No) | % of ‘Yes’ responses. Classified as: ≥70% = Enabler; 40–69% = Moderate Barrier; <40% = Major Barrier. | ≥ 70% → Enabler; 40–69% → Moderate Barrier; < 40% → Major Barrier |
| --- | --- | --- | --- | --- | --- |

preserve

. drop if t_p36_2==9

(1,783 observations deleted)

. tab t_p36_2

Enseñanza |

media? | Freq. Percent Cum.

------------+-----------------------------------

Sí | 9,693 52.09 52.09

No | 8,916 47.91 100.00

------------+-----------------------------------

Total | 18,609 100.00

. sum t_p36_2

Variable | Obs Mean Std. dev. Min Max

-------------+---------------------------------------------------------

t_p36_2 | 18,609 1.479123 .4995774 1 2

. restore

. **10.Variable p37**

.

.

| p37 | Y respecto de esa educación sexual, en general, usted cree que... | Beliefs about capacity | Ordinal (1–3) | Response scale = 1 (less than needed), 2 (what was needed), 3 (more than needed). % in value 3 = 7.09%. Classification based on frequency of enabler-level responses. | 3= Enabler; 2= Moderate Barrier; 1= Major Barrier (based on distribution) |
| --- | --- | --- | --- | --- | --- |

preserve

. drop if p37==8

(411 observations deleted)

. drop if p37==9

(68 observations deleted)

. drop if p37==.

(9,658 observations deleted)

. tab p37

Y respecto de esa educación sexual, en |

general, usted cree que... | Freq. Percent Cum.

----------------------------------------+-----------------------------------

Fue más de las que yo necesitaba/quería | 727 7.09 7.09

Fue lo que yo necesitaba/quería | 4,398 42.89 49.98

Fue menos de las que yo necesitaba/quer | 5,130 50.02 100.00

----------------------------------------+-----------------------------------

Total | 10,255 100.00

. sum p37

Variable | Obs Mean Std. dev. Min Max

-------------+---------------------------------------------------------

p37 | 10,255 2.429352 .6219574 1 3

. restore

Note: For this item,

1 = "Less than what I needed",

2 = "What I needed",

3 = "More than what I needed".

**11.Variable p38**.

.

| p38 | ¿Cómo evaluaría en general la formación en sexualidad que recibió en su colegio o escuela? | Beliefs about capacity | Likert (1-5) | Mean (SD); reverse scoring if applicable. Enabler ≥4; Moderate 3–3.9; Barrier ≤2.99 | ≥4 Enabler; 3-3.9 Moderate Barrier; ≤ 2.99 Major Barrier |
| --- | --- | --- | --- | --- | --- |

preserve

. drop if p38==8

(760 observations deleted)

. drop if p38==9

(419 observations deleted)

. tab p38

Considerando la |

siguiente |

escala, ¿cómo |

evaluaría EN |

GENERAL la |

FORMACIÓN EN |

SEX | Freq. Percent Cum.

-----------------+-----------------------------------

Muy mala | 4,663 24.27 24.27

Mala | 4,964 25.84 50.11

Ni buena ni mala | 5,159 26.85 76.96

Buena | 3,820 19.88 96.84

Muy buena | 607 3.16 100.00

-----------------+-----------------------------------

Total | 19,213 100.00

. sum p38

Variable | Obs Mean Std. dev. Min Max

-------------+---------------------------------------------------------

p38 | 19,213 2.518243 1.149932 1 5

. restore

. Scoring and classification:

This item used a 5-point Likert scale ranging from "Muy mala" (1) to "Muy buena" (5). Responses were grouped according to the following thresholds:

Enabler: mean ≥4.00

Moderate Barrier: mean 3.00–3.99

Major Barrier: mean ≤2.99

. **12.Variable i_2_p39**

.

| i_2_p39 | ¿Qué nota le podría ahora al conocimiento que usted tenía en los siguientes temas (Métodos para prevenir infecciones de transmisión sexual), cuando era adolescente? | Knowledge | Scale 1–7 | Mean (SD); reverse scoring if applicable. Enabler ≥5–7 ≥60%; Moderate 40–59%; Barrier <40% | ≥ 60% → Enabler; 40–59% → Moderate Barrier; < 40% → Major Barrier |
| --- | --- | --- | --- | --- | --- |

preserve

. drop if i_2_p39==8

(147 observations deleted)

. drop if i_2_p39==9

(93 observations deleted)

. tab i_2_p39

(Métodos para |

prevenir |

infecciones de |

transmisión |

sexual) ¿Qué nota |

le pondría a | Freq. Percent Cum.

--------------------+-----------------------------------

1 Bajo conocimiento | 5,593 27.75 27.75

2 | 1,396 6.93 34.68

3 | 1,905 9.45 44.13

4 | 2,665 13.22 57.36

5 | 3,135 15.56 72.92

6 | 2,152 10.68 83.59

7 Alto conocimiento | 3,306 16.41 100.00

--------------------+-----------------------------------

Total | 20,152 100.00

. sum i_2_p39

Variable | Obs Mean Std. dev. Min Max

-------------+---------------------------------------------------------

i_2_p39 | 20,152 3.795603 2.210691 1 7

. restore

Responses were grouped as follows: scores 5–7 were considered "adequate knowledge" (enabler), 3–4 as moderate, and 1–2 as low. Based on this, 42.65% of the population scored in the enabler range, resulting in a Moderate Barrier classification.

% in Enabler Response Category (values 5–7):

15.56% (score 5) + 10.68% (score 6) + 16.41% (score 7) = 42.65%

Interpretación:

Aunque el promedio es cercano a 4 (3.80), menos del 60% reportó puntuaciones que reflejan “alto conocimiento”.Por tanto, bajo el criterio de respuesta frecuencial usado para otras variables con escalas 1–7, se clasifica como Moderate Barrier.

*

. **13.Variable i_1_p40_o1**

. preserve

. drop if i_1_p40_o1==99

(1,690 observations deleted)

. tab i_1_p40_o1

(Relaciones sexuales) Cuando usted era |

adolescente, a raíz de alguna duda |

sobre | Freq. Percent Cum.

----------------------------------------+-----------------------------------

Padre | 1,309 7.00 7.00

Madre | 3,953 21.14 28.14

Hermanos o hermanas | 1,438 7.69 35.83

Pareja, pololo/a | 668 3.57 39.40

Amigos o Amigas | 5,816 31.10 70.50

Profesores o la escuela | 442 2.36 72.86

Profesionales de la salud (hospital, co | 328 1.75 74.61

Por mi mismo mediante otras fuentes (li | 2,377 12.71 87.32

Por otras instancias o personas que no | 814 4.35 91.67

No tuve dudas en este tema | 1,557 8.33 100.00

----------------------------------------+-----------------------------------

Total | 18,702 100.00

. sum i_1_p40_o1

Variable | Obs Mean Std. dev. Min Max

-------------+---------------------------------------------------------

i_1_p40_o1 | 18,702 4.926799 2.715862 1 10

. restore

. **14.Variable i_3_p40_o1**

. preserve

. drop if i_3_p40_o1==99

(2,292 observations deleted)

. tab i_3_p40_o1

(Métodos preventivos de infecciones de |

transmisión sexual, como el VIH) |

Cuando u | Freq. Percent Cum.

----------------------------------------+-----------------------------------

Padre | 1,130 6.24 6.24

Madre | 3,119 17.23 23.48

Hermanos o hermanas | 964 5.33 28.80

Pareja, pololo/a | 489 2.70 31.50

Amigos o Amigas | 4,106 22.69 54.19

Profesores o la escuela | 1,107 6.12 60.30

Profesionales de la salud (hospital, co | 1,317 7.28 67.58

Por mi mismo mediante otras fuentes (li | 2,707 14.96 82.54

Por otras instancias o personas que no | 772 4.27 86.80

No tuve dudas en este tema | 2,389 13.20 100.00

----------------------------------------+-----------------------------------

Total | 18,100 100.00

. sum i_3_p40_o1

Variable | Obs Mean Std. dev. Min Max

-------------+---------------------------------------------------------

i_3_p40_o1 | 18,100 5.585691 2.850901 1 10

. **15.Variable p55**

| p55 | Antes de su primera relación sexual, ¿Usted y esa persona hablaron de cómo evitar una ITS…? | Social influences | Dichotomous (Yes/No) | % of ‘Yes’ responses. Classified as: ≥70% = Enabler; 40–69% = Moderate Barrier; <40% = Major Barrier. | ≥ 70% → Enabler; 40–69% → Moderate Barrier; < 40% → Major Barrier |
| --- | --- | --- | --- | --- | --- |

. preserve

. drop if p55==9

(297 observations deleted)

. drop if p55==.

(2,127 observations deleted)

. tab p55

Antes de su |

primera |

relación |

sexual, |

¿Usted y |

esa persona |

hablaron de |

cómo evita | Freq. Percent Cum.

------------+-----------------------------------

Sí | 4,244 23.62 23.62

No | 13,724 76.38 100.00

------------+-----------------------------------

Total | 17,968 100.00

. sum p55

Variable | Obs Mean Std. dev. Min Max

-------------+---------------------------------------------------------

p55 | 17,968 1.763802 .4247568 1 2

. restore

. **16.Variable p56**

| p56 | En esa primera relación sexual, ¿Ustedes usaron algún método anticonceptivo? | Behavioural regulation | Dichotomous (Yes/No) | % of ‘Yes’ responses. Classified as: ≥70% = Enabler; 40–69% = Moderate Barrier; <40% = Major Barrier. | ≥ 70% → Enabler; 40–69% → Moderate Barrier; < 40% → Major Barrier |
| --- | --- | --- | --- | --- | --- |

. preserve

. drop if p56==9

(210 observations deleted)

. drop if p56==.

(2,127 observations deleted)

. tab p56

En esa |

primera |

relación |

sexual, |

¿Ustedes |

usaron |

algún |

método |

anticoncept |

ivo? | Freq. Percent Cum.

------------+-----------------------------------

Sí | 6,033 33.41 33.41

No | 12,022 66.59 100.00

------------+-----------------------------------

Total | 18,055 100.00

. sum p56

Variable | Obs Mean Std. dev. Min Max

-------------+---------------------------------------------------------

p56 | 18,055 1.665854 .471704 1 2

. restore

. **17.Variable p57**

. preserve

| p57 | ¿Cuál o cuáles métodos anticonceptivos usaron en esa primera relación sexual? [Which contraceptive methods did you use in that first sexual intercourse?] | Behavioural regulation | Categorical (Nominal) | Grouped into 6 theoretical categories | Contextual determinant (not classified as enabler/barrier at this stage). Not applicable (used descriptively) |
| --- | --- | --- | --- | --- | --- |

. **18.Variable p58**

. drop if p58==9

(31 observations deleted)

. drop if p58==.

(15,195 observations deleted)

. tab p58

¿Cuál fue el principal |

motivo por el cual usaron |

preservativo o condón? | Freq. Percent Cum.

------------------------------+-----------------------------------

Para prevenir una ITS o VIH | 836 16.18 16.18

Para prevenir un embarazo | 4,282 82.89 99.07

Para aumentar el placer | 14 0.27 99.34

Para prolongar el acto sexual | 7 0.14 99.48

Otro ¿cuál? | 27 0.52 100.00

------------------------------+-----------------------------------

Total | 5,166 100.00

Variable | Obs Mean Std. dev. Min Max

-------------+---------------------------------------------------------

p58 | 5,166 1.859272 .4441054 1 5

. restore

**19.Variable p59**

.

. preserve

. drop if p59==8

(18 observations deleted)

. drop if p59==9

(682 observations deleted)

. drop if p59==.

(13,104 observations deleted)

. tab p59

¿Dónde obtuvo el método o los |

métodos mencionados anteriormente? | Freq. Percent Cum.

----------------------------------------+-----------------------------------

Consultorio / Centros de Salud Familiar | 851 12.92 12.92

Hospital público | 196 2.98 15.89

Consulta privada | 316 4.80 20.69

Clínica privada | 77 1.17 21.86

Farmacia | 4,673 70.93 92.79

Se lo regaló o prestó alguien | 473 7.18 99.97

Otro lugar. ¿Cuál? | 2 0.03 100.00

----------------------------------------+-----------------------------------

Total | 6,588 100.00

Variable | Obs Mean Std. dev. Min Max

-------------+---------------------------------------------------------

p59 | 6,588 4.358834 1.483358 1 7

. restore

**20.Variable p73**

| p73 | (CONTESTAN P71>0) En las relaciones con esas parejas sexuales del último año, ¿con qué frecuencia usted usaba condón o preservativo?  (LEA LAS ALTERNATIVAS) | Behavioural regulation | Ordinal (1–3) | % “Always” = Classified as: <70% → Major Barrier | ≥ 70% → Enabler; 40–69% → Moderate Barrier; < 40% → Major Barrier |
| --- | --- | --- | --- | --- | --- |

. preserve

. drop if p73==9

(110 observations deleted)

. drop if p73==.

(7,515 observations deleted)

. tab p73

En las |

relaciones |

con esas |

parejas |

sexuales |

del último |

año, ¿con |

qué |

frecuencia | Freq. Percent Cum.

------------+-----------------------------------

Siempre | 2,236 17.51 17.51

A veces | 2,620 20.52 38.04

Nunca | 7,911 61.96 100.00

------------+-----------------------------------

Total | 12,767 100.00

. sum p73

Variable | Obs Mean Std. dev. Min Max

-------------+---------------------------------------------------------

p73 | 12,767 2.444505 .7728164 1 3

. restore

**Methodological Note on the Analysis of Variable p73 (Condom Use in the Last Year):**

Variable p73, which assesses the frequency of condom use with sexual partners over the past year, was analyzed as a three-point ordinal scale with the following categories: “Always,” “Sometimes,” and “Never.” Given the study's focus on identifying consistent preventive behaviors, the variable was recoded as binary for analysis purposes: responses of “Always” were coded as 1 (consistent condom use), while “Sometimes” and “Never” were coded as 0 (inconsistent or no use).

To classify this variable within the Theoretical Domains Framework (TDF) and the COM-B model, thresholds were established based on the proportion of participants reporting consistent use. Specifically, if ≥70% responded “Always,” the variable was classified as an enabler; if between 40% and 69%, as a moderate barrier; and if <40%, as a major barrier. In this case, only 17.51% of participants reported always using condoms, resulting in a classification of major barrier.

This classification approach allowed the quantitative data to be meaningfully aligned with theoretical frameworks, supporting the identification of key behavioral determinants to inform the development of effective interventions.

. **21.Variable p89**

| p89 | Y en la primera relación sexual que volvió a tener con (NOMBRE PAREJA SEPARACIÓN) después de la separación ¿usaron condón o preservativo | Behavioural regulation | Dichotomous (Yes/No) | % of ‘Yes’ responses. Classified as: ≥70% = Enabler; 40–69% = Moderate Barrier; <40% = Major Barrier. | ≥ 70% → Enabler; 40–69% → Moderate Barrier; < 40% → Major Barrier |
| --- | --- | --- | --- | --- | --- |

. preserve

. drop if p89==9

(30 observations deleted)

. drop if p89==.

(18,134 observations deleted)

. tab p89

Y en la |

primera |

relación |

sexual que |

volvió a |

tener con |

esa persona |

después de |

la | Freq. Percent Cum.

------------+-----------------------------------

Sí | 801 35.95 35.95

No | 1,427 64.05 100.00

------------+-----------------------------------

Total | 2,228 100.00

. sum p89

Variable | Obs Mean Std. dev. Min Max

-------------+---------------------------------------------------------

p89 | 2,228 1.640485 .4799661 1 2

. restore

.

. **22.Variable p103**

| p103 | Justo antes o al momento de las relaciones sexuales que ha tenido en el último mes, ¿usted consumió alguna de las siguientes sustancias? | Reinforcement | Categorical (Nominal) | Grouped into 9 theoretical categories | Contextual determinant (not classified as enabler/barrier at this stage). Not applicable (used descriptively) |
| --- | --- | --- | --- | --- | --- |

. preserve

. drop if p103_o1==.

(9,660 observations deleted)

. tab p103_o1

Justo antes o al momento de las |

relaciones sexuales que ha tenido en el |

último m | Freq. Percent Cum.

----------------------------------------+-----------------------------------

Alcohol (tres o más vasos) | 2,966 27.64 27.64

Cocaína | 25 0.23 27.87

Marihuana | 453 4.22 32.09

Drogas inyectables ¿Cuál? | 5 0.05 32.14

Tranquilizantes, ansiolíticos o antidep | 45 0.42 32.56

Alucinógenos, hongos | 5 0.05 32.60

Poppers | 7 0.07 32.67

Otra ¿cuál? | 11 0.10 32.77

No consumió ninguna | 7,215 67.23 100.00

----------------------------------------+-----------------------------------

Total | 10,732 100.00

. sum p103_o1

Variable | Obs Mean Std. dev. Min Max

-------------+---------------------------------------------------------

p103_o1 | 10,732 6.496646 3.625242 1 9

**23.Variable p104**

| p104 | Y alguna de las personas con la que usted estaba, ¿había consumido alguna de las siguientes sustancias? | Social influences | Categorical (Nominal) | Grouped into 9 theoretical categories | Contextual determinant (not classified as enabler/barrier at this stage). Not applicable (used descriptively) |
| --- | --- | --- | --- | --- | --- |

preserve

. drop if p104_o1==.

(9,654 observations deleted)

. tab p104_o1

Justo antes o al momento de las |

relaciones sexuales que ha tenido en el |

último m | Freq. Percent Cum.

----------------------------------------+-----------------------------------

Alcohol (tres o más vasos) | 2,765 25.75 25.75

Cocaína | 29 0.27 26.02

Marihuana | 459 4.27 30.29

Drogas inyectables ¿Cuál? | 1 0.01 30.30

Tranquilizantes, ansiolíticos o antidep | 10 0.09 30.40

Alucinógenos, hongos | 3 0.03 30.42

Poppers | 4 0.04 30.46

Otra ¿cuál? | 8 0.07 30.54

No consumió ninguna | 7,459 69.46 100.00

----------------------------------------+-----------------------------------

Total | 10,738 100.00

. sum p104_o1

Variable | Obs Mean Std. dev. Min Max

-------------+---------------------------------------------------------

p104_o1 | 10,738 6.65813 3.562269 1 9

. restore

**24.Variable p119**

| p119 | En esa última relación sexual, ¿Ustedes usaron alguno método anticonceptivo? | Behavioural regulation | Dichotomous (Yes/No) | % of ‘Yes’ responses. Classified as: ≥70% = Enabler; 40–69% = Moderate Barrier; <40% = Major Barrier. | ≥ 70% → Enabler; 40–69% → Moderate Barrier; < 40% → Major Barrier |
| --- | --- | --- | --- | --- | --- |

preserve

. drop if p119==9

(139 observations deleted)

. drop if p119==.

(8,142 observations deleted)

. tab p119

En esa última |

relación sexual, |

¿Ustedes usaron |

alguno método |

anticonceptivo? | Freq. Percent Cum.

----------------------+-----------------------------------

Sí | 4,467 36.88 36.88

No | 7,644 63.12 100.00

----------------------+-----------------------------------

Total | 12,111 100.00

. sum p119

Variable | Obs Mean Std. dev. Min Max

-------------+---------------------------------------------------------

p119 | 12,111 1.631162 .4825099 1 2

. restore

.

**25.Variable p120**

| p120 | ¿Cuál o cuáles métodos anticonceptivos utilizaron? | Behavioural regulation | Categorical (Nominal) | Grouped into 12 theoretical categories | Contextual determinant (not classified as enabler/barrier at this stage). Not applicable (used descriptively) |
| --- | --- | --- | --- | --- | --- |

. preserve

. drop if p120_o1==.

(15,925 observations deleted)

. drop if p120_o1==99

(16 observations deleted)

. tab p120_o1

¿Cuál o cuáles métodos |

anticonceptivos utilizaron? | Freq. Percent Cum.

----------------------------------------+-----------------------------------

Condón o preservativo masculino | 2,332 52.39 52.39

Condón o preservativo femenino | 36 0.81 53.20

Píldora o pastillas anticonceptivas | 1,179 26.49 79.69

Dispositivo intrauterino (DIU, T de cob | 305 6.85 86.54

Implante | 210 4.72 91.26

Inyección | 310 6.96 98.23

Diafragma | 1 0.02 98.25

Coito interrumpido | 5 0.11 98.36

Método natural (Billings, ritmo) | 2 0.04 98.40

Esterilización femenina | 43 0.97 99.37

Esterilización masculina | 13 0.29 99.66

Otro ¿Cuál? | 15 0.34 100.00

----------------------------------------+-----------------------------------

Total | 4,451 100.00

. sum p120_o1

Variable | Obs Mean Std. dev. Min Max

-------------+---------------------------------------------------------

p120_o1 | 4,451 2.462368 1.984799 1 13

. restore

.

**26.Variable p121**

.

| p121 | (CONTESTAN P120=1,2) ¿Por qué motivo usaron preservativo o condón? | Goals | Dichotomous (Yes/No) | % of ‘Yes’ responses. Classified as: ≥70% = Enabler; 40–69% = Moderate Barrier; <40% = Major Barrier. | ≥ 70% → Enabler; 40–69% → Moderate Barrier; < 40% → Major Barrier |
| --- | --- | --- | --- | --- | --- |

preserve

. drop if p121a==.

(18,024 observations deleted)

. drop if p121a==9

(41 observations deleted)

. tab p121a

¿Por qué motivo |

usaron preservativo o |

condón? Para |

prevenir infecciones |

de tran | Freq. Percent Cum.

----------------------+-----------------------------------

Sí | 1,860 79.93 79.93

No | 467 20.07 100.00

----------------------+-----------------------------------

Total | 2,327 100.00

. sum p121a

Variable | Obs Mean Std. dev. Min Max

-------------+---------------------------------------------------------

p121a | 2,327 1.200688 .4006008 1 2

. restore

**27.Variable p122**

| p122 | ¿Dónde obtuvo el método o los métodos mencionados anteriormente? | Environmental context and resources | Categorical (Nominal) | Grouped into 6 theoretical categories | Contextual determinant (not classified as enabler/barrier at this stage). Not applicable (used descriptively) |
| --- | --- | --- | --- | --- | --- |

. preserve

. drop if p122==9

(56 observations deleted)

. drop if p122==.

(16,019 observations deleted)

. tab p122

¿Dónde obtuvo el método o |

los métodos mencionados |

anteriormente? | Freq. Percent Cum.

------------------------------+-----------------------------------

Consultorio | 1,420 32.89 32.89

Hospital | 171 3.96 36.85

Consulta privada | 408 9.45 46.31

Farmacia | 2,231 51.68 97.98

Se lo regaló o prestó alguien | 49 1.14 99.12

Otro lugar. ¿Cuál? | 38 0.88 100.00

------------------------------+-----------------------------------

Total | 4,317 100.00

. ///No tiene sentido este promedio

> sum p122

Variable | Obs Mean Std. dev. Min Max

-------------+---------------------------------------------------------

p122 | 4,317 2.868427 1.407091 1 6

. restore

.

. **28.Variable p123**

.

| p123 | (CONTESTAN SI P119=2) ¿Por qué razón no usó ningún método anticonceptivo? | Beliefs about capabilities | Categorical (Nominal) | Grouped into 6 theoretical categories | Contextual determinant (not classified as enabler/barrier at this stage). Not applicable (used descriptively) |
| --- | --- | --- | --- | --- | --- |

preserve

. drop if p123==9

(1,253 observations deleted)

. drop if p123==.

(12,748 observations deleted)

. tab p123

¿Por qué razón no usó ningún |

método anticonceptivo? | Freq. Percent Cum.

----------------------------------------+-----------------------------------

Buscaba embarazarse o embarazar a su pa | 363 5.68 5.68

No tenía disponible | 734 11.48 17.16

Olvido | 328 5.13 22.30

No quería | 3,892 60.90 83.20

Otro ¿Cuál? | 1,074 16.80 100.00

----------------------------------------+-----------------------------------

Total | 6,391 100.00

. ///No tiene sentido este promedio

> sum p123

Variable | Obs Mean Std. dev. Min Max

-------------+---------------------------------------------------------

p123 | 6,391 3.716633 1.053485 1 5

. restore

. **29.Variable p151**

| p151 | (RESPONDE SEXO = MUJER) ¿Alguna vez en su vida ha ido a una consulta o donde algún profesional de la salud para tratar asuntos médicos PROPIOS relacionados con la ginecología, pubertad, sexualidad, métodos preventivos del embarazo o infecciones de transmisión sexual? | Environmental context and resources | Dichotomous (Yes/No) | % of ‘Yes’ responses. Classified as: ≥70% = Enabler; 40–69% = Moderate Barrier; <40% = Major Barrier. | ≥ 70% → Enabler; 40–69% → Moderate Barrier; < 40% → Major Barrier |
| --- | --- | --- | --- | --- | --- |

. preserve

. drop if p151==9

(184 observations deleted)

. drop if p151==.

(6,838 observations deleted)

. tab p151

¿Alguna |

vez en su |

vida ha ido |

a una |

consulta o |

donde |

algún |

profesional |

de la sal | Freq. Percent Cum.

------------+-----------------------------------

Sí | 9,186 68.71 68.71

No | 4,184 31.29 100.00

------------+-----------------------------------

Total | 13,370 100.00

. sum p151

Variable | Obs Mean Std. dev. Min Max

-------------+---------------------------------------------------------

p151 | 13,370 1.312939 .4637073 1 2

. restore

.

. **30.Variable p152**

.

| p152 | RESPONDE SEXO = HOMBRE) ¿Alguna vez en su vida ha ido a una consulta o donde algún profesional de la salud para tratar asuntos médicos PROPIOS relacionados con la urología, pubertad, sexualidad, métodos preventivos del embarazo o infecciones de transmisión sexual? | Environmental context and resources | Dichotomous (Yes/No) | % of ‘Yes’ responses. Classified as: ≥70% = Enabler; 40–69% = Moderate Barrier; <40% = Major Barrier. | ≥ 70% → Enabler; 40–69% → Moderate Barrier; < 40% → Major Barrier |
| --- | --- | --- | --- | --- | --- |

preserve

. drop if p152==9

(121 observations deleted)

. drop if p152==.

(13,554 observations deleted)

. tab p152

¿Alguna |

vez en su |

vida ha ido |

a una |

consulta o |

donde |

algún |

profesional |

de la sal | Freq. Percent Cum.

------------+-----------------------------------

Sí | 1,633 24.31 24.31

No | 5,084 75.69 100.00

------------+-----------------------------------

Total | 6,717 100.00

. sum p152

Variable | Obs Mean Std. dev. Min Max

-------------+---------------------------------------------------------

p152 | 6,717 1.756886 .4289956 1 2

. restore

.

**31.Variable p154**

.

| p154 | ¿Por qué motivo fue esa primera vez? (MARQUE TODAS LAS QUE CONSIDERE) | Goals | Categorical (Nominal) | Grouped into 12 theoretical categories | Contextual determinant (not classified as enabler/barrier at this stage). Not applicable (used descriptively) |
| --- | --- | --- | --- | --- | --- |

. preserve

. drop if p154_o1==.

(9,572 observations deleted)

. drop if p154_o1==99

(84 observations deleted)

. tab p154_o1

¿Por qué motivo fue esa primera vez? | Freq. Percent Cum.

----------------------------------------+-----------------------------------

Para solicitar anticonceptivos, distint | 2,372 22.09 22.09

Para solicitar preservativos o condones | 213 1.98 24.08

Para solicitar la píldora del día despu | 60 0.56 24.64

Por información o consejería | 1,303 12.14 36.77

A hacer un chequeo preventivo | 2,231 20.78 57.55

Por un embarazo | 3,368 31.37 88.93

Por haber sufrido algún tipo de violenc | 22 0.20 89.13

Por tener síntomas de VIH-Sida o una in | 38 0.35 89.48

Para hacer un test VIH u otra infección | 32 0.30 89.78

Por una enfermedad ginecológica o uroló | 882 8.22 98.00

Sospecha de infertilidad | 63 0.59 98.58

Otro ¿cuál? | 152 1.42 100.00

----------------------------------------+-----------------------------------

Total | 10,736 100.00

. sum p154_o1

Variable | Obs Mean Std. dev. Min Max

-------------+---------------------------------------------------------

p154_o1 | 10,736 4.809613 2.679927 1 12

. restore

.

**32.Variable P155**

.

| p155 | ¿A qué lugar fue? | Environmental context and resources | Categorical (Nominal) | Grouped into 5 theoretical categories | Contextual determinant (not classified as enabler/barrier at this stage). Not applicable (used descriptively) |
| --- | --- | --- | --- | --- | --- |

preserve

. drop if p155==9

(37 observations deleted)

. drop if p155==.

(9,572 observations deleted)

. tab p155

¿A qué lugar fue? | Freq. Percent Cum.

----------------------------------------+-----------------------------------

CESFAM, CECOSF, consultorio, posta rura | 5,067 46.99 46.99

Consulta privada | 2,786 25.84 72.83

Hospital público | 1,664 15.43 88.26

Clínica privada | 1,215 11.27 99.53

Otro ¿cuál? | 51 0.47 100.00

----------------------------------------+-----------------------------------

Total | 10,783 100.00

. ///No tiene sentido este promedio

> sum p155

Variable | Obs Mean Std. dev. Min Max

-------------+---------------------------------------------------------

p155 | 10,783 1.923954 1.054429 1 5

. restore

.

**33.Variable p206**

.

| p206 | ¿Cómo descubrió que tenía alguna de esas infecciones? Si ha tenido más de una vez una infección, piense en la última. | Environmental context and resources | Categorical (Nominal) | Grouped into 5 theoretical categories | Contextual determinant (not classified as enabler/barrier at this stage). Not applicable (used descriptively) |
| --- | --- | --- | --- | --- | --- |

preserve

. drop if p206==9

(59 observations deleted)

. drop if p206==.

(18,975 observations deleted)

. tab p206

¿Cómo descubrió que tenía alguna de |

esas infecciones? Si ha tenido más de |

una ve | Freq. Percent Cum.

----------------------------------------+-----------------------------------

Una de sus parejas sexuales fue diagnos | 117 8.62 8.62

Usted tenía síntomas o malestares que l | 833 61.34 69.96

En una consulta por otro motivo de salu | 124 9.13 79.09

En un control de rutina | 228 16.79 95.88

De otra manera, ¿Cómo? | 56 4.12 100.00

----------------------------------------+-----------------------------------

Total | 1,358 100.00

> sum p206

Variable | Obs Mean Std. dev. Min Max

-------------+---------------------------------------------------------

p206 | 1,358 2.464654 1.002504 1 5

. restore

**34.Variable p207**

| p207 | La última vez que fue diagnosticado de alguna infección de transmisión sexual, ¿se lo informó a su o sus parejas sexuales? | Behavioural regulation | Ordinal (1–3) | % “Yes” = 70.33%. Classified as: ≥70% → Enabler; 40–69% → Moderate Barrier; <40% → Major Barrier | ≥ 70% → Enabler; 40–69% → Moderate Barrier; < 40% → Major Barrier |
| --- | --- | --- | --- | --- | --- |

. preserve

. drop if p207==8

(11 observations deleted)

. drop if p207==9

(41 observations deleted)

. drop if p207==.

(18,975 observations deleted)

. tab p207

La última vez que fue diagnosticado |

de alguna infección de transmisión |

sexual, ¿ | Freq. Percent Cum.

--------------------------------------+-----------------------------------

Sí | 960 70.33 70.33

No | 224 16.41 86.74

No tenía pareja sexual en ese momento | 181 13.26 100.00

--------------------------------------+-----------------------------------

Total | 1,365 100.00

. sum p207

Variable | Obs Mean Std. dev. Min Max

-------------+---------------------------------------------------------

p207 | 1,365 1.429304 .7145471 1 3

. restore

**35.Variable p208**

| p208 | Por cualquier razón, ¿Se ha hecho el examen del VIH o Sida en los últimos 12 meses? | Behavioural regulation | Dichotomous (Yes/No) | % of ‘Yes’ responses. Classified as: ≥70% = Enabler; 40–69% = Moderate Barrier; <40% = Major Barrier. | ≥ 70% → Enabler; 40–69% → Moderate Barrier; < 40% → Major Barrier |
| --- | --- | --- | --- | --- | --- |

.

. preserve

. drop if p208==8

(8 observations deleted)

. drop if p208==9

(399 observations deleted)

. drop if p208==.

(2,261 observations deleted)

. tab p208

Por |

cualquier |

razón, |

¿Se ha |

hecho el |

examen del |

VIH o Sida |

en los |

últimos 12 |

mes | Freq. Percent Cum.

------------+-----------------------------------

Sí | 4,004 22.59 22.59

No | 13,720 77.41 100.00

------------+-----------------------------------

Total | 17,724 100.00

. sum p208

Variable | Obs Mean Std. dev. Min Max

-------------+---------------------------------------------------------

p208 | 17,724 1.774092 .4181909 1 2

. restore

**36.Variable p210**

| p210 | ¿Por qué razón se hizo el examen del VIH o Sida? | Beliefs about consequences | Categorical (Nominal) | Grouped into 11 theoretical categories | Contextual determinant (not classified as enabler/barrier at this stage). Not applicable (used descriptively) |
| --- | --- | --- | --- | --- | --- |

.

. preserve

. drop if p210==99

(21 observations deleted)

. drop if p210==.

(16,388 observations deleted)

. tab p210

¿Por qué razón se hizo el examen del |

VIH o Sida? | Freq. Percent Cum.

----------------------------------------+-----------------------------------

Por haber estado en situación de riesgo | 148 3.72 3.72

Porque lo hace como una rutina | 2,334 58.60 62.31

Por inicio de una nueva relación de par | 63 1.58 63.90

Por una donación de sangre | 226 5.67 69.57

Por control de embarazo | 394 9.89 79.46

Por una hospitalización/cirugía | 166 4.17 83.63

Por problemas de salud | 207 5.20 88.83

Por exigencia para ingresar a un trabaj | 126 3.16 91.99

Por exigencia para contratar un seguro | 16 0.40 92.39

Por exigencia para ingresar como estudi | 10 0.25 92.64

Otra. Especifique | 293 7.36 100.00

----------------------------------------+-----------------------------------

Total | 3,983 100.00

. sum p210

Variable | Obs Mean Std. dev. Min Max

-------------+---------------------------------------------------------

p210 | 3,983 3.715541 2.765882 1 11

. restore

**37.Variable p211**

.

| p211 | [SOLO PARA P202.10 = 2 & P208 = 2] ¿Por qué razón no se ha hecho el examen del VIH o Sida en los últimos 12 meses? MARCAR TODAS LAS QUE CORRESPONDA | Beliefs about consequences | Categorical (Nominal) | Grouped into 7 theoretical categories | Contextual determinant (not classified as enabler/barrier at this stage). Not applicable (used descriptively) |
| --- | --- | --- | --- | --- | --- |

. preserve

. drop if p211_o1==.

(6,672 observations deleted)

. drop if p211_o1==9

(366 observations deleted)

. tab p211_o1

¿Por qué razón no se ha hecho el |

examen del VIH o Sida en los últimos |

12 meses? | Freq. Percent Cum.

----------------------------------------+-----------------------------------

Por no estar en situación de riesgo | 9,121 68.30 68.30

Por no considerarlo necesario porque te | 2,208 16.53 84.84

Por no saber dónde hacerlo | 252 1.89 86.72

Por no haberlo pensado o no considerarl | 1,182 8.85 95.57

Por el temor a un resultado positivo | 32 0.24 95.81

Por miedo o vergüenza a ser estigmatiza | 49 0.37 96.18

Otra. Especifique | 510 3.82 100.00

----------------------------------------+-----------------------------------

Total | 13,354 100.00

. sum p211_o1

Variable | Obs Mean Std. dev. Min Max

-------------+---------------------------------------------------------

p211_o1 | 13,354 1.7257 1.419822 1 7

. restore

.

.

**38.Variable i_1_p212**

| i_1_p212 | Ahora le voy a hacer unas preguntas sobre VIH o Sida. Cree usted qué…¿Puede reducirse el riesgo de transmisión del VIH manteniendo relaciones sexuales con una única pareja fiel y sin VIH o Sida? | Knowledge | Dichotomous (Yes/No) | % of ‘Yes’ responses. Classified as: ≥70% = Enabler; 40–69% = Moderate Barrier; <40% = Major Barrier. | ≥ 70% → Enabler; 40–69% → Moderate Barrier; < 40% → Major Barrier |
| --- | --- | --- | --- | --- | --- |

. preserve

. drop if i_1_p212==8

(7 observations deleted)

. drop if i_1_p212==9

(1,164 observations deleted)

. tab i_1_p212

(¿Puede |

reducirse |

el riesgo |

de |

transmisió |

n del VIH |

manteniendo |

relaciones |

sexual | Freq. Percent Cum.

------------+-----------------------------------

Sí | 14,685 76.40 76.40

No | 4,536 23.60 100.00

------------+-----------------------------------

Total | 19,221 100.00

. sum i_1_p212

Variable | Obs Mean Std. dev. Min Max

-------------+---------------------------------------------------------

i_1_p212 | 19,221 1.235992 .4246282 1 2

. restore

**39.Variable i_2_p212**

.

| i_2_p212 | ¿Puede reducirse el riesgo de transmisión del VIH usando preservativo o condón cada vez que se mantienen relaciones sexuales? | Knowledge | Dichotomous (Yes/No) | % of ‘Yes’ responses. Classified as: ≥70% = Enabler; 40–69% = Moderate Barrier; <40% = Major Barrier. | ≥ 70% → Enabler; 40–69% → Moderate Barrier; < 40% → Major Barrier |
| --- | --- | --- | --- | --- | --- |

. preserve

. drop if i_2_p212==9

(1,268 observations deleted)

. tab i_2_p212

(¿Puede |

reducirse |

el riesgo |

de |

transmisió |

n del VIH |

usando |

preservativ |

o o condón | Freq. Percent Cum.

------------+-----------------------------------

Sí | 16,281 85.13 85.13

No | 2,843 14.87 100.00

------------+-----------------------------------

Total | 19,124 100.00

. sum i_2_p212

Variable | Obs Mean Std. dev. Min Max

-------------+---------------------------------------------------------

i_2_p212 | 19,124 1.148661 .3557637 1 2

. restore

. **40.Variable i_3_p212**

| i_3_p212 | ¿Puede una persona de aspecto saludable tener VIH? | Knowledge | Dichotomous (Yes/No) | % of ‘Yes’ responses. Classified as: ≥70% = Enabler; 40–69% = Moderate Barrier; <40% = Major Barrier. | ≥ 70% → Enabler; 40–69% → Moderate Barrier; < 40% → Major Barrier |
| --- | --- | --- | --- | --- | --- |

. preserve

. drop if i_3_p212==9

(1,794 observations deleted)

. tab i_3_p212

(¿Puede |

una persona |

de aspecto |

saludable |

tener VIH?) |

Ahora le |

voy a hacer |

unas p | Freq. Percent Cum.

------------+-----------------------------------

Sí | 15,931 85.66 85.66

No | 2,667 14.34 100.00

------------+-----------------------------------

Total | 18,598 100.00

. sum i_3_p212

Variable | Obs Mean Std. dev. Min Max

-------------+---------------------------------------------------------

i_3_p212 | 18,598 1.143403 .3504923 1 2

. restore

**41.Variable i_4_p212**

| i_4_p212 | ¿Se puede adquirir el VIH por picaduras de mosquito? | Knowledge | Dichotomous (Yes/No) | % of ‘No’ responses. Classified as: ≥70% = Enabler; 40–69% = Moderate Barrier; <40% = Major Barrier. | ≥ 70% → Enabler; 40–69% → Moderate Barrier; < 40% → Major Barrier |
| --- | --- | --- | --- | --- | --- |

.

. preserve

. drop if i_4_p212==9

(4,511 observations deleted)

. tab i_4_p212

(¿Se puede |

adquirir el |

VIH por |

picaduras |

de |

mosquito?) |

Ahora le |

voy a hacer |

unas | Freq. Percent Cum.

------------+-----------------------------------

Sí | 4,041 25.45 25.45

No | 11,840 74.55 100.00

------------+-----------------------------------

Total | 15,881 100.00

. sum i_4_p212

Variable | Obs Mean Std. dev. Min Max

-------------+---------------------------------------------------------

i_4_p212 | 15,881 1.745545 .4355681 1 2

. restore

**42.Variable i_5_p212**

.

| i_5_p212 | ¿Se puede adquirir el VIH por compartir alimentos con una persona con VIH/Sida? | Knowledge | Dichotomous (Yes/No) | % of ‘No’ responses. Classified as: ≥70% = Enabler; 40–69% = Moderate Barrier; <40% = Major Barrier. | ≥ 70% → Enabler; 40–69% → Moderate Barrier; < 40% → Major Barrier |
| --- | --- | --- | --- | --- | --- |

. preserve

. drop if i_5_p212==9

(2,385 observations deleted)

. tab i_5_p212

(¿Se puede |

adquirir el |

VIH por |

compartir |

alimentos |

con una |

persona con |

VIH/Sida? | Freq. Percent Cum.

------------+-----------------------------------

Sí | 3,181 17.67 17.67

No | 14,826 82.33 100.00

------------+-----------------------------------

Total | 18,007 100.00

. sum i_5_p212

Variable | Obs Mean Std. dev. Min Max

-------------+---------------------------------------------------------

i_5_p212 | 18,007 1.823346 .3813858 1 2

. restore

**43.Variable i_6_p212**

.

| i_6_p212 | ¿Se puede transmitir el VIH de la madre al niño/a en el embarazo, parto o durante la lactancia? | Knowledge | Dichotomous (Yes/No) | % of ‘Yes’ responses. Classified as: ≥70% = Enabler; 40–69% = Moderate Barrier; <40% = Major Barrier. | ≥ 70% → Enabler; 40–69% → Moderate Barrier; < 40% → Major Barrier |
| --- | --- | --- | --- | --- | --- |

. preserve

. drop if i_6_p212==9

(2,966 observations deleted)

. tab i_6_p212

(¿Se puede |

transmitir |

el VIH de |

la madre al |

niño/a en |

el |

embarazo, |

parto o |

duran | Freq. Percent Cum.

------------+-----------------------------------

Sí | 14,290 82.00 82.00

No | 3,136 18.00 100.00

------------+-----------------------------------

Total | 17,426 100.00

. sum i_6_p212

Variable | Obs Mean Std. dev. Min Max

-------------+---------------------------------------------------------

i_6_p212 | 17,426 1.179961 .384166 1 2

. restore

**44.Variable p213**

| p213 | ¿Conoce usted la medida “profilaxis de preexposición” o PREP como alternativa de prevención del VIH/Sida? | Knowledge | Dichotomous (Yes/No) | % of ‘Yes’ responses. Classified as: ≥70% = Enabler; 40–69% = Moderate Barrier; <40% = Major Barrier. | ≥ 70% → Enabler; 40–69% → Moderate Barrier; < 40% → Major Barrier |
| --- | --- | --- | --- | --- | --- |

.

. preserve

. drop if p213==9

(1,628 observations deleted)

. tab p213

¿Conoce |

usted la |

medida |

“profilax |

is de |

preexposici |

ón” o |

PREP como |

alternativa |

de | Freq. Percent Cum.

------------+-----------------------------------

Sí | 1,971 10.50 10.50

No | 16,793 89.50 100.00

------------+-----------------------------------

Total | 18,764 100.00

. sum p213

Variable | Obs Mean Std. dev. Min Max

-------------+---------------------------------------------------------

p213 | 18,764 1.894958 .3066151 1 2

. restore

.

.

.

.

.

.

.

.

end of do-file

.

| **TDF domain** | **Description** |
| --- | --- |
| Knowledge | An awareness of the existence of something |
| Skills | An ability or proficiency acquired through practice |
| Social/professional role and identity | A coherent set of behaviors and displayed personal qualities of an individual in a social or work setting |
| Beliefs about capabilities | Acceptance of the truth, reality, or validity about an ability, talent, or facility that a person can put to constructive use |
| Optimism | The confidence that things will happen for the best, or that desired goals will be attained |
| Beliefs about consequences | Acceptance of the truth, reality, or validity about outcomes of a behavior in a given situation |
| Reinforcement | Increasing the probability of a response by arranging a dependent relationship, or contingency, between the response and a given stimulus |
| Intentions | A conscious decision to perform a behavior or a resolve to act in a certain way |
| Goals | Mental representation of outcomes or end states that an individual wants to achieve |
| Memory, attention and decision processes | The ability to retain information, focus selectively on aspects of the environment, and choose between two or more alternatives |
| Environmental context and resources | Any circumstance of a person’s situation or environment that discourages or encourages the development of skills and abilities, independence, social competence, and adaptive behavior |
| Social influences | Those interpersonal processes that can cause an individual to change their thoughts, feelings, or behaviors |
| Emotion | A complex reaction pattern, involving experiential, behavioral, and physiological elements, by which the individual attempts to deal with a personally significant matter or event |
| Behavioral regulation | Anything aimed at managing or changing objectively observed or measured actions |

# Appendix 7. 405 items Included in the National Survey

| **N°** | **VARIABLE** | **LABEL ( questions of the questionnaire)** |
| --- | --- | --- |
| 1 | sexo [Sex] | - |
| 2 | edad [Age] | - |
| 3 | p1 | ¿Cuál es su sexo asignado al nacer? [What is your sex assigned at birth?] |
| 4 | p2 | ¿Es usted una persona intersex? [Are you an intersex person?] |
| 5 | p3 | ¿Cuál es el género con el que Usted se identifica? [What gender do you identify with?] |
| 6 | p3_otro | - |
| 7 | p4 | ¿Qué edad tiene? [How old are you?] |
| 8 | p5 | ¿Cuál es su nivel educacional más alto alcanzado o su nivel educacional actual? [What is your highest level of education attained or your current educational level?] |
| 9 | p6 | En ese nivel educacional, ¿cuál fue el último curso que aprobó (para los que no... [At that educational level, what was the last grade you passed (for those who did not...] |
| 10 | p7 | ¿Cuál es su estado conyugal o civil actual? [What is your current marital or civil status?] |
| 11 | p8 | ¿Cómo calificaría su calidad de vida? [How would you rate your quality of life?] |
| 12 | i_1_p9 | (Con la privacidad que tiene donde vive) Ahora le preguntaré acerca de cómo se siente. [With the privacy you have where you live, I will now ask you how you feel.] |
| 13 | i_2_p9 | (Con su bienestar mental o emocional) Ahora le preguntaré acerca de cómo se siente. [With your mental or emotional well-being, I will now ask you how you feel.] |
| 14 | i_3_p9 | (Con su vida amorosa) Ahora le preguntaré acerca de cómo se siente. [With your love life, I will now ask you how you feel.] |
| 15 | i_4_p9 | (Con la cantidad de diversión que tiene en su vida) Ahora le preguntaré acerca de cómo se siente. [With the amount of fun you have in your life, I will now ask you how you feel.] |
| 16 | i_5_p9 | (Con su vida familiar) Ahora le preguntaré acerca de cómo se siente Usted en distintos aspectos. [With your family life, I will now ask you how you feel in different aspects.] |
| 17 | i_6_p9 | (Con su vida sexual) Ahora le preguntaré acerca de cómo se siente Usted en distintos aspectos. [With your sex life, I will now ask you how you feel in different aspects.] |
| 18 | p10 | En general Usted diría que su salud es... [In general, you would say that your health is...] |
| 19 | i_1_p11 | (Poco interés o placer en hacer cosas) Durante las dos últimas semanas, ¿con qué frecuencia ha sentido molestias, debido a cualquiera de los siguientes problemas? [Little interest or pleasure in doing things: In the last two weeks, how often have you felt distress due to any of the following problems?] |
| 20 | i_2_p11 | (Se ha sentido decaído(a), deprimido(a) o sin esperanzas) Durante las dos últimas semanas, ¿con qué frecuencia ha sentido molestias, debido a cualquiera de los siguientes problemas? [Feeling down, depressed, or hopeless: In the last two weeks, how often have you felt distress due to any of the following problems?] |
| 21 | i_3_p11 | (Se ha sentido nervioso(a), ansioso(a) o con los nervios de punta) Durante las dos últimas semanas, ¿con qué frecuencia ha sentido molestias, debido a cualquiera de los siguientes problemas? [Feeling nervous, anxious, or on edge: In the last two weeks, how often have you felt distress due to any of the following problems?] |
| 22 | i_4_p11 | (No ha sido capaz de parar o controlar su preocupación) Durante las dos últimas semanas, ¿con qué frecuencia ha sentido molestias, debido a cualquiera de los siguientes problemas? [Not being able to stop or control worrying: In the last two weeks, how often have you felt distress due to any of the following problems?] |
| 23 | i_1_p12 | ¿Alguna vez en su vida ha tenido la experiencia A? Pensar seriamente en suicidarse. [Have you ever in your life had experience A? Seriously thinking about suicide.] |
| 24 | i_1_p13 | ¿Alguna vez en su vida ha tenido la experiencia B? Hacer un plan para suicidarse. ¿Ha tenido la experiencia B en los últimos 12 meses? [Have you ever in your life had experience B? Making a plan to commit suicide. Have you had experience B in the last 12 months?] |
| 25 | i_2_p12 | (Experiencia B.) Ahora vea la tarjeta 6. Están anotadas dos experiencias designadas A y B. [Experience B. Now look at card 6. Two designated experiences A and B are written.] |
| 26 | i_2_p13 | (Experiencia B.) ¿Ha tenido la experiencia... en los últimos 12 meses? [Experience B. Have you had the experience... in the last 12 months?] |
| 27 | i_1_p16 | (Ver, sin usar anteojos ópticos o lentes?) En los últimos 30 días y sin tener en cuenta el uso de lentes. [Seeing, without using glasses or lenses? In the last 30 days and disregarding the use of glasses.] |
| 28 | i_2_p16 | (Oír, sin usar audífono o dispositivo de ayuda para oír?) En los últimos 30 días. [Hearing, without using a hearing aid or hearing device? In the last 30 days.] |
| 29 | i_3_p16 | (Caminar o subir peldaños?) En los últimos 30 días y sin tener en cuenta ningún dispositivo de ayuda. [Walking or climbing stairs? In the last 30 days and without considering any assistive device.] |
| 30 | i_4_p16 | ¿Recordar cosas o concentrarse? [Remembering things or concentrating?] |
| 31 | i_5_p16 | (Asearse o vestirse?) En los últimos 30 días y sin tener en cuenta ningún tipo de ayuda [Grooming or dressing? In the last 30 days and without any kind of help] |
| 32 | i_6_p16 | (Dormir?) En los últimos 30 días y sin tener en cuenta ningún tipo de ayuda [Sleeping? In the last 30 days and without any kind of help] |
| 33 | i_7_p16 | (Hacer las tareas de la casa como barrer, cocinar, hacer arreglos o sacar la basura) [Doing household chores such as sweeping, cooking, doing repairs or taking out the garbage] |
| 34 | i_8_p16 | (Participar en fiestas, eventos religiosos, reuniones vecinales u otras actividades) [Participating in parties, religious events, neighborhood meetings, or other activities] |
| 35 | i_9_p16 | ¿Llevarse bien con la gente cercana a usted, incluyendo su familia, amigos y amigas? [Getting along with people close to you, including family, friends, and others?] |
| 36 | i_1_p17 | Debido a su salud, ¿qué grado de dificultad le ha generado en su vida… (Sentirse triste, deprimido(a), preocupado(a) o ansioso(a)?) [Due to your health, how much difficulty has it caused you in your life… (Feeling sad, depressed, worried, or anxious?)] |
| 37 | i_2_p17 | Debido a su salud, ¿qué grado de dificultad le ha generado en su vida… ¿Sentir algún malestar o dolor físico, por ejemplo, dolor de espalda, dolor de estómago o dolor de cabeza? [Due to your health, how much difficulty has it caused you in your life… Feeling some discomfort or physical pain, such as back pain, stomach pain or headache?] |
| 38 | i_1_p18a1 | A continuación, le voy a preguntar por algunas enfermedades que Usted tiene o ha tenido (Migrañas o dolores de cabeza frecuentes) ¿Alguna vez un doctor o médico le ha dicho que tiene o que padece de...? [I will now ask you about some illnesses that you have or have had (Migraines or frequent headaches). Has a doctor or physician ever told you that you have or suffer from...?] |
| 39 | i_1_p18b1 | (Migrañas o dolores de cabeza frecuentes) ¿A qué edad se la diagnosticaron... [Migraines or frequent headaches: At what age were you diagnosed...?] |
| 40 | i_1_p18c1 | (Migrañas o dolores de cabeza frecuentes) ¿Alguna vez ha sido tratado por esto... [Migraines or frequent headaches: Have you ever been treated for this...?] |
| 41 | i_1_p18d1 | (Migrañas o dolores de cabeza frecuentes) ¿Alguna vez ha sido operado por esto... [Migraines or frequent headaches: Have you ever had surgery for this...?] |
| 42 | i_1_p18e1 | (Migrañas o dolores de cabeza frecuentes) ¿Ha estado tomando algún medicamento o [Migraines or frequent headaches: Have you been taking any medication or] |
| 43 | i_2_p18a1 | (Trastorno musculo esquelético (dolor de espalda crónico, pierna, brazos, hombro...¿Alguna vez un doctor o médico le ha dicho que tiene o que padece de...? [Musculoskeletal disorder (chronic pain in the back, legs, arms, shoulder)... Has a doctor ever told you that you have or suffer from...?] |
| 44 | i_2_p18b1 | (Trastorno musculo esquelético) ¿A qué edad se la diagnosticaron? [Musculoskeletal disorder: At what age were you diagnosed?] |
| 45 | i_2_p18c1 | (Trastorno musculo esquelético) ¿Alguna vez ha sido tratado por esto...? [Musculoskeletal disorder: Have you ever been treated for this...?] |
| 46 | i_2_p18d1 | (Trastorno musculo esquelético) ¿Alguna vez ha sido operado por esto...? [Musculoskeletal disorder: Have you ever had surgery for this...?] |
| 47 | i_2_p18e1 | (Trastorno musculo esquelético) ¿Ha estado tomando algún medicamento o [Musculoskeletal disorder: Have you been taking any medication or] |
| 48 | i_1_p18a2 | (Depresión) ¿Alguna vez un doctor o médico le ha dicho que tiene o que padece de [Depression: Has a doctor or physician ever told you that you have or suffer from] |
| 49 | i_1_p18b2 | (Depresión) ¿A qué edad se la diagnosticaron [Depression: At what age were you diagnosed?] |
| 50 | i_1_p18c2 | (Depresión) ¿Alguna vez ha sido tratado por esto... [Depression: Have you ever been treated for this?] |
| 51 | i_1_p18e2 | (Depresión) ¿Ha estado tomando algún medicamento o haciendo algún tratamiento [Depression: Have you been taking any medication or undergoing any treatment?] |
| 52 | i_1_p18a3 | (Diabetes) ¿Alguna vez un doctor o médico le ha dicho que tiene o que padece de. [Diabetes: Has a doctor ever told you that you have or suffer from it?] |
| 53 | i_1_p18b3 | (Diabetes) ¿A qué edad se la diagnosticaron... [Diabetes: At what age were you diagnosed?] |
| 54 | i_1_p18c3 | (Diabetes) ¿Alguna vez ha sido tratado [Diabetes: Have you ever been treated for it?] |
| 55 | i_1_p18d3 | (Diabetes) ¿Alguna vez ha sido operado por [Diabetes: Have you ever had surgery for it?] |
| 56 | i_1_p18e3 | (Diabetes) ¿Ha estado tomando algún medicamento o haciendo algún tratamiento pa [Diabetes: Have you been taking any medication or undergoing any treatment for it?] |
| 57 | i_2_p18a3 | (Bocio, enfermedad a la tiroides hipertiroidismo o hipotiroidismo) ¿Alguna vez u [Goiter, thyroid disease: hyperthyroidism or hypothyroidism – Has a doctor ever told you that you have or suffer from it?] |
| 58 | i_2_p18b3 | (Bocio, enfermedad a la tiroides hipertiroidismo o hipotiroidismo) ¿A qué edad s [Goiter, thyroid disease: At what age were you diagnosed?] |
| 59 | i_2_p18c3 | (Bocio, enfermedad a la tiroides hipertiroidismo o hipotiroidismo) ¿Alguna vez ha sido tratado [Goiter, thyroid disease: Have you ever been treated for it?] |
| 60 | i_2_p18d3 | (Bocio, enfermedad a la tiroides hipertiroidismo o hipotiroidismo) ¿Alguna vez ha sido operado [Goiter, thyroid disease: Have you ever had surgery for it?] |
| 61 | i_2_p18e3 | (Bocio, enfermedad a la tiroides hipertiroidismo o hipotiroidismo) ¿Ha estado to [Goiter, thyroid disease: Have you been taking any medication or undergoing any treatment for it?] |
| 62 | i_3_p18a3 | (Sobrepeso u obesidad) ¿Alguna vez un doctor o médico le ha dicho que tiene o qu [Overweight or obesity: Has a doctor ever told you that you have or suffer from it?] |
| 63 | i_3_p18b3 | (Sobrepeso u obesidad) ¿A qué edad se la diagnosticaron... @_@ITERNAME [Overweight or obesity: At what age were you diagnosed?] |
| 64 | i_3_p18c3 | (Sobrepeso u obesidad) ¿Alguna vez ha sido tratado por esto... [Overweight or obesity: Have you ever been treated for this?] |
| 65 | i_3_p18d3 | (Sobrepeso u obesidad) ¿Alguna vez ha sido operado [Overweight or obesity: Have you ever had surgery for it?] |
| 66 | i_3_p18e3 | (Sobrepeso u obesidad) ¿Ha estado tomando algún medicamento o haciendo algún tra [Overweight or obesity: Have you been taking any medication or undergoing any treatment for it?] |
| 67 | i_4_p18a3 | (Hipertensión) ¿Alguna vez un doctor o médico le ha dicho que tiene o que padece [Hypertension: Has a doctor ever told you that you have or suffer from it?] |
| 68 | i_4_p18b3 | (Hipertensión) ¿A qué edad se la diagnosticaron... [Hypertension: At what age were you diagnosed?] |
| 69 | i_4_p18c3 | (Hipertensión) ¿Alguna vez ha sido tratado por esto... [Hypertension: Have you ever been treated for this?] |
| 70 | i_4_p18d3 | (Hipertensión) ¿Alguna vez ha sido operado por esto... [Hypertension: Have you ever had surgery for this?] |
| 71 | i_4_p18e3 | (Hipertensión) ¿Ha estado tomando algún medicamento o haciendo algún tratamiento [Hypertension: Have you been taking any medication or undergoing any treatment?] |
| 72 | i_5_p18a3 | (Infertilidad) ¿Alguna vez un doctor o médico le ha dicho que tiene o que padece [Infertility: Has a doctor ever told you that you have this?] |
| 73 | i_5_p18b3 | (Infertilidad) ¿A qué edad se la diagnosticaron [Infertility: At what age were you diagnosed?] |
| 74 | i_5_p18c3 | (Infertilidad) ¿Alguna vez ha sido tratado por esto... @_@I [Infertility: Have you ever been treated for this?] |
| 75 | i_5_p18d3 | (Infertilidad) ¿Alguna vez ha sido operado por esto... [Infertility: Have you ever had surgery for this?] |
| 76 | i_5_p18e3 | (Infertilidad) ¿Ha estado tomando algún [Infertility: Have you been taking any medication for this?] |
| 77 | p19 | ¿Ha sido diagnosticado con COVID-19? [Have you been diagnosed with COVID-19?] |
| 78 | p20 | ¿Alguna vez se ha realizado una mamografía? [Have you ever had a mammogram?] |
| 79 | p20b | ¿Alguna vez se ha realizado un papanicolaou? [Have you ever had a Pap smear?] |
| 80 | p20c | ¿Alguna vez se ha realizado un control de antígeno prostático? [Have you ever had a prostate-specific antigen (PSA) test?] |
| 81 | p21a | ¿En los últimos tres años se ha realizado una mamografía? [In the past three years, have you had a mammogram?] |
| 82 | p21b | ¿En los últimos tres años se ha realizado un papanicolaou? [In the past three years, have you had a Pap smear?] |
| 83 | p21c | ¿En los últimos tres años se ha realizado un control de antígeno prostático? [In the past three years, have you had a PSA test?] |
| 84 | p22 | ¿Me podría decir cuánto pesa aproximadamente actualmente? [Could you tell me your approximate current weight?] |
| 85 | p23 | ¿Me podría decir cuánto mide aproximadamente actualmente? [Could you tell me your approximate current height?] |
| 86 | i_1_p24 | (Me gusta mi aspecto físico tal y como es) ¿Qué tan de acuerdo está con las sigu [I like the way my body looks just as it is – To what extent do you agree with the following] |
| 87 | i_2_p24 | (Me gusta el aspecto de mi cuerpo sin la ropa)¿Qué tan de acuerdo está con las sigu [I like how my body looks without clothes – To what extent do you agree with the following] |
| 88 | i_3_p24 | (Soy físicamente atractivo/a) ¿Qué tan de acuerdo está con las siguientes afirma [I am physically attractive – To what extent do you agree with the following statements] |
| 89 | i_1_p25 | (Cocaína) ¿Ha probado Ud. alguna de las siguientes sustancias alguna vez en su v [Cocaine: Have you ever tried any of the following substances in your life?] |
| 90 | i_1_p26 | (Cocaína) ¿Cuándo fue la última vez que consumió? [Cocaine: When was the last time you used it?] |
| 91 | i_2_p25 | (Marihuana) ¿Ha probado Ud. alguna de las siguientes sustancias alguna vez en su vida? [Marijuana: Have you ever tried any of the following substances in your life?] |
| 92 | i_2_p26 | (Marihuana) ¿Cuándo fue la última vez que consumió? [Marijuana: When was the last time you used it?] |
| 93 | i_3_p25 | (Drogas inyectables) ¿Ha probado Ud. alguna de las siguientes sustancias alguna vez en su vida? [Injectable drugs: Have you ever tried any of the following substances in your life?] |
| 94 | i_3_p26 | (Drogas inyectables) ¿Cuándo fue la última vez que consumió? [Injectable drugs: When was the last time you used it?] |
| 95 | i_4_p25 | (Tranquilizantes, ansiolíticos o antidepresivo) ¿Ha probado Ud. alguna de las siguientes sustancias alguna vez en su vida? [Tranquilizers, anxiolytics or antidepressants: Have you ever tried any of the following substances in your life?] |
| 96 | i_4_p26 | (Tranquilizantes, ansiolíticos o antidepresivo) ¿Cuándo fue la última vez que consumió? [Tranquilizers, anxiolytics or antidepressants: When was the last time you used it?] |
| 97 | i_5_p25 | (Alcohol) ¿Ha probado Ud. alguna de las siguientes sustancias alguna vez en su vida? [Alcohol: Have you ever tried any of the following substances in your life?] |
| 98 | i_5_p26 | ¿Cuándo fue la última vez que consumió? [When was the last time you used it?] |
| 99 | i_6_p25 | (Alucinógenos, hongos) ¿Ha probado Ud. alguna de las siguientes sustancias alguna vez en su vida? [Hallucinogens, mushrooms: Have you ever tried any of the following substances in your life?] |
| 100 | i_6_p26 | (Alucinógenos, hongos) ¿Cuándo fue la última vez que consumió? [Hallucinogens, mushrooms: When was the last time you used it?] |
| 101 | i_7_p25 | (Poppers) ¿Ha probado Ud. alguna de las siguientes sustancias alguna vez en su vida? [Poppers: Have you ever tried any of the following substances in your life?] |
| 102 | i_7_p26 | (Poppers) ¿Cuándo fue la última vez que consumió? [Poppers: When was the last time you used it?] |
| 103 | i_8_p25 | (Otra) ¿Ha probado Ud. alguna de las siguientes sustancias alguna vez en su vida? [Other: Have you ever tried any of the following substances in your life?] |
| 104 | i_8_p26 | (Otra) ¿Cuándo fue la última vez que consumió? [Other: When was the last time you used it?] |
| 105 | p25_otra1 | ¿Cuál? Drogas inyectables [Which one? Injectable drugs] |
| 106 | p25_otra2 | ¿Cuál? Otra [Which one? Other] |
| 107 | p27 | Si usted pudiera volver a la época en que todavía no tenía hijos/as y pudiera elegir exactamente el número de hijos/as que tendría en toda su vida ¿Cuántos serían? [If you could go back to the time before you had children and choose exactly how many children you would have in your life, how many would that be?] |
| 108 | i_1_p28 | Respecto a la sexualidad y el género… ¿Cuán de acuerdo está Ud. con...? Hay que tener un orgasmo para que una relación sexual sea verdaderamente satisfactoria. [Regarding sexuality and gender… To what extent do you agree with the statement: One must have an orgasm for a sexual relationship to be truly satisfying?] |
| 109 | i_2_p28 | Es necesario mantener relaciones sexuales regularmente para el bienestar personal. [It is necessary to have regular sexual activity for personal well-being.] |
| 110 | i_3_p28 | Los hombres por naturaleza tienen más necesidades sexuales que las mujeres. [Men naturally have more sexual needs than women.] |
| 111 | i_4_p28 | La homosexualidad es una forma de vivir la sexualidad tan válida como cualquier otra. [Homosexuality is a way of living sexuality just as valid as any other.] |
| 112 | i_5_p28 | Una mujer debe tener derecho a interrumpir un embarazo, si no desea ser madre o no desea serlo en ese momento o circunstancia. [A woman should have the right to terminate a pregnancy if she does not want to be a mother or does not wish to be one at that time or under those circumstances.] |
| 113 | i_6_p28 | La masturbación es una práctica aceptable teniendo pareja. [Masturbation is an acceptable practice when in a relationship.] |
| 114 | i_7_p28 | Solo se debe tener sexo cuando hay amor. [One should only have sex when there is love.] |
| 115 | p29 | ¿Usted conoce personalmente a personas lesbianas, gay, bisexual, queer, intersex, trans, etc.? [Do you personally know any lesbian, gay, bisexual, queer, intersex, or trans people?] |
| 116 | p30_o1 | ¿Y de dónde conoce a esa/a persona/s? [And where do you know this person/these people from?] |
| 117 | p31 | Si uno de sus hijos/as le dijera que es homosexual (gay), lesbiana, bisexual u otra, usted.... [If one of your children told you they were gay, lesbian, bisexual or other, you would…] |
| 118 | p32 | Si uno de sus hijos/as le dijera que es trans (transgénero masculino, transgénero femenino), usted…: (LEA ALTERNATIVAS) [If one of your children told you they were transgender (male-to-female or female-to-male), you would… (READ OPTIONS)] |
| 119 | i_1_p33 | (Usar preservativos o condón disminuye el placer de las mujeres.) [Using condoms reduces women's sexual pleasure.] |
| 120 | i_2_p33 | (Usar preservativos o condón disminuye el placer de los hombres.) [Using condoms reduces men's sexual pleasure.] |
| 121 | i_3_p33 | (Es necesario ocupar preservativo o condón incluso si se tiene pareja estable) [It is necessary to use condoms even in a stable relationship.] |
| 122 | i_4_p33 | (Usar preservativo o condón estimula el juego sexual.) [Using condoms stimulates sexual play.] |
| 123 | i_5_p33 | (Los preservativos o condones son demasiado caros para usarlos regularmente) [Condoms are too expensive to use regularly.] |
| 124 | p34 | Cuando usted era niño/a, ¿En su familia se conversaban temas sexuales? [When you were a child, were sexual topics discussed in your family?] |
| 125 | p35 | (CONTESTAN P34=2,3) ¿Y con qué frecuencia usted participaba cuando se conversaban temas sexuales? [And how often did you participate in conversations about sexual topics?] |
| 126 | t_p36_1 | En su escuela, cuándo usted era estudiante, ¿se impartía educación sexual en Enseñanza básica? [When you were a student, was sex education taught in primary school?] |
| 127 | t_p36_2 | En su escuela, cuándo usted era estudiante, ¿se impartía educación sexual en Enseñanza media? [When you were a student, was sex education taught in high school?] |
| 128 | p37 | Y respecto de esa educación sexual, en general, usted cree que... [And regarding that sex education, in general, do you think that...] |
| 129 | p38 | ¿Cómo evaluaría EN GENERAL la FORMACIÓN EN SEXUALIDAD que recibió en su colegio o escuela? [How would you evaluate the SEXUALITY EDUCATION you received at school overall?] |
| 130 | i_1_p39 | ¿Qué nota le podría ahora al conocimiento que usted tenía en los siguientes temas, cuando era adolescente, donde 1 es bajo conocimiento y 7 alto conocimiento? (Métodos para prevenir un embarazo) [What grade would you give your knowledge as a teenager on the following topic: methods to prevent pregnancy? (1 = low, 7 = high)] |
| 131 | i_2_p39 | (Métodos para prevenir infecciones de transmisión sexual) ¿Qué nota le pondría a [What grade would you give your knowledge as a teenager on the following topic: methods to prevent sexually transmitted infections?] |
| 132 | i_1_p40_o1 | Cuando usted era adolescente, a raíz de alguna duda sobre los siguientes temas, ¿A quién recurrió para resolverlas? Relaciones sexuales [As a teenager, when you had questions about the following topics, who did you go to for answers? Sexual relations] |
| 133 | i_2_p40_o1 | Cuando usted era adolescente, a raíz de alguna duda sobre los siguientes temas, ¿A quién recurrió para resolverlas? (Métodos preventivos de embarazo) [As a teenager, when you had questions about the following topics, who did you go to for answers? (Pregnancy prevention methods)] |
| 134 | i_3_p40_o1 | Cuando usted era adolescente, a raíz de alguna duda sobre los siguientes temas, ¿A quién recurrió para resolverlas? (Métodos preventivos de infecciones de transmisión sexual, como el VIH) [As a teenager, when you had questions about the following topics, who did you go to for answers? (STI prevention methods, such as HIV)] |
| 135 | i_1_p41 | ¿Qué tan de acuerdo o en desacuerdo está con que la educación sexual en Chile tenga las siguientes características? Que promueva la abstinencia sexual hasta, a lo menos, los 18 años. [To what extent do you agree or disagree that sex education in Chile should include: Promoting sexual abstinence until at least 18 years old.] |
| 136 | i_2_p41 | (Que transmita una visión positiva del placer y el disfrute sexual.) [Conveying a positive view of pleasure and sexual enjoyment.] |
| 137 | i_3_p41 | (Que enseñe que solo un hombre y una mujer pueden conformar una familia) [Teaching that only a man and a woman can form a family.] |
| 138 | i_4_p41 | (Que enseñe a poner un preservativo o condón) [Teaching how to put on a condom.] |
| 139 | i_5_p41 | (Que enseñe que se tiene que acordar claramente que se quiere tener relaciones sexuales con la otra persona) [Teaching that there must be clear mutual agreement to have sex.] |
| 140 | p42 | (Si usted es mujer) ¿A qué edad aproximadamente tuvo su primera menstruación o regla? [If you are a woman: At approximately what age did you have your first menstruation or period?] |
| 141 | p43 | (Si es hombre) ¿A qué edad aproximadamente tuvo su primera eyaculación nocturna? [If you are a man: At approximately what age did you have your first nocturnal ejaculation?] |
| 142 | p44 | (Para mujeres) ¿Usted conversó con alguien sobre su primera menstruación o regla en ese momento? [Did you talk to anyone about your first menstruation or period at that time?] |
| 143 | p45 | (Para hombres) ¿Usted conversó con alguien sobre su primera eyaculación nocturna en ese momento? [Did you talk to anyone about your first nocturnal ejaculation at that time?] |
| 144 | p46 | ¿Qué edad tenía usted cuando dio su primer beso en la boca con connotación romántico o sexual? [How old were you when you had your first kiss on the mouth with romantic or sexual connotation?] |
| 145 | t_p47_1 | ¿Alguna vez en su vida ha tenido relaciones sexuales voluntarias? [Have you ever had voluntary sexual intercourse in your life?] |
| 146 | t_p47_2 | ¿Alguna vez en su vida ha tenido las siguientes prácticas sexuales voluntarias? Sexo vaginal [Have you ever had the following voluntary sexual practices in your life? Vaginal sex] |
| 147 | t_p47_3 | ¿Alguna vez en su vida ha tenido las siguientes prácticas sexuales voluntarias? Sexo anal [Have you ever had the following voluntary sexual practices in your life? Anal sex] |
| 148 | p48 | ¿Alguna vez en su vida ha tenido las siguientes prácticas sexuales voluntarias? Sexo oral [Have you ever had the following voluntary sexual practices in your life? Oral sex] |
| 149 | p49 | (Para quienes no han tenido relaciones sexuales) ¿Ha recibido caricias en el cuerpo, caricias en pechos o caricias genitales? [Have you received caresses on your body, breasts, or genitals?] |
| 150 | p50_o1 | ¿A qué edad tuvo su primera relación sexual voluntaria? [At what age did you have your first voluntary sexual intercourse?] |
| 151 | p51 | ¿Esta primera relación sexual, de qué forma fue? Sexo vaginal [What form did your first sexual intercourse take? Vaginal sex] |
| 152 | p52 | ¿La persona con quien tuvo esa primera relación sexual era hombre, mujer, otro? [Was the person with whom you had your first sexual intercourse a man, woman, or other?] |
| 153 | p53 | ¿Y la otra persona, qué edad tenía? [And how old was the other person?] |
| 154 | p54 | Antes de su primera relación sexual, ¿Usted y esa persona hablaron de cómo evitar un posible embarazo? [Before your first sexual intercourse, did you and that person talk about how to avoid a possible pregnancy?] |
| 155 | p55 | Antes de su primera relación sexual, ¿Usted y esa persona hablaron de cómo evitar una ITS…? [Before your first sexual intercourse, did you and that person talk about how to prevent an STI?] |
| 156 | p56 | En esa primera relación sexual, ¿Ustedes usaron algún método anticonceptivo? [Did you use any contraceptive method during your first sexual intercourse?] |
| 157 | p57 | ¿Cuál o cuáles métodos anticonceptivos usaron en esa primera relación sexual? MARQUE TODAS LAS QUE CORRESPONDA. [Which contraceptive method(s) did you use during your first sexual intercourse? MARK ALL THAT APPLY.] |
| 158 | p58 | ¿Cuál fue el principal motivo por el cual usaron preservativo o condón? [What was the main reason you used a condom?] |
| 159 | p59 | ¿Dónde obtuvo el método o los métodos mencionados anteriormente? [Where did you get the method(s) mentioned above?] |
| 160 | p60 | ¿En qué lugar tuvo su primera relación sexual? [Where did your first sexual intercourse take place?] |
| 161 | p61 | ¿Era la primera relación sexual también para la otra persona? [Was it also the other person's first sexual experience?] |
| 162 | p62 | ¿Qué tipo de vínculo tenía usted en ese momento con la persona con quien tuvo esa primera relación sexual? (LEA) [What was your relationship with the person with whom you had your first sexual intercourse? (READ)] |
| 163 | p63 | ¿Su primera relación sexual fue principalmente por…?: MARQUE UNA ALTERNATIVA [Your first sexual intercourse was mainly because…?: MARK ONE OPTION] |
| 164 | p64 | Después de la primera relación sexual, ¿Usted tuvo otras relaciones sexuales con la misma persona? [After the first sexual intercourse, did you have sex again with the same person?] |
| 165 | p65 | Con la persona con la que usted, tuvo esa primera relación sexual ¿Llegaron a vivir en pareja? [Did you end up living as a couple with the person with whom you had your first sexual intercourse?] |
| 166 | p66 | (CONTESTAN P65=1) ¿Cuánto tiempo duró la relación de pareja con esta persona? (RECORDAR QUE ES EN RELACIÓN CON LA PAREJA DE LA PRIMERA RELACIÓN SEXUAL) [How long did your relationship with this person last? (REMEMBER: This refers to the partner of your first sexual intercourse)] |
| 167 | p67 | En los seis meses siguientes a esa relación sexual, ¿habló con alguien que no fuera su pareja respecto de esa primera relación sexual? [In the six months after your first sexual intercourse, did you talk to someone other than your partner about it?] |
| 168 | p68 | ¿Con quién habló primero sobre esa primera relación sexual? MARQUE UNA ALTERNATIVA [Who did you talk to first about your first sexual intercourse? MARK ONE OPTION] |
| 169 | p69 | En el transcurso de toda su vida, ¿podría indicar con cuántas personas ha tenido relaciones sexuales? Se trata de personas con las que tuvo relaciones sexuales, aun cuando no hayan sido «pareja». Se incluyen parejas ocasionales, parejas de una sola noche y trabajadoras/es sexuales (“prostitutas/os”). No olvidar a la pareja actual. Si no recuerda con exactitud, por favor indique un número aproximado. [Over the course of your life, how many people have you had sexual relations with? This includes people who were not your partner—such as one-night stands, casual partners, and sex workers ("prostitutes"). Don’t forget your current partner. If you don’t remember exactly, please give an approximate number.] |
| 170 | p70 | (CONTESTAN P69>0) En el transcurso de toda su vida, ¿cuántos han sido hombres, mujeres, u otro [Of the sexual partners you've had in your life, how many were men, women, or other?] |
| 171 | p71 | En el transcurso del último año, ¿podría indicar con cuántas personas han tenido relaciones sexuales? Se trata de personas con las que se tuvo relaciones sexuales, aun cuando no hayan constituido «pareja». Se incluyen parejas ocasionales, parejas de una sola noche y trabajadoras/es sexuales (“prostitutas/os”). No olvidar a la pareja actual. Si no recuerda con exactitud, por favor indique un número aproximado. [In the past year, how many people have you had sexual relations with? This includes people who were not your partner—such as one-night stands, casual partners, and sex workers ("prostitutes"). Don’t forget your current partner. If you don’t remember exactly, please give an approximate number.] |
| 172 | p72 | (CONTESTAN P71>0 Y EN P70 SE MARCA MÁS DE UN SEXO) En el transcurso del último año, ¿cuántos han sido hombres, mujeres, u otro? [In the past year, how many of those sexual partners were men, women, or other?] |
| 173 | p73 | (CONTESTAN P71>0) En las relaciones con esas parejas sexuales del último año, ¿con qué frecuencia usted usaba condón o preservativo? (LEA LAS ALTERNATIVAS) [With those sexual partners from the past year, how often did you use condoms? (READ THE OPTIONS)] |
| 174 | p74 | (CONTESTAN P71>0) En el último mes, ¿podría indicar con cuántas personas ha tenido relaciones sexuales? Se trata de personas con las que tuvo relaciones sexuales, aun cuando no hayan sido «pareja». Se incluyen parejas ocasionales, parejas de una sola noche y trabajadoras/es sexuales (“prostitutas/os”). No olvidar a la pareja actual. Si no recuerda con exactitud, por favor indique un número aproximado. [In the last month, could you indicate how many people you had sexual intercourse with? This includes people with whom you had sexual intercourse even if they were not a “partner.” It includes casual partners, one-night stands, and sex workers (“prostitutes”). Don't forget your current partner. If you don’t remember exactly, please provide an approximate number.] |
| 175 | p75 | (CONTESTAN P74>0 Y EN P72 SE MARCA MÁS DE UN SEXO) En el último mes, ¿cuántos han sido hombres, mujeres, u otro? [In the last month, how many were men, women, or other?] |
| 176 | p76 | (CONTESTAN P74>0) En el último mes, ¿cuántas relaciones sexuales tuvo? Considere número de relaciones sexuales (no parejas sexuales) (Si no ha tenido marcar 0). [In the last month, how many times did you have sexual intercourse? Count the number of sexual acts (not sexual partners). (If none, mark 0).] |
| 177 | p77 | En relación con la cantidad de relaciones sexuales que tuvo el último mes, ¿cuál de las siguientes afirmaciones lo/la representa mejor? [Regarding the number of sexual encounters you had last month, which of the following statements best represents you?] |
| 178 | p78 | En el transcurso de toda su vida, ¿podría indicar con cuántas personas ha tenido una relación afectiva importante, hayan o no vivido juntos? (pololos/as, novios/as, esposo/a, etc.) [Over the course of your life, how many people have you had a significant romantic relationship with, whether or not you lived together? (boyfriends/girlfriends, partners, spouses, etc.)] |
| 179 | p79 | En el transcurso de toda su vida, ¿cuántos han sido hombres, mujeres, u otro? [Throughout your life, how many have been men, women, or other?] |
| 180 | p80 | De ellas, ¿con cuántas ha vivido en pareja durante al menos 3 meses? Considere convivencia sin acuerdo de unión civil, convivencia con acuerdo de unión civil y matrimonio. [Of them, how many have you lived with as a couple for at least 3 months? Consider cohabitation without civil union, with civil union agreement, and marriage.] |
| 181 | p81 | Actualmente, ¿Usted tiene pareja? [Do you currently have a partner?] |
| 182 | p82 | ¿Desde qué año usted tiene esa pareja? [Since what year have you had this partner?] |
| 183 | p83 | (CONTESTA SI P81=1) ¿Y actualmente usted vive con esa pareja? [And do you currently live with this partner?] |
| 184 | p84 | (CONTESTA SI P83 = 1) ¿Desde qué año viven juntos? [Since what year have you been living together?] |
| 185 | p85 | (CONTESTA SI P83 = 2) ¿Usted tiene la intención de vivir junto a su pareja algún día? [Do you intend to live with your partner someday?] |
| 186 | p86 | (CONTESTA SI P81 = 1) Su relación de pareja actual se caracteriza por ser… [Your current relationship is characterized by being...] |
| 187 | p87 | ¿En los últimos 5 años ha tenido alguna separación o ruptura de una relación amorosa? [In the last 5 years, have you experienced a separation or breakup of a romantic relationship?] |
| 188 | p88 | ¿Volvió a tener relaciones sexuales con esa persona después de la separación? (Enc: en caso de haber tenido más de una pareja de la cual se separó, responder respecto a la más importante para la persona entrevistada). [Did you have sexual intercourse with that person again after the separation? (Note for interviewer: if the person had more than one partner from whom they separated, refer to the most important one for the interviewee).] |
| 189 | p89 | Y en la primera relación sexual que volvió a tener con (NOMBRE PAREJA SEPARACIÓN) después de la separación ¿usaron condón o preservativo? [And in the first sexual encounter you had again with (NAME OF SEPARATED PARTNER) after the separation, did you use a condom?] |
| 190 | p90 | (CONTESTA SI P81=1) Ahora hablaremos de la persona con la que usted vive, o bien, con quién tiene una relación estable e importante, aunque no vivan juntos. ¿Esa persona es hombre, mujer u otro? [Now let's talk about the person you live with or with whom you have a stable and significant relationship, even if you don’t live together. Is that person a man, woman, or other?] |
| 191 | p91 | ¿Qué edad tiene esa pareja? (INDICAR EDAD EN AÑOS CUMPLIDOS) [How old is your partner? (INDICATE AGE IN COMPLETED YEARS)] |
| 192 | p92 | (CONTESTA SI P81=1) Comparando con su pareja actual, el ingreso económico de Usted es: [Compared to your current partner, your economic income is:] |
| 193 | p93 | (CONTESTA SI P81=1) ¿Diría que usted depende económicamente de su pareja? [Would you say you are economically dependent on your partner?] |
| 194 | p94 | (CONTESTA SI P83=1) Actualmente, ¿Usted duerme…? (LEA ALTERNATIVAS) [Currently, do you sleep...? (READ ALTERNATIVES) (ASKED IF P83=1)] |
| 195 | p95 | ¿Usted y su pareja hablan o han hablado sobre si desean o desearon tener hijos/as? [Have you and your partner talked or ever talked about whether you want(ed) to have children?] |
| 196 | p96 | (CONTESTA SI P81=1) En el último mes, ¿tuvo relaciones sexuales con su pareja? [In the past month, have you had sexual intercourse with your partner? (ASKED IF P81=1)] |
| 197 | p97 | (CONTESTA SI P81=1 & P96=2) ¿Eso es así por qué…? [Is that so because...? (ASKED IF P81=1 & P96=2)] |
| 198 | p98 | En el transcurso de su vida, ¿alguna vez se ha masturbado? [In your lifetime, have you ever masturbated?] |
| 199 | p99 | ¿A qué edad se masturbó Usted por primera vez? Si no recuerda, mencione una edad aproximada [At what age did you masturbate for the first time? If you don’t remember, mention an approximate age] |
| 200 | p100 | En el último mes, ¿cuántas veces se ha masturbado aproximadamente? [In the past month, approximately how many times have you masturbated?] |
| 201 | p101 | En los últimos 12 meses ¿Con qué frecuencia usted ha realizado las siguientes prácticas en sus relaciones sexuales? [In the past 12 months, how often have you practiced the following during your sexual relations?] |
| 202 | p102 | En el transcurso de su vida…. [In your lifetime....] |
| 203 | p103 | (CONTESTAN SI P74 >0. DROGAS SE FILTRAN SEGÚN REPORTADO EN P25) Justo antes o al momento de las relaciones sexuales que ha tenido en el último mes, ¿usted consumió alguna de las siguientes sustancias? [Just before or at the time of the sexual intercourse you had in the past month, did you consume any of the following substances? (ASKED IF P74>0, FILTERED BY P25 DRUGS)] |
| 204 | p104 | (CONTESTAN SI P74 >0) Y alguna de las personas con la que usted estaba, ¿había consumido alguna de las siguientes sustancias? (MULTIPLE) [And did any of the persons you were with consume any of the following substances? (MULTIPLE OPTIONS) (ASKED IF P74>0)] |
| 205 | p105 | En los últimos 12 meses, ¿Con qué frecuencia ha visto material pornográfico (tales como películas, videos, imágenes, etc.)? [In the past 12 months, how often have you watched pornographic material (such as movies, videos, images, etc.)?] |
| 206 | p106 | ¿Alguna vez en su vida usted ha enviado imágenes o videos suyas con contenido sexual o erótico por algún medio digital (mensaje de texto, e-mail, mensaje directo, WhatsApp, etc.) [Have you ever in your life sent images or videos of yourself with sexual or erotic content through any digital means (text message, e-mail, direct message, WhatsApp, etc.)?] |
| 207 | p107 | ¿Alguna vez en su vida usted ha recibido imágenes de conocidos con contenido sexual o erótico por algún medio digital (mensaje de texto, e-mail, mensaje directo, WhatsApp, etc.)? [Have you ever in your life received sexual or erotic content from people you know through digital means (text message, e-mail, direct message, WhatsApp, etc.)?] |
| 208 | p108 | ¿Alguna vez en su vida ha usado video llamadas (mostrando sus genitales, pechos, o realizando alguna actividad sexual)? [Have you ever in your life used video calls to show your genitals, breasts, or perform any sexual activity?] |
| 209 | p109 | En el último año, ¿ha buscado parejas afectivas y/o sexuales por medio de aplicaciones o plataformas de encuentro digitales? [In the past year, have you searched for romantic and/or sexual partners through digital platforms or apps?] |
| 210 | p110 | (CONTESTA SI P109=1) ¿Tuvo relaciones sexuales con esta o estas personas? [Did you have sexual intercourse with this or these people? (ASKED IF P109=1)] |
| 211 | p111 | ¿Alguna vez ha pagado para tener relaciones sexuales? [Have you ever paid to have sex?] |
| 212 | p112 | (CONTESTA SI P111=1) ¿Qué edad tenía en ese momento? [How old were you at that time? (ASKED IF P111=1)] |
| 213 | p113 | (CONTESTA SI P111=2) ¿Qué edad tenía la primera vez? Y ¿Qué edad tenía la última vez? [How old were you the first time? And how old were you the last time? (ASKED IF P111=2)] |
| 214 | p114 | (CONTESTA SI P111=2) ¿Usted tenía pareja la última vez que pagó para tener relaciones sexuales? [Did you have a partner the last time you paid for sex? (ASKED IF P111=2)] |
| 215 | p115 | Vamos a hablar ahora de su última relación sexual. ¿Cuándo fue su última relación sexual? PARA ENTREVISTADO/AR/A: PUEDE SER LA PAREJA U OTRA PERSONA. [Let's now talk about your last sexual encounter. When was your last sexual encounter? FOR RESPONDENT: IT MAY BE WITH A PARTNER OR SOMEONE ELSE.] |
| 216 | p116 | ¿Qué tipo de vínculo tenía usted en ese momento con la persona con quien tuvo esa última relación sexual? (LEA ALTERNATIVAS) [What kind of relationship did you have at the time with the person you had that last sexual encounter with? (READ ALTERNATIVES)] |
| 217 | p117 | ¿La persona con quien tuvo esa última relación sexual era hombre, mujer, otro? [Was the person you had your last sexual encounter with a man, woman, or other?] |
| 218 | p118 | En cuanto a su género, ¿esa persona es?: [Regarding their gender, is that person?:] |
| 219 | p119 | En esa última relación sexual, ¿Ustedes usaron alguno método anticonceptivo? [In that last sexual encounter, did you use any contraceptive method?] |
| 220 | p120 | ¿Cuál o cuáles métodos anticonceptivos utilizaron? MARQUE TODAS LAS QUE CORRESPONDAN [Which contraceptive methods did you use? MARK ALL THAT APPLY] |
| 221 | p121 | (CONTESTAN P120=1,2) ¿Por qué motivo usaron preservativo o condón? [Why did you use a condom? (ASKED IF P120=1,2)] |
| 222 | p122 | ¿Dónde obtuvo el método o los métodos mencionados anteriormente? [Where did you obtain the method(s) mentioned above?] |
| 223 | p123 | (CONTESTAN SI P119=2) ¿Por qué razón no usó ningún método anticonceptivo? [Why didn’t you use any contraceptive method? (ASKED IF P119=2)] |
| 224 | p124 | ¿Tuvo alguna dificultad para conseguir o acceder a métodos anticonceptivos durante la pandemia? [Did you have any difficulty getting or accessing contraceptive methods during the pandemic?] |
| 225 | p125 | ¿Esta última relación sexual, de qué forma fue? MARQUE TODAS LAS QUE CORRESPONDAN. [What sexual practices were involved in this last sexual encounter? MARK ALL THAT APPLY.] |
| 226 | p126 | [SOLO PARA 125=3] ¿Usted le hizo sexo oral a esta persona? [Did you perform oral sex on this person? (ONLY IF 125=3)] |
| 227 | p127 | [SOLO PARA 125=3] ¿Esta persona le hizo sexo oral a usted? [Did this person perform oral sex on you? (ONLY IF 125=3)] |
| 228 | p128 | Sexo vaginal Sexo anal Sexo Oral Prefiero no responder [Vaginal sex Anal sex Oral sex Prefer not to answer] |
| 229 | p129 | Y la otra persona, ¿Alcanzó un orgasmo? [Did the other person reach orgasm?] |
| 230 | p130 | ¿Y qué tan satisfecho está con esta última relación sexual? [And how satisfied are you with this last sexual encounter?] |
| 231 | p131 | (CONTESTAN SI P115=5) ¿Cuál es el principal motivo por el que usted no ha tenido relaciones sexuales en el último año? [What is the main reason you have not had sex in the past year? (ASKED IF P115=5)] |
| 232 | p132 | (CONTESTAN SI P115=4) ¿Qué diría sobre la situación de no tener relaciones sexuales en el último año? [What would you say about not having had sex in the past year? (ASKED IF P115=4)] |
| 233 | p133 | Durante su vida, usted se ha sentido atraído/a sexualmente por… Por favor no lea, solo indíqueme el número de la tarjeta. (ENCUESTADOR: SOLO PIDA NÚMERO DE RESPUESTA AL ENTREVISTADO/A) [In your lifetime, have you felt sexually attracted to... Please don’t read aloud, just tell me the number on the card. (INTERVIEWER: ONLY ASK FOR THE RESPONSE NUMBER FROM THE RESPONDENT)] |
| 234 | p134 | Usted actualmente se identifica cómo: [How do you currently identify yourself?] |
| 235 | p135 | Durante su vida, ¿ha tenido una orientación sexual diferente de la que recién me comentó (homosexual, heterosexual bisexual u otro)? [During your life, have you ever had a sexual orientation different from the one you just mentioned (homosexual, heterosexual, bisexual, or other)?] |
| 236 | p136 | (CONTESTAN P134 = 1, 2 o 3) ¿A qué edad, se sintió sexualmente atraído, por primera vez, por alguien de su mismo sexo? [At what age did you first feel sexually attracted to someone of the same sex? (ASKED IF P134 = 1, 2 or 3)] |
| 237 | p137 | (CONTESTAN P134 = 1, 2 o 3) ¿Alguna vez recibió tratamiento de alguien que intentó cambiar su orientación sexual (como tratar de volverse heterosexual)? En caso afirmativo, marque todas las alternativas que correspondan. [Have you ever received treatment from someone who tried to change your sexual orientation (such as trying to make you heterosexual)? If yes, mark all that apply. (ASKED IF P134 = 1, 2 or 3)] |
| 238 | p138 | (CONTESTAN P3 = 3, 4 Y 5) Anteriormente me mencionó que no se identifica con el género que le asignaron en su nacimiento. ¿A qué edad comenzó a sentir que su género era “diferente” al que le asignaron en su nacimiento? [Earlier you mentioned that you do not identify with the gender assigned to you at birth. At what age did you begin to feel that your gender was “different” from the one assigned at birth? (ASKED IF P3 = 3, 4 or 5)] |
| 239 | p139 | (CONTESTAN P3= 3 o 4) respecto a su identidad de género, ¿diría que está o estuvo en proceso de transición? [Regarding your gender identity, would you say you are or have been in the process of transition? (ASKED IF P3 = 3 or 4)] |
| 240 | p140 | (CONTESTAN P3= 3 o 4) ¿Desde hace cuántos años está en proceso de transición? (indique cantidad de años aproximado) [For how many years have you been in the process of transition? (Indicate approximate number of years) (ASKED IF P3 = 3 or 4)] |
| 241 | p141 | (CONTESTAN P3 = 3 o 4 & P139 = 1) Actualmente, ¿ha tenido o ha comenzado? [Currently, have you had or begun...? (ASKED IF P3 = 3 or 4 & P139 = 1)] |
| 242 | p142 | (CONTESTAN P3 = 3 o 4 & P141.1 = 1) ¿Ha efectuado su cambio de nombre y sexo en el Registro Civil? [Have you changed your name and sex in the Civil Registry? (ASKED IF P3 = 3 or 4 & P141.1 = 1)] |
| 243 | p143 | (CONTESTAN P3 = 3 o 4 & 141.2 = 1) Si está tomando hormonas, ¿por dónde las obtiene? (marque todas las que corresponde) [If you are taking hormones, where do you get them from? (Mark all that apply) (ASKED IF P3 = 3 or 4 & P141.2 = 1)] |
| 244 | p144 | (CONTESTAN P3 = 3 o 4 & 141.3 = 1) ¿Ha tenido operaciones o intervenciones quirúrgicas relacionadas con una reasignación de sexo o transición de género? [Have you had surgeries or procedures related to sex reassignment or gender transition? (ASKED IF P3 = 3 or 4 & P141.3 = 1)] |
| 245 | p145 | (CONTESTAN P144 = 1) ¿Cuál/es es/son la/s operación/es o intervención/es de otra índole que se ha realizado? (Puedes marcar más de una alternativa) [Which surgery or other procedures have you undergone? (You can mark more than one) (ASKED IF P144 = 1)] |
| 246 | p146 | (CONTESTAN P144 = 1) ¿Dónde se realizó esas operaciones o intervenciones quirúrgicas? (Puedes marcar más de una alternativa) [Where did you have those surgeries or procedures performed? (You can mark more than one) (ASKED IF P144 = 1)] |
| 247 | p147 | (CONTESTAN P3 = 3 o 4 & P141.1 = 1 \| P141.2 = 1 \| P141.3 = 1) Durante su proceso de transición ¿ha tenido la necesidad de una consulta de diagnóstico para solicitar certificado de disforia de género con algún profesional de salud (médico psiquiatra o psicólogo/a)? [During your transition, have you needed a diagnostic consultation to request a gender dysphoria certificate from a health professional (psychiatrist or psychologist)? (ASKED IF P3 = 3 or 4 & P141.1/2/3 = 1)] |
| 248 | p148 | (CONTESTAN P3 = 3º 4) ¿Alguna vez recibió tratamiento de alguien que intentó hacerle identificarse solo con su sexo asignado al nacer (en otras palabras, tratar de evitar que usted fuera transgénero)? En caso afirmativo, marque todas las casillas que correspondan. [Have you ever received treatment from someone who tried to make you identify only with your birth-assigned sex (i.e., to prevent you from being transgender)? If yes, check all that apply. (ASKED IF P3 = 3 or 4)] |
| 249 | p149 | (CONTESTAN P3 = 3, 4, 5, 6, 7 o P134 = 1, 3, 4) A continuación, te presentamos varias frases que describen diversas experiencias de vida y cosas que pueden haberte ocurrido por ser una persona LGTBQ+. Desde que tenías 18 años, ¿Alguna vez…? [Here are several statements describing life experiences that may have happened to you as an LGBTQ+ person. Since you were 18, have you ever...? (ASKED IF P3 = 3, 4, 5, 6, 7 or P134 = 1, 3, 4)] |
| 250 | p150 | [SOLO PARA P149 = 1] ¿Y cuántas veces ha sucedido en los últimos 12 meses? [How many times has this happened in the last 12 months? (ONLY IF P149 = 1)] |
| 251 | p151 | (RESPONDE SEXO = MUJER) ¿Alguna vez en su vida ha ido a una consulta o donde algún profesional de la salud para tratar asuntos médicos PROPIOS relacionados con la ginecología, pubertad, sexualidad, métodos preventivos del embarazo o infecciones de transmisión sexual? [Have you ever in your life visited a health professional to address personal medical issues related to gynecology, puberty, sexuality, pregnancy prevention methods, or sexually transmitted infections? (RESPONDENT SEX = FEMALE)] |
| 252 | p152 | (RESPONDE SEXO = HOMBRE) ¿Alguna vez en su vida ha ido a una consulta o donde algún profesional de la salud para tratar asuntos médicos PROPIOS relacionados con la urología, pubertad, sexualidad, métodos preventivos del embarazo o infecciones de transmisión sexual? [Have you ever in your life visited a health professional to address personal medical issues related to urology, puberty, sexuality, pregnancy prevention methods, or sexually transmitted infections? (RESPONDENT SEX = MALE)] |
| 253 | p153 | ¿Qué edad tenía la primera vez que consultó?: [How old were you the first time you sought such a consultation?] |
| 254 | p154 | ¿Por qué motivo fue esa primera vez? (MARQUE TODAS LAS QUE CONSIDERE) [What was the reason for that first time? (MARK ALL THAT APPLY)] |
| 255 | p155 | ¿A qué lugar fue? (LEA ALTERNATIVAS. CIRCULE SOLO UNO) [Where did you go? (READ OPTIONS. CIRCLE ONLY ONE)] |
| 256 | p156 | [SOLO PARA SEXO = MUJER] ¿Alguna vez en su vida ha tenido un control o consulta ginecológica? [FOR WOMEN ONLY: Have you ever had a gynecological check-up or consultation?] |
| 257 | p157 | [SOLO PARA SEXO ASIGNADO AL NACER = MUJER] Te pedimos que nos indiques si, en el marco de tus visitas a servicios de ginecología (públicos o privados), con qué frecuencia le ha ocurrido alguna de las siguientes situaciones. [FOR PEOPLE ASSIGNED FEMALE AT BIRTH ONLY: Please indicate how often the following situations have occurred during your visits to gynecological services (public or private).] |
| 258 | p158 | ¿Ha estado embarazada alguna vez en su vida? (incluir embarazos que terminaron en parto normal, parto prematuro, aborto, muerte fetal u otro problema). [Have you ever been pregnant? (Include pregnancies ending in full-term delivery, preterm delivery, abortion, stillbirth, or other complications).] |
| 259 | p159 | ¿Está embarazada actualmente? [Are you currently pregnant?] |
| 260 | p160 | ¿Cuántos embarazos ha tenido a lo largo de su vida? incluya embarazos que terminaron en parto normal, parto prematuro, aborto, muerte fetal u otro problema. [How many pregnancies have you had in your lifetime? Include pregnancies ending in full-term delivery, preterm delivery, abortion, stillbirth, or other complications.] |
| 261 | p161 | ¿Uno o más de los embarazos que tuvo no fue planificado o no estaba previsto que ocurriera? [Was one or more of your pregnancies unplanned or unexpected?] |
| 262 | p162 | ¿Cuántos embarazos fueron no planificados? [How many pregnancies were unplanned?] |
| 263 | p163 | ¿Cuál o cuáles? [Which one(s)?] |
| 264 | p164 | (CONTESTAN SI P161=1) ¿Qué edad tenía usted al momento que se enteró del primer embarazo que no fue planificado o no había previsto que ocurriera? [(IF P161=1) How old were you when you found out about your first unplanned or unexpected pregnancy?] |
| 265 | p165 | (CONTESTAN SI P161=1) En el momento en que se enteró de este embarazo no planificado o no previsto ¿Qué relación tenía usted con la persona con la que tuvo este embarazo? [(IF P161=1) At the time you found out about this unplanned pregnancy, what was your relationship with the person you had the pregnancy with?] |
| 266 | p166 | (CONTESTAN SI P161=1) ¿Usted o esa persona usaban un método preventivo del embarazo en el momento en que tuvo ese primer embarazo que no fue planificado o no estaba previsto que ocurriera? [(IF P161=1) Were you or that person using any pregnancy prevention method when you had that first unplanned pregnancy?] |
| 267 | p167 | ¿Qué edad tenía usted cuando ocurrió el primer embarazo? [How old were you when the first pregnancy occurred?] |
| 268 | p168 | ¿Quién era la persona de la cual se embarazó? Considere la relación que tenía usted con esa persona en el momento del embarazo. [Who was the person you got pregnant by? Consider the relationship you had with that person at the time of the pregnancy.] |
| 269 | p169 | ¿Y cuántos/as hijos/as nacidos vivos/as ha tenido Usted? [How many live-born children have you had?] |
| 270 | p170 | (SI TIENE HIJOS/AS) ¿Qué edad tenía usted cuando nació su primer/a hijo/a? [IF YOU HAVE CHILDREN: How old were you when your first child was born?] |
| 271 | p171 | (SI TIENE HIJOS/AS) ¿Con cuántas personas distintas ha tenido hijos/as? [IF YOU HAVE CHILDREN: How many different people have you had children with?] |
| 272 | p172 | (SI TIENE HIJOS/AS), ¿Cuántos de sus hijos viven con usted? [IF YOU HAVE CHILDREN: How many of your children live with you?] |
| 273 | p173 | ¿En este hogar hay niños menores de 3 años a su cuidado? [Are there children under 3 years old in this household under your care?] |
| 274 | p174 | Ahora vamos a conversar sobre el último embarazo que tuvo. ¿Se realizó un control prenatal, se chequeó el embarazo alguna vez? [Now we are going to talk about your last pregnancy. Did you have a prenatal check-up, did you have the pregnancy checked at any time?] |
| 275 | p175 | ¿En qué semana de su último embarazo tuvo el primer control? [In which week of your last pregnancy did you have your first check-up?] |
| 276 | p176 | ¿Cuántos controles prenatales tuvo Ud. durante el último embarazo? [How many prenatal check-ups did you have during your last pregnancy?] |
| 277 | p177 | ¿Durante su último embarazo con quién se controló? (MARQUE TODAS LAS QUE CONSIDERE) [During your last pregnancy, who did you consult with for check-ups? (MARK ALL THAT APPLY)] |
| 278 | p178 | ¿Dónde se controló? (MARQUE TODAS LAS QUE CONSIDERE) [Where did you have your check-ups? (MARK ALL THAT APPLY)] |
| 279 | p179 | En alguno de sus controles de su último embarazo, le hicieron o le mandaron a usted lo siguiente: [During any of your check-ups in your last pregnancy, were you given or referred for the following:] |
| 280 | p180 | Su último embarazo terminó con: [Your last pregnancy ended in:] |
| 281 | p181 | (CONTESTAN SI P0=1 o 2) ¿Cuál es la fecha de su último parto? SI NO SABE O NO RECUERDA, COLOCAR 01/01/1900. [(IF P0=1 or 2) What is the date of your last delivery? IF YOU DO NOT KNOW OR DO NOT REMEMBER, ENTER 01/01/1900.] |
| 282 | p182 | (CONTESTAN SI P0=3 o 4) ¿Cuál es la fecha de su última pérdida o aborto? SI NO SABE O NO RECUERDA, COLOCAR 01/01/1900. [(IF P0=3 or 4) What is the date of your last miscarriage or abortion? IF YOU DO NOT KNOW OR DO NOT REMEMBER, ENTER 01/01/1900.] |
| 283 | p183 | (CONTESTAN SI P0=3 o 4) ¿Recibió atención médica durante o inmediatamente después del aborto? [(IF P0=3 or 4) Did you receive medical attention during or immediately after the abortion?] |
| 284 | p184 | (CONTESTAN SI P183 = 1) ¿Dónde recibió la atención médica? [(IF P183 = 1) Where did you receive medical attention?] |
| 285 | p185 | (CONTESTAN SI P0=1 o 2) ¿Dónde fue su último parto? [(IF P0=1 or 2) Where did your last delivery take place?] |
| 286 | p186 | (CONTESTAN SI P0=1 o 2) ¿El parto fue por cesárea? [(IF P0=1 or 2) Was the delivery by cesarean section?] |
| 287 | p187 | (CONTESTAN SI P186 = 1) ¿Esta cesárea fue por…? [(IF P186 = 1) Was this cesarean section due to…?] |
| 288 | p188 | (CONTESTAN SI P0=1 o 2) Una vez dada de alta del último parto, ¿tuvo Ud. algún chequeo o control médico dentro de las 2 semanas posteriores? [(IF P0=1 or 2) After being discharged from your last delivery, did you have any medical check-up within the following 2 weeks?] |
| 289 | p189 | (CONTESTAN SI P188 = 2) ¿Cuál fue la principal razón por qué no tuvo chequeo o revisión médica? [(IF P188 = 2) What was the main reason you did not have a medical check-up?] |
| 290 | p190 | (CONTESTAN SI P180=1 o 2) ¿Algún médico(a) o doctor(a) le diagnosticó depresión postparto? [(IF P180=1 or 2) Did any doctor diagnose you with postpartum depression?] |
| 291 | p191 | (CONTESTAN SI P190 = 1) ¿Tuvo tratamiento para esta depresión postparto? [(IF P190 = 1) Did you receive treatment for this postpartum depression?] |
| 292 | p192 | (CONTESTAN SI P0=1 o 2) ¿Mirando hacia atrás, cree usted que tuvo depresión postparto? [(IF P0=1 or 2) Looking back, do you think you had postpartum depression?] |
| 293 | p193 | (CONTESTAN SI P0=1 o 2) Después de este último parto, ¿usted amamantó a su hijo/a? [(IF P0=1 or 2) After this last delivery, did you breastfeed your child?] |
| 294 | p194 | [SOLO SI P180 = 1 o 2 y P193 = 1] ¿Por cuántos meses amamantó a su hijo/a? [[ONLY IF P180 = 1 or 2 and P193 = 1] For how many months did you breastfeed your child?] |
| 295 | p195 | [SOLO SI P180 = 1 o 2 y P193 = 1] ¿Cree usted que el amamantar afectó a su vida sexual…? LEER ALTERNATIVAS [[ONLY IF P180 = 1 or 2 and P193 = 1] Do you think breastfeeding affected your sex life...? READ OPTIONS] |
| 296 | p196 | (CONTESTAN SI P180 = 1 o 2) En su último parto, ¿a usted…? [(IF P180 = 1 or 2) During your last delivery, were you...?] |
| 297 | p197 | ¿Ha tenido menstruación o regla en el último año? [Have you had your period or menstruation in the past year?] |
| 298 | p198 | ¿Cuál es la razón por la cual usted no ha tenido menstruación o regla en el último año? [What is the reason you have not had your period or menstruation in the past year?] |
| 299 | p199 | (CONTESTAN SI P198 = 4 O 7) ¿Podría indicarme aproximadamente qué edad tenía usted en su última menstruación o regla? [(IF P198 = 4 or 7) Could you tell me approximately how old you were when you had your last period?] |
| 300 | p200 | (CONTESTAN SI P198 = 4 o 7) ¿Alguna vez ha consultado a un médico/a, ginecólogo/a o matrón/a por razones relacionadas con su menopausia? [(IF P198 = 4 or 7) Have you ever consulted a doctor, gynecologist, or midwife for reasons related to your menopause?] |
| 301 | p201 | (CONTESTAN SI P198 = 4 O 7) ¿Ha tomado hormonas (terapia hormonal) para la menopausia? [(IF P198 = 4 or 7) Have you taken hormones (hormone therapy) for menopause?] |
| 302 | p202 | ¿Alguna vez en su vida, un doctor o médico le ha dicho que tiene o que padece de...? [Has a doctor ever told you that you have or suffer from...?] |
| 303 | p203 | ¿A qué edad se lo diagnosticaron por última vez? [At what age were you last diagnosed with this condition?] |
| 304 | p204 | La última vez que usted tuvo esta enfermedad ¿Siguió tratamiento? [The last time you had this illness, did you follow a treatment?] |
| 305 | p205 | ¿Alguna vez ha sido operado por esto? [Have you ever had surgery for this?] |
| 306 | p206 | ¿Cómo descubrió que tenía alguna de esas infecciones? Si ha tenido más de una vez una infección, piense en la última. [How did you find out that you had any of those infections? If you had more than one, think of the last one.] |
| 307 | p207 | La última vez que fue diagnosticado de alguna infección de transmisión sexual, ¿se lo informó a su o sus parejas sexuales? [The last time you were diagnosed with a sexually transmitted infection, did you inform your sexual partner(s)?] |
| 308 | p208 | [RESPONDEN P202.10 = 2] Por cualquier razón, ¿Se ha hecho el examen del VIH o Sida en los últimos 12 meses? [[IF P202.10 = 2] For any reason, have you had an HIV or AIDS test in the last 12 months?] |
| 309 | p209 | [RESPONDEN P202.10 = 2 & P208 = 1] ¿Conoce su resultado? [[IF P202.10 = 2 & P208 = 1] Do you know your result?] |
| 310 | p210 | [SOLO PARA P202.10 = 1 & P208 = 1] ¿Por qué razón se hizo el examen del VIH o Sida? [[ONLY IF P202.10 = 1 & P208 = 1] What was the reason you had an HIV or AIDS test?] |
| 311 | p211 | [SOLO PARA P202.10 = 2 & P208 = 2] ¿Por qué razón no se ha hecho el examen del VIH o Sida en los últimos 12 meses? MARCAR TODAS LAS QUE CORRESPONDA [[ONLY IF P202.10 = 2 & P208 = 2] Why haven’t you had an HIV or AIDS test in the past 12 months? MARK ALL THAT APPLY] |
| 312 | p212 | Ahora le voy a hacer unas preguntas sobre VIH o Sida. Cree usted qué… LEA AL ENCUESTADO CADA PREGUNTA Y MARQUE SÍ O NO [Now I’m going to ask you some questions about HIV or AIDS. Do you believe that… READ EACH QUESTION TO THE RESPONDENT AND MARK YES OR NO] |
| 313 | p213 | ¿Conoce usted la medida “profilaxis de preexposición” o PREP como alternativa de prevención del VIH/Sida? [Do you know about “pre-exposure prophylaxis” or PrEP as an HIV/AIDS prevention measure?] |
| 314 | p214 | SI ES MUJER: Alguna vez en su vida, ¿Usted ha usado anticoncepción de emergencia, llamada píldora del día después o PAE? Por favor, considere solo el uso de la píldora del día después, y no otras píldoras que buscan tener el mismo efecto. [IF FEMALE: Have you ever used emergency contraception, known as the morning-after pill or ECP? Please consider only the use of the morning-after pill, and not other pills that seek the same effect.] |
| 315 | p215 | SI ES HOMBRE: Alguna vez en su vida, ¿En una relación sexual con una mujer, ella ha usado anticoncepción de emergencia llamada píldora del día después o PAE? Por favor, considere solo el uso de la píldora del día después, y no otras píldoras que buscan tener el mismo efecto. [IF MALE: Have you ever been in a sexual relationship with a woman who used emergency contraception, known as the morning-after pill or ECP? Please consider only the use of the morning-after pill, and not other pills that seek the same effect.] |
| 316 | p216 | (CONTESTA SI EN P124 o 215 = 1) En los últimos 12 meses, ¿Cuántas veces ha usado anticoncepción de emergencia? (INDICAR NUMERO DE VECES) Por favor, considere solo el uso de la píldora del día después, y no otras píldoras que buscan tener el mismo efecto. [(IF YES TO P124 OR 215 = 1) In the past 12 months, how many times have you used emergency contraception? (INDICATE NUMBER OF TIMES) Please consider only the morning-after pill.] |
| 317 | p217 | ¿Alguna vez en la vida, alguna de sus parejas ha interrumpido algún embarazo producto de una relación sexual con usted? [Has any of your partners ever terminated a pregnancy that resulted from sexual intercourse with you?] |
| 318 | p218 | ¿Esta decisión fue consensuada con usted? [Was this decision made jointly with your partner?] |
| 319 | p219 | ¿Alguna vez en la vida, usted intentó o realizó alguna acción para interrumpir un embarazo propio, pero sin concretarlo? [Have you ever attempted or taken any action to terminate your own pregnancy, but without completing it?] |
| 320 | p220 | ¿Alguna vez en la vida, usted interrumpió un embarazo propio, concretándolo? [Have you ever successfully terminated your own pregnancy?] |
| 321 | p221 | ¿Ese embarazo lo interrumpió bajo alguna de las causas indicadas en la Ley de Interrupción voluntaria del embarazo? [Was the termination carried out under any of the grounds established in the Law on Voluntary Termination of Pregnancy?] |
| 322 | p222 | ¿Por cuál causal lo interrumpió? [What was the reason or ground for terminating it?] |
| 323 | p223 | ¿Qué edad tenía usted cuando interrumpió ese embarazo? (INDICAR EDAD N AÑOS CUMPLIDOS) [How old were you when you terminated that pregnancy? (INDICATE AGE IN COMPLETED YEARS)] |
| 324 | p224 | ¿Cuántos meses de gestación tenía al momento de interrumpir ese embarazo? (ANOTAR SEMANAS. SI NO RECUERDA SEMANAS; ANOTAR MESES) [How many months pregnant were you when you terminated that pregnancy? (RECORD IN WEEKS; IF YOU DON’T REMEMBER, RECORD IN MONTHS)] |
| 325 | p225 | Cuando usted interrumpió ese embarazo ¿Qué relación tenía usted con la persona con la que se embarazó? [What was your relationship with the person who got you pregnant at the time of the pregnancy?] |
| 326 | p226 | Para interrumpir el embarazo usted puede haber usado uno o más métodos para hacerlo, ¿cuál o cuáles usó? MARQUE TODAS LAS QUE CORRESPONDA [To terminate the pregnancy, you may have used one or more methods. Which one(s) did you use? CHECK ALL THAT APPLY] |
| 327 | p227 | ¿Usted se hizo una ecografía después del aborto? [Did you have an ultrasound after the abortion?] |
| 328 | p228 | ¿Usted tuvo alguna consulta con un profesional de la salud para interrumpir ese embarazo? [Did you consult a health professional before terminating that pregnancy?] |
| 329 | p229 | ¿Tuvo complicaciones que requirieron atención médica en un servicio de salud a raíz del aborto? [Did you experience complications that required medical care after the abortion?] |
| 330 | p230 | ¿Usted tuvo alguna consulta con un profesional de la salud para realizar el seguimiento de la interrupción de ese embarazo? [Did you have a follow-up consultation with a health professional after the abortion?] |
| 331 | p231 | ¿Con quién? [With whom?] |
| 332 | p232 | ¿Se contactó con un colectivo u ONG que colabora con las mujeres en la realización del aborto? [Did you contact any organization or NGO that supports women in accessing abortion services?] |
| 333 | p233 | ¿Su pareja actual ha tenido alguna enfermedad o discapacidad que lo haya afectado a usted en su actividad sexual o el disfrute de la misma? [Has your current partner had any illness or disability in the past 12 months that has affected your sexual activity or enjoyment?] |
| 334 | p234 | En los últimos 12 meses, usted ha tenido alguna enfermedad o discapacidad que lo haya afectado a usted en su actividad sexual o el disfrute de esta? [In the past 12 months, have you had any illness or disability that affected your sexual activity or enjoyment?] |
| 335 | p235 | ¿Usted ha tomado algún medicamento que haya afectado negativamente su actividad sexual o el disfrute de esta? [Have you taken any medication in the past 12 months that negatively affected your sexual activity or enjoyment?] |
| 336 | p236 | ¿Con qué frecuencia usted ha experimentado alguna de las siguientes situaciones en su vida sexual? MARCAR TODAS LAS QUE CORRESPONDAN [How often have you experienced any of the following situations in your sex life? MARK ALL THAT APPLY] |
| 337 | p237 | ¿Esto que le pasa a usted, cree que es un problema para la vida sexual con su pareja? [Do you think this affects your sex life with your partner?] |
| 338 | p238 | ¿Usted ha consultado o pedido ayuda a algún profesional de la salud por las situaciones que mencionó anteriormente? [Have you sought help from a health professional for the situations you mentioned?] |
| 339 | p239 | ¿A qué profesional o profesionales? [Which professional(s)?] |
| 340 | p240 | ¿Alguna vez en su vida ha experimentado las siguientes situaciones en lugares públicos, sin su consentimiento, tales como calles, plazas, transporte público, centros comerciales, cines, estadios, conciertos, marchas u otros espacios similares a estos? [Have you ever experienced the following situations in public spaces without your consent, such as streets, plazas, public transport, shopping centers, cinemas, stadiums, concerts, marches, or other similar places?] |
| 341 | p241 | ¿Le ocurrió en el último año? [Did it happen in the past year?] |
| 342 | p242 | ¿A qué edad le ocurrió por primera vez o única vez esta situación? [At what age did this situation happen to you for the first or only time?] |
| 343 | p243 | ¿En cuál o cuáles de los siguientes lugares le ha ocurrido esa situación alguna vez en la vida? TODAS LAS QUE CORRESPONDAN [In which of the following places has this situation occurred in your life? SELECT ALL THAT APPLY] |
| 344 | p244 | ¿Alguna vez en la vida ha experimentado alguna de las siguientes situaciones? [Have you ever experienced any of the following situations in your life?] |
| 345 | p245 | ¿Qué tan frecuentemente le sucedió esto en los últimos 5 años? [How frequently did this happen to you in the last 5 years?] |
| 346 | p246 | Pensando la última vez que le sucedió ¿Dónde le ocurrió? [Thinking of the last time this happened, where did it occur?] |
| 347 | p247 | ¿Alguna vez en la vida alguien te tocó tus partes privadas, tus genitales o tus pechos, bajo manipulación, engaño, sometimiento u obligación, y/o te forzaron a hacerlo? [Has anyone ever touched your private parts, genitals or breasts under manipulation, deceit, coercion or obligation, and/or forced you to do it?] |
| 348 | p248 | ¿Y en esa oportunidad te penetraron oral, anal o vaginalmente, con el pene, los dedos u otro objeto, bajo manipulación, engaño, sometimiento u obligación, y/o te forzaron a hacerlo? [And at that time, were you penetrated orally, anally or vaginally, with a penis, fingers or another object under manipulation, deceit, coercion or obligation, and/or were you forced to do it?] |
| 349 | p249 | ¿Y esto, cuántas veces le ocurrió? [How many times has this happened to you?] |
| 350 | p250 | ¿Qué edad tenía usted cuando ocurrió esto? [How old were you when this happened?] |
| 351 | p251 | ¿Qué edad tenía usted la primera vez? [How old were you the first time?] |
| 352 | p252 | ¿Qué edad tenía usted la última vez? [How old were you the last time?] |
| 353 | p253 | Pensando en la primera o única vez ¿Quién le hizo esto? [Thinking of the first or only time, who did this to you?] |
| 354 | p254 | (CONTESTA SI P249 = 1,2) Y pensando en la primera o única vez ¿La persona que le hizo esto era hombre o mujer? [If P249 = 1 or 2: Thinking about the first or only time, was the person who did this to you a man or a woman?] |
| 355 | p255 | (CONTESTA SI P249 = 3,4) Y pensando en la primera o única vez ¿Las personas que le hicieron esto eran hombres o mujeres? [If P249 = 3 or 4: Thinking about the first or only time, were the people who did this to you men or women?] |
| 356 | p256 | (CONTESTA SI P249 = 1, 2) Y pensando en la primera o única vez ¿Qué edad aproximadamente tenía la persona que le hizo esto? [If P249 = 1 or 2: Thinking about the first or only time, approximately how old was the person who did this to you?] |
| 357 | p257 | (CONTESTA SI P249 = 3, 4) Y pensando en la primera o única vez ¿Qué edad en promedio tenían las personas que le hicieron esto? [If P249 = 3 or 4: Thinking about the first or only time, what was the average age of the people who did this to you?] |
| 358 | p258 | ¿Ha hablado de esto con alguien? [Have you talked to anyone about this?] |
| 359 | p259 | (RESPONDEN P258 = 1) ¿Y con quién habló de ello? [If answered yes to P258: Who did you talk to about it?] |
| 360 | p260 | ¿Usted realizó una denuncia a la Justicia por este hecho? [Did you report this incident to the justice system?] |
| 361 | p261 | (CONTESTAN SI P260 = 2) ¿Cuál fue la principal razón por la qué no realizó una denuncia a la Justicia? [If P260 = 2: What was the main reason why you did not report it to the justice system?] |
| 362 | p262 | ¿Siente Ud. que esta situación lo(la) afectó de manera significativa en su vida? [Do you feel that this situation significantly affected your life?] |
| 363 | p263 | ¿Cuál es su nacionalidad? [What is your nationality?] |
| 364 | p264 | ¿Cuál? [Which one?] |
| 365 | p265 | Cuándo usted nació, ¿En qué país vivía su madre? [When you were born, in which country was your mother living?] |
| 366 | p266 | ¿Cuál? [Which one?] |
| 367 | p267 | En Chile, la ley reconoce diez pueblos indígenas, ¿pertenece Usted o es descendiente de alguno de ellos? [In Chile, the law recognizes ten Indigenous peoples. Do you belong to or are you a descendant of any of them?] |
| 368 | p268 | ¿Cuál es su religión o credo? [What is your religion or belief?] |
| 369 | p269 | ¿Usted se definiría como una persona…? (LEA ALTERNATIVAS) [How would you define yourself as a person...? (READ ALTERNATIVES)] |
| 370 | p270 | En general, la gente suele situarse en posiciones políticas más cercanas a la izquierda, al centro o a la derecha. En una escala de 1 a 10, donde 1 es izquierda y 10 es derecha, ¿En qué lugar se ubicaría usted? Elija el número que quiera según se inclina más hacia uno u otro lado [In general, people tend to position themselves politically closer to the left, center, or right. On a scale from 1 to 10, where 1 is left and 10 is right, where would you place yourself? Choose the number that best reflects your tendency.] |
| 371 | p271 | ¿Cuántos dormitorios, de uso exclusivo para dormir, tiene su vivienda? (INDICAR EL NUMERO DE PIEZAS) [How many bedrooms, used exclusively for sleeping, does your household have? (INDICATE THE NUMBER OF ROOMS)] |
| 372 | p272 | La semana pasada, ¿usted trabajó al menos una hora, sin considerar los quehaceres del hogar? [Last week, did you work at least one hour, not counting household chores?] |
| 373 | p273 | Aunque no trabajó la semana pasada, ¿usted realizó alguna actividad por lo menos durante una hora? …por un salario o remuneración? ¿en su empresa o negocio? para la empresa o negocio de un familiar (con o sin remuneración)? por pago en especies? como aprendiz o realizando una práctica remunerada? de venta, ¿sin incluir bienes del hogar? agrícola, minera o artesanal para la venta? [Even if you didn’t work last week, did you do any activity for at least one hour… for a salary or payment? In your own business or company? For a family business (with or without payment)? In exchange for goods? As an apprentice or in a paid internship? For sales, excluding household items? In agricultural, mining, or craft work for sale?] |
| 374 | p274 | ¿Aunque no trabajó la semana pasada, %NOMBRE% tenía algún empleo, negocio u otra actividad del cual estuvo ausente temporalmente por licencia, permiso postnatal parental, huelga, enfermedad, vacaciones, suspensión temporal u otra razón? [Although %NAME% did not work last week, did they have any job, business, or other activity from which they were temporarily absent due to leave, parental leave, strike, illness, vacation, temporary suspension, or other reason?] |
| 375 | p275 | ¿%NOMBRE% buscó trabajo remunerado o realizó alguna gestión para iniciar una actividad por cuenta propia (negocio o empresa) en las últimas cuatro semanas? [Has %NAME% looked for paid work or taken any steps to start a self-employed activity (business or enterprise) in the last four weeks?] |
| 376 | p276 | (CONTESTAN P272=1 o P273=1 o P274=1) ¿Cuál es su ocupación u oficio? [What is your occupation or trade?] |
| 377 | p277 | (CONTESTAN P272=1 o P273=1 o P274=1) ¿Qué hace usted en su trabajo o negocio principal? [What do you do in your main job or business?] |
| 378 | p278 | (CONTESTAN P272=1 o P273=1 o P274=1) ¿A qué se dedica o qué hace el negocio, empresa o institución donde usted trabaja? [What does the business, company, or institution where you work do?] |
| 379 | p279 | (CONTESTAN P272=1 o P273=1 o P274=1) En su trabajo o negocio principal, ¿Usted trabaja como? [In your main job or business, do you work as...?] |
| 380 | p280 | En su trabajo principal, ¿tiene contrato de trabajo escrito? [In your main job, do you have a written work contract?] |
| 381 | p281 | ¿A qué sistema previsional de salud pertenece usted? [Which health insurance system are you affiliated with?] |
| 382 | p282 | [SOLO PARA PERSONAS CON PAREJA P81 = 1] ¿Cuál es el nivel educacional más alto alcanzado o nivel educacional actual de su pareja? [What is the highest level of education your partner has completed or is currently attending?] |
| 383 | p283 | [SOLO PARA PERSONAS CON PAREJA P81 = 1] En ese nivel educacional, ¿cuál fue el último curso que su pareja aprobó o que cursa actualmente? [In that educational level, what was the last grade your partner completed or is currently attending?] |
| 384 | p284 | ¿Cuál fue el ingreso total de su hogar en el último mes? [What was your household’s total income last month?] |
| 385 | p285 | ¿Podría decirme en cuál de estos tramos está el ingreso total mensual de su hogar? [Could you tell me in which of these ranges your household’s monthly income falls?] |
| 386 | p286 | ¿Cree usted poder entregar información respecto a educación, ocupación e ingresos de los miembros del hogar? [Do you think you can provide information about the education, occupation, and income of household members?] |
| 387 | p287 | ¿Quién entregará información respecto de los miembros del hogar? [Who will provide information about the household members?] |
| 388 | p288 | ¿Cuál es el nivel educacional más alto alcanzado o nivel educacional actual de %MIEMBRO%? [What is the highest level of education %MEMBER% has completed or is currently attending?] |
| 389 | p289 | ¿Cuál fue el último curso que aprobó o que cursa actualmente %MIEMBRO%? [What was the last grade %MEMBER% completed or is currently attending?] |
| 390 | p290 | ¿%MIEMBRO% trabajó al menos una hora la semana pasada, sin contar quehaceres del hogar? [Did %MEMBER% work at least one hour last week, not counting household chores?] |
| 391 | p291 | ¿%MIEMBRO% realizó alguna actividad por lo menos durante una hora la semana pasada? [Did %MEMBER% perform any activity for at least one hour last week?] |
| 392 | p292 | ¿%MIEMBRO% tenía algún empleo, negocio u otra actividad del cual estuvo ausente temporalmente? [Did %MEMBER% have any job, business, or other activity from which they were temporarily absent?] |
| 393 | p293 | ¿%MIEMBRO% buscó trabajo remunerado o hizo alguna gestión para iniciar una actividad por cuenta propia en las últimas cuatro semanas? [Did %MEMBER% seek paid work or take any steps to start a self-employed activity in the past four weeks?] |
| 394 | p294 | ¿Cuál es la ocupación u oficio de %MIEMBRO%? (Indique el nombre completo del empleo u ocupación principal, y facilite detalles, por ejemplo: recolector de frutas, profesor de escuela nivel secundario, enfermera titulada, conductor de bus, guardia de seguridad, gerente de una empresa, entre otros). [What is %MIEMBRO%'s occupation or job? (Please specify the full name of the main job or occupation and provide details, for example: fruit picker, secondary school teacher, registered nurse, bus driver, security guard, company manager, among others).] |
| 395 | p295 | ¿Qué hace %MIEMBRO% en su trabajo o negocio principal? (Facilite detalles, por ejemplo: recoger y transportar uvas, enseñar matemáticas, cuidar enfermos y administrar medicamentos, transportar pasajeros entre ciudades, vigilar y controlar entrada en una empresa, administrar una empresa de productos lácteos). [What does %MIEMBRO% do in their main job or business? (Provide details, e.g., harvest and transport grapes, teach mathematics, care for the sick and administer medications, transport passengers between cities, monitor and control entry to a company, manage a dairy product business).] |
| 396 | p296 | ¿A qué se dedica o qué hace el negocio, empresa o institución donde %MIEMBRO% trabaja? (Describa la actividad a que se dedica la empresa, negocio o institución en que la persona realiza su actividad u ocupación principal). [What does the business, company or institution where %MIEMBRO% works do? (Describe the main activity of the business or institution where the person performs their main job).] |
| 397 | p297 | En su trabajo o negocio principal, ¿%MIEMBRO% trabaja como...? [In their main job or business, does %MIEMBRO% work as...?] |
| 398 | p298 | En %MES PASADO%, ¿cuál fue el ingreso de %MIEMBRO% proveniente de su o sus trabajos, ocupación o actividad? [In %LAST MONTH%, what was %MIEMBRO%'s income from their job(s), occupation, or activity?] |
| 399 | p299 | En %MES%, ¿cuál fue el ingreso que recibió %MIEMBRO% por jubilación o pensión? [In %MONTH%, what income did %MIEMBRO% receive from retirement or pension?] |
| 400 | p300 | En %MES PASADO%, ¿cuál fue el ingreso total que recibió %MIEMBRO% por subsidios, bonos o aportes del Estado? [In %LAST MONTH%, what was the total income %MIEMBRO% received from state subsidies, bonuses or contributions?] |
| 401 | p301 | Los últimos 12 meses, ¿cuál fue el ingreso que recibió %MIEMBRO% por los siguientes subsidios, bonos o aportes del Estado? [In the last 12 months, what income did %MIEMBRO% receive from the following subsidies, bonuses or state contributions?] |
| 402 | p302 | En %MES PASADO%, ¿cuál fue el ingreso total que recibió %MIEMBRO% por arriendos urbanos, pensión de alimentos o dinero aportado por terceros ajenos al hogar? [In %LAST MONTH%, what was the total income %MIEMBRO% received from urban rentals, alimony or money contributed by third parties outside the household?] |
| 403 | p303 | ¿Cuál fue el ingreso total de su hogar en el último mes? [What was the total income of your household last month?] |
| 404 | p304 | ¿Podría decirme en cuál de estos tramos está el ingreso total mensual de su hogar? [Could you tell me which of these ranges your household's total monthly income falls into?] |
| 405 | p305 | ¿Diría que el ingreso mensual del hogar les permite pagar sin necesidad de créditos o préstamos? [Would you say your household's monthly income allows you to pay without needing loans or credit?] |

Appendix 8 – Supplementary Table X. Full Classification of Survey Items by COM-B, TDF Domains, Response Thresholds, and Behavioral Outcomes

| Supplementary Table X. Full Classification of Survey Items by COM-B, TDF Domains, Response Thresholds, and Behavioral Outcomes | | | | | | |
| --- | --- | --- | --- | --- | --- | --- |
| **N°** | **VARIABLE** | **Summary Label** | **Agreed TDF Domain** | **COM Sub-Constructs** | **COM-B** | **Final Classification** |
| 1 | i_1_p33 | Condoms reduce sexual pleasure in women | Beliefs about consequences | Reflective motivation | Motivation | Enabler |
| 2 | i_2_p33 | Condoms reduce sexual pleasure in men | Beliefs about consequences | Reflective motivation | Motivation | Moderate Barrier |
| 3 | i_3_p33 | condom use necessary even in stable relationships | Knowledge | Psychological Capability | Capability | Moderate Barrier |
| 4 | i_4_p33 | condom use enhances sexual play | Beliefs about consequences | Reflective motivation | Motivation | Major Barrier |
| 5 | i_5_p33 | condoms are too expensive | Environmental context and resources | Physical opportunity | Opportunity | Enabler |
| 6 | p34 | Family discussed sexual topics in childhood | Social influences | Social opportunity | Opportunity | Major Barrier |
| 7 | p35 | Participation in sexual topic conversations | Social influences | Social opportunity | Opportunity | Enabler |
| 8 | t_p36_1 | Sex education in primary school | Environmental context and resources | Physical opportunity | Opportunity | Major Barrier |
| 9 | t_p36_2 | Sex education in secondary school | Environmental context and resources | Physical opportunity | Opportunity | Moderate Barrier |
| 10 | p37 | Perceived adequacy of sexual education | Beliefs about capacity | Reflective motivation | Motivation | Major Barrier |
| 11 | p38 | Perceived quality of sex education | Beliefs about capacity | Reflective motivation | Motivation | Major Barrier |
| 12 | i_2_p39 | Knowledge of STI prevention | Knowledge | Psychological Capability | Capability | Moderate Barrier |
| 13 | i_1_p40_o1 | Informational sources about sex | Environmental context and resources | Physical opportunity | Motivation | Descriptive use only |
| 14 | i_3_p40_o1 | Informational sources about STI prevention | Environmental context and resources | Physical opportunity | Motivation | Descriptive use only |
| 15 | p55 | Communication before first sex | Social influences | Social opportunity | Motivation | Major Barrier |
| 16 | p56 | Contraceptive use at first sex | Behavioural regulation | Psychological Capability | Capability | Major Barrier |
| 17 | p57 | Contraceptive use at first sex | Behavioural regulation | Psychological Capability | Capability | Descriptive use only |
| 18 | p58 | Main reason for condom use | Goals | Reflective motivation | Motivation | Descriptive use only |
| 19 | p59 | Source of contraceptive method | Environmental context and resources | Physical opportunity | Opportunity | Descriptive use only |
| 20 | p73 | Condom use frequency (last year) | Behavioural regulation | Psychological Capability | Capability | Major Barrier |
| 21 | p89 | Condom use after reconciliation | Behavioural regulation | Psychological Capability | Capability | Major Barrier |
| 22 | p103 | Substance use before sex | Reinforcement | Automatic motivation | Motivation | Descriptive use only |
| 23 | p104 | Partner’s substance use before sex | Social influences | Social opportunity | Opportunity | Descriptive use only |
| 24 | p119 | Contraceptive use in last sex | Behavioural regulation | Psychological Capability | Capability | Major Barrier |
| 25 | p120 | Contraceptive methods used | Behavioural regulation | Psychological Capability | Capability | Descriptive use only |
| 26 | p121 | Condom use motivation – STI/VIH prevention | Goals | Reflective motivation | Motivation | Enabler |
| 27 | p122 | Source of contraceptive method | Environmental context and resources | Physical opportunity | Opportunity | Descriptive use only |
| 28 | p123 | Reason for not using contraception | Beliefs about capabilities | Reflective motivation | Motivation | Descriptive use only |
| 29 | p151 | Ever sought sexual health consultation (women only) | Environmental context and resources | Physical opportunity | Opportunity | Moderate Barrier |
| 30 | p152 | Ever sought consultation for STI (men only) | Environmental context and resources | Physical opportunity | Opportunity | Major Barrier |
| 31 | p154 | Reason for first sexual health consultation | Goals | Reflective motivation | Motivation | Descriptive use only |
| 32 | p155 | Place of sexual health care | Environmental context and resources | Physical opportunity | Opportunity | Descriptive use only |
| 33 | p206 | Mode of STI diagnosis | Environmental context and resources | Physical opportunity | Opportunity | Descriptive use only |
| 34 | p207 | Disclosure of STI diagnosis to partner | Behavioural regulation | Psychological Capability | Capability | Enabler |
| 35 | p208 | HIV test in past 12 months | Behavioural regulation | Psychological Capability | Capability | Major Barrier |
| 36 | p210 | Reason for HIV testing | Beliefs about consequences | Reflective motivation | Motivation | Descriptive use only |
| 37 | p211 | Reasons for not taking HIV test (12m) | Beliefs about consequences | Reflective motivation | Motivation | Descriptive use only |
| 38 | i_1_p212 | Knowledge of condom efficacy for HIV | Knowledge | Psychological Capability | Capability | Enabler |
| 39 | i_2_p212 | Knowledge of condom efficacy for HIV | Knowledge | Psychological Capability | Capability | Enabler |
| 40 | i_3_p212 | Knowledge that HIV may be asymptomatic | Knowledge | Psychological Capability | Capability | Enabler |
| 41 | i_4_p212 | Rejects HIV transmission via mosquito bite | Knowledge | Psychological Capability | Capability | Enabler |
| 42 | i_5_p212 | Rejects HIV transmission via food sharing | Knowledge | Psychological Capability | Capability | Enabler |
| 43 | i_6_p212 | Aware of mother-to-child HIV transmission | Knowledge | Psychological Capability | Capability | Enabler |
| 44 | p213 | Awareness of PrEP for HIV prevention | Knowledge | Psychological Capability | Capability | Major Barrier |

Appendix 9 – Supplementary Table S2. Classification of Analyzed vs. Contextual Variables

This table summarizes the classification of key variables from the ENSSEX 2022–2023 survey used in the behavioral analysis, indicating whether each item was used as a behavioral determinant or for descriptive context only.

| **Variable (Survey Item)** | **Used as Behavioral Determinant** | **Framework Mapping** | **Classification** |
| --- | --- | --- | --- |
| p73 – Consistent condom use (last 12 months) | Yes | Behavioral regulation (COM-B, TDF) | Major barrier |
| p208 – HIV/STI testing (last 12 months) | Yes | Behavioral regulation (COM-B, TDF) | Major barrier |
| p34 – Family discussion of sexual topics | No | Social influences (context only) | Descriptive |
| p38 – Evaluation of school sex education | No | Beliefs about capabilities (context only) | Descriptive |
| p55 – Discussion of STI/pregnancy prevention at first sex | No | Social influences / Intentions (context only) | Descriptive |

# Appendix Supplementary Table S1

Detailed classifications and item-level data are presented in Table S1

**Table S1** Linking and classifying behavioral survey items within the COM-B model and TDF domains, and categorizing behavioral determinants as barriers or enablers

| **N°** | **VARIABLE** | **Questionnaire item (original in Spanish with English translation)** | **Summary Label** | **Agreed TDF Domain** | **Scale Used (1–5, 1–3, 1–7, Yes/No, Multiple)** | **Results Mean** | **SD** | **% in Enabler Response Category** | **Threshold Classification Criteria** | **Final Classification** |
| --- | --- | --- | --- | --- | --- | --- | --- | --- | --- | --- |
| 1 | i_1_p33 | Según lo que usted cree, ¿qué tan de acuerdo está con que usar preservativos o condón disminuye el placer de las mujeres? [How much do you believe that using condoms reduces women's sexual pleasure?] | Condoms reduce sexual pleasure in women | Beliefs about Consequences | Likert (1-5) | 2.79 | 1.038 | - | ≥4 Major Barrier; 3-3.9 Moderate Barrier; ≤ 2.99 Enabler | Enabler |
| 2 | i_2_p33 | Según lo que usted cree, ¿qué tan de acuerdo está con que usar preservativos o condón disminuye el placer de los hombres? [How much do you believe that using condoms reduces men's sexual pleasure?] | Condoms reduce sexual pleasure in men | Beliefs about Consequences | Likert (1-5) | 3.015 | 1.067 | - | ≥4 Major Barrier; 3-3.9 Moderate Barrier; ≤ 2.99 Enabler | Moderate Barrier |
| 3 | i_3_p33 | Según lo que usted cree, ¿es necesario ocupar preservativo o condón incluso si se tiene pareja estable? [How necessary do you think it is to use condoms even when in a stable relationship?] | condom use necessary even in stable relationships | Knowledge | Likert (1-5) | 3.087 | 1.071 | - | ≥4 Enabler; 3-3.9 Moderate Barrier; ≤ 2.99 Major Barrier | Moderate Barrier |
| 4 | i_4_p33 | Según lo que usted cree, ¿usar preservativo o condón estimula el juego sexual? [To what extent do you believe that using condoms enhances sexual enjoyment?] | condom use enhances sexual enjoyment | Beliefs about Consequences | Likert (1-5) | 2.834 | 0.987 | - | ≥4 Enabler; 3-3.9 Moderate Barrier; ≤ 2.99 Major Barrier | Major Barrier |
| 5 | i_5_p33 | Según lo que usted cree, ¿los preservativos o condones son demasiado caros para usarlos regularmente? [To what extent to you agree with the statement "Condoms too expensive to use regularly"?] | condoms are too expensive | Environmental Context and Resources | Likert (1-5) | 2.535 | 1.038 | - | ≥4 Major Barrier; 3-3.9 Moderate Barrier; ≤ 2.99 Enabler | Enabler |
| 6 | p34 | Cuando usted era niño/a, ¿En su familia se conversaban temas sexuales? [When you were a child, did your family talk about sexuality?] | Family discussed sexual topics in childhood | Social Influences | Ordinal (1–3); classified by % responses = 3 | 1.356 | 0.611 | 7.16% (value 3 only); or 28.81% (values 2 or 3) | 3 = Enabler; 2 = Moderate Barrier; 1 = Major Barrier. Classified based on % value 3 only (7.16%) → Major Barrier | Major Barrier |
| 7 | p35 | ¿Y con qué frecuencia usted participaba cuando se conversaban temas sexuales? [And how often did you talk when sexual topics were discussed?] | Participation in sexual topic conversations | Social Influences | Ordinal (1–3) | 1.733 | 0.630 | 89.9% (values 2 or 3) | 1 = Never; 2 = Sometimes; 3 = Always or almost always. % in values 2 or 3 = 89.9% → ≥70% = Enabler | Enabler |
| 8 | t_p36_1 | En su escuela, cuándo usted era estudiante, ¿se impartía educación sexual en Enseñanza básica? [When you were in elementary school, did you have sex education?] | Sex education in primary school | Environmental Context and Resources | Dichotomous (Yes/No) | - | - | 30.28% ("Yes") | % “Yes” = 30.28%. Classified as: <40% → Major Barrier | Major Barrier |
| 9 | t_p36_2 | En su escuela, cuándo usted era estudiante, ¿se impartía educación sexual en Enseñanza media? [When you were in high school, did you have sex education?] | Sex education in secondary school | Environmental Context and Resources | Dichotomous (Yes/No) | - | - | 52.09% ("Yes") | % “Yes” = 52.09%. Classified as: 40–69% → Moderate Barrier | Moderate Barrier |
| 10 | p37 | Y respecto de esa educación sexual, en general, usted cree que… [And did you consider the sex education you received at school...] | Perceived adequacy of sexual education | Beliefs about Capacity | Ordinal (1–3) | 2.429 | 0.621 | - | 1 = Less than needed; 2 = What I needed; 3 = More than needed. % in value 3 = 7.09% → Major Barrier | Major Barrier |
| 11 | p38 | ¿Cómo evaluaría en general la formación en sexualidad que recibió en su colegio o escuela? [How would you evaluate the sex education you received in school?] | Perceived quality of sex education | Beliefs about Capacity | Likert (1-5) | 2.518 | 1.149 | 23.04% (“Good” or “Very good”) | ≥4 Enabler; 3-3.9 Moderate Barrier; ≤ 2.99 Major Barrier | Major Barrier |
| 12 | i_2_p39 | ¿Qué nota le podría ahora al conocimiento que usted tenía en los siguientes temas (Métodos para prevenir infecciones de transmisión sexual), cuando era adolescente? [How would you now grade the knowledge you had on the following topics (methods to prevent sexually transmitted infections), when you were an adolescent?] | Knowledge of STI prevention | Knowledge | Likert (1-7) | 3.796 | 2.211 | 42.65% (values 5–7) | ≥60% Enabler; 40–59% Moderate Barrier; <40% Major Barrier | Moderate Barrier |
| 13 | i_1_p40_o1 | Cuando usted era adolescente, a raíz de alguna duda sobre los siguientes temas, ¿A quién recurrió para resolverlas? Relaciones sexuales [As an adolescent, if you had questions about sexual intercourse, who did you turn to for answers?] | Information sources about sex | Environmental Context and Resources | Nominal Categorical | - | - | - | Not applicable to this scale | Descriptive use only |
| 14 | i_3_p40_o1 | Cuando usted era adolescente, a raíz de alguna duda sobre los siguientes temas, ¿A quién recurrió para resolverlas? (Métodos preventivos de infecciones de transmisión sexual, como el VIH)  [As an adolescent, if you had questions about methods to prevent sexually transmitted infections like HIV, who did you turn to for answers?] | Information sources about STI prevention | Environmental Context and Resources | Nominal Categorical | - | - | - | Not applicable to this scale | Descriptive use only |
| 15 | p55 | Antes de su primera relación sexual, ¿Usted y esa persona hablaron de cómo evitar una ITS…?  [Before having intercourse for the first time, did you and your partner talk about how to avoid STIs...?] | Communication before first sex | Social Influences | Dichotomous (Yes/No) | 1.76 | 0.424 | 23.62% ("Yes") | % “Yes” = 23.62%. Classified as: <40% → Major Barrier | Major Barrier |
| 16 | p56 | En esa primera relación sexual, ¿Ustedes usaron algún método anticonceptivo? [Did you use any contraceptive method the first time you had intercourse?] | Contraceptive use at first sex | Behavioral Regulation | Dichotomous (Yes/No) | 1.67 | 0.471 | 33.41% ("Yes") | % “Yes” = 33.41%. Classified as: <40% → Major Barrier | Major Barrier |
| 17 | p57 | ¿Cuál o cuáles métodos anticonceptivos usaron en esa primera relación sexual? [Which contraceptive methods did you use that first time?] | Contraceptive use at first sex | Behavioral Regulation | Nominal Categorical | - | - | - | Not applicable to this scale | Descriptive use only |
| 18 | p58 | ¿Cuál fue el principal motivo por el cual usaron preservativo o condón? [What was the main reason for using a condom that first time?] | Main reason for condom use | Goals | Nominal Categorical | - | - | - | Not applicable to this scale | Descriptive use only |
| 19 | p59 | ¿Dónde obtuvo el método o los métodos mencionados anteriormente? [Where did you get the contraceptive method(s) mentioned above?] | Source of contraception | Environmental Context and Resources | Nominal Categorical | - | - | - | Not applicable to this scale | Descriptive use only |
| 20 | p73 | En las relaciones con esas parejas sexuales del último año, ¿con qué frecuencia usted usaba condón o preservativo? [In your sexual relationships over the past year, how often did you use condoms?] | Condom use frequency (last year) | Behavioral Regulation | Ordinal (1–3) | 2.43 | 0.76 | 17.51% ("Always") | % “Always” = 17.51%. Classified as: <70% → Major Barrier | Major Barrier |
| 21 | p89 | Y en la primera relación sexual que volvió a tener con esa persona después de la separación ¿usaron condón o preservativo? [In the first sexual encounter you had with that person after the separation, did you use a condom?] | Condom use after reconciliation | Behavioral Regulation | Dichotomous (Yes/No) | 1.64 | 0.48 | 35.95% ("Yes") | % “Yes” = 35.95%. Classified as: <70% → Major Barrier | Major Barrier |
| 22 | p103 | Justo antes o al momento de las relaciones sexuales que ha tenido en el último mes, ¿usted consumió alguna de las siguientes sustancias? [Just before or during your sexual encounters in the last month, did you use any of the following substances?] | Substance use before sex | Reinforcement | Nominal Categorical | - | - | - | Not applicable to this scale | Descriptive use only |
| 23 | p104 | Y alguna de las personas con la que usted estaba, ¿había consumido alguna de las siguientes sustancias? [And did any of the people you were with use any of the following substances?] | Partner’s substance use before sex | Social Influences | Nominal Categorical | - | - | - | Not applicable to this scale | Descriptive use only |
| 24 | p119 | En esa última relación sexual, ¿Ustedes usaron alguno método anticonceptivo? [In that last sexual encounter, did you use any contraceptive method?] | Contraceptive use at last sex | Behavioral Regulation | Dichotomous (Yes/No) | 1.63 | 0.48 | 36.88% ("Yes") | % “Yes” = 36.88%. Classified as: <70% → Major Barrier | Major Barrier |
| 25 | p120 | ¿Cuál o cuáles métodos anticonceptivos utilizaron? [Which contraceptive method(s) did you use?] | Contraceptive methods used | Behavioral Regulation | Nominal Categorical | - | - | - | Not applicable to this scale | Descriptive use only |
| 26 | p121 | ¿Por qué motivo usaron preservativo o condón? [What was the reason for using a condom?] | Condom use motivation – STI/VIH prevention | Goals | Dichotomous (Yes/No) | 1.20 | 0.40 | 79.93% ("Yes") | % “Yes” = 79.93%. Classified as: ≥70% → Enabler | Enabler |
| 27 | p122 | ¿Dónde obtuvo el método o los métodos mencionados anteriormente? [Where did you get the contraceptive method(s) mentioned above?] | Source of contraception | Environmental Context and Resources | Nominal Categorical | - | - | - | Not applicable to this scale | Descriptive use only |
| 28 | p123 | ¿Por qué razón no usó ningún método anticonceptivo? [Why did you not use any contraceptive method?] | Reason for not using contraception | Beliefs about Capabilities | Nominal Categorical | - | - | - | Not applicable to this scale | Descriptive use only |
| 29 | p151 | ¿Alguna vez en su vida ha ido a una consulta o donde algún profesional de la salud para tratar asuntos médicos PROPIOS relacionados con la ginecología, pubertad, sexualidad, métodos preventivos del embarazo o infecciones de transmisión sexual? [Have you ever seen a health professional for personal medical concerns related to gynecology, puberty, sexuality, pregnancy prevention or sexually transmitted infections?] | Ever sought sexual health consultation (women only) | Environmental Context and Resources | Dichotomous (Yes/No) | 1.31 | 0.463 | 68.71% ("Yes") | % “Yes” = 68.71%. Classified as: 40–69% → Moderate Barrie | Moderate Barrier |
| 30 | p152 | ¿Alguna vez en su vida ha ido a una consulta o donde algún profesional de la salud para tratar asuntos médicos PROPIOS relacionados con la urología, pubertad, sexualidad, métodos preventivos del embarazo o infecciones de transmisión sexual? [Have you ever seen a health professional for personal medical concerns related to urology, puberty, sexuality, pregnancy prevention or sexually transmitted infections?] | Ever sought consultation for STI (men only) | Environmental Context and Resources | Dichotomous (Yes/No) | 1.76 | 0.43 | 24.31% ("Yes") | % “Yes” = 24.31%. Classified as: <40% → Major Barrier | Major Barrier |
| 31 | p154 | ¿Por qué motivo fue esa primera vez? [What was the reason for that first visit?] | Reason for first sexual health consultation | Goals | Nominal Categorical | - | - | - | Not applicable to this scale | Descriptive use only |
| 32 | p155 | ¿A qué lugar fue? [Where did you go?] | Place of sexual health care | Environmental Context and Resources | Nominal Categorical | - | - | - | Not applicable to this scale | Descriptive use only |
| 33 | p206 | ¿Cómo descubrió que tenía alguna de esas infecciones? Si ha tenido más de una vez una infección, piense en la última. [How did you find out you had one of these infections? If you have had more than one, answer for the most recent.] | Mode of STI diagnosis | Environmental Context and Resources | Nominal Categorical | - | - | - | Not applicable to this scale | Descriptive use only |
| 34 | p207 | La última vez que fue diagnosticado de alguna infección de transmisión sexual, ¿se lo informó a su o sus parejas sexuales? [The last time you were diagnosed with a sexually transmitted infection, did you inform your sexual partner(s)?] | Disclosure of STI diagnosis to partner | Behavioral Regulation | Categorical (1–3) | 1.43 | 0.71 | 70.33% ("Yes") | % “Yes” = 70.33%. Classified as: ≥70% → Enabler | Enabler |
| 35 | p208 | Por cualquier razón, ¿Se ha hecho el examen del VIH o Sida en los últimos 12 meses? [Have you had an HIV test for any reason in the past 12 months?] | HIV test in past 12 months | Behavioral Regulation | Dichotomous (Yes/No) | 1.774 | 0.40 | 22.59% ("Yes") | % “Yes” = 22.59%. Classified as: <40% → Major Barrier | Major Barrier |
| 36 | p210 | ¿Por qué razón se hizo el examen del VIH o Sida? [Why did you have an HIV test?] | Reason for HIV testing | Beliefs about Consequences | Nominal Categorical | - | - | - | Not applicable to this scale | Descriptive use only |
| 37 | p211 | ¿Por qué razón no se ha hecho el examen del VIH o Sida en los últimos 12 meses? [Why have you not had an HIV test in the past 12 months?] | Reasons for not getting tested for HIV (12m) | Beliefs about Consequences | Nominal Categorical | - | - | - | Not applicable to this scale | Descriptive use only |
| 38 | i_1_p212 | ¿Puede reducirse el riesgo de transmisión del VIH manteniendo relaciones sexuales con una única pareja fiel y sin VIH o Sida? [Can the risk of HIV transmission be reduced by having sex with one monogamous partner who does not have HIV/AIDS?] | Knowledge of condom efficacy for HIV | Knowledge | Dichotomous (Yes/No) | 1.24 | 0.424 | 76.40% ("Yes") | % “Yes” = 76.40%. Classified as: ≥70% → Enabler | Enabler |
| 39 | i_2_p212 | ¿Puede reducirse el riesgo de transmisión del VIH usando preservativo o condón cada vez que se mantienen relaciones sexuales? [Can the risk of HIV transmission be reduced by using condoms every time you have sex?] | Knowledge of condom efficacy for HIV | Knowledge | Dichotomous (Yes/No) | 1.15 | 0.36 | 85.13% ("Yes") | % “Yes” = 85.13%. Classified as: ≥70% → Enabler | Enabler |
| 40 | i_3_p212 | ¿Puede una persona de aspecto saludable tener VIH?  [Can a healthy-looking person have HIV?] | Knowledge that HIV may be asymptomatic | Knowledge | Dichotomous (Yes/No) | 1.14 | 0.35 | 85.66% ("Yes") | % “Yes” = 85.66%. Classified as: ≥70% → Enabler | Enabler |
| 41 | i_4_p212 | ¿Se puede adquirir el VIH por picaduras de mosquito? [Can HIV be transmitted through mosquitos?] | Rejects HIV transmission via mosquito bite | Knowledge | Dichotomous (Yes/No) | 1.75 | 0.43 | 74.55% ("No") | % “No” = 74.55%. Classified as: ≥70% → Enabler | Enabler |
| 42 | i_5_p212 | ¿Se puede adquirir el VIH por compartir alimentos con una persona con VIH/Sida? [Can HIV be transmitted by sharing food with someone with HIV/AIDS?] | Rejects HIV transmission via food sharing | Knowledge | Dichotomous (Yes/No) | 1.82 | 0.38 | 82.33% ("No") | % “No” = 82.33%. Classified as: ≥70% → Enabler | Enabler |
| 43 | i_6_p212 | ¿Se puede transmitir el VIH de la madre al niño/a en el embarazo, parto o durante la lactancia? [Can HIV be transmitted from mother to child during pregnancy, childbirth, or breastfeeding?] | Aware of mother-to-child HIV transmission | Knowledge | Dichotomous (Yes/No) | 1.18 | 0.38 | 82.00% ("Yes") | % “Yes” = 82.00%. Classified as: ≥70% → Enabler | Enabler |
| 44 | p213 | ¿Conoce usted la medida “profilaxis de preexposición” o PREP como alternativa de prevención del VIH/Sida? [Do you know about “pre-exposure prophylaxis” or PrEP as an alternative for HIV/AIDS prevention?] | Awareness of PrEP for HIV prevention | Knowledge | Dichotomous (Yes/No) | 1.89 | 0.31 | 10.50% ("Yes") | % “Yes” = 10.50%. Classified as: <40% → Major Barrier | Major Barrier |

Mapping of 44 ENSSEX items to COM-B and TDF domains. Classification based on item scale, descriptive statistics, and predefined thresholds. See Methods and Supplementary Appendix 5 for details.

# Appendix Supplementary Table S2

**Table S2** Classification of survey items on condom use according to COM-B and TDF

| **Item** | **VARIABLE** | **Summary Label** | **Agreed TDF Domain** | **COM Sub-Constructs** | **COM-B** | **Final Classification** |
| --- | --- | --- | --- | --- | --- | --- |
| 1 | i_1_p33 | Condoms reduce sexual pleasure in women | Beliefs about Consequences | Reflective Motivation | Motivation | Enabler |
| 2 | i_2_p33 | Condoms reduce sexual pleasure in men | Beliefs about Consequences | Reflective Motivation | Motivation | Moderate Barrier |
| 3 | i_3_p33 | Condom use necessary even in stable relationships | Knowledge | Psychological Capability | Capability | Moderate Barrier |
| 4 | i_4_p33 | Condom use enhances sexual enjoyment | Beliefs about Consequences | Reflective Motivation | Motivation | Major Barrier |
| 5 | i_5_p33 | Condoms are too expensive | Environmental Context and Resources | Physical Opportunity | Opportunity | Enabler |
| 6 | p34 | Family discussed sexuality in childhood | Social Influences | Social Opportunity | Opportunity | Major Barrier |
| 7 | p35 | Participation in family conversations about sexuality | Social Influences | Social Opportunity | Opportunity | Enabler |
| 8 | t_p36_1 | Sex education in elementary school | Environmental Context and Resources | Physical Opportunity | Opportunity | Major Barrier |
| 9 | t_p36_2 | Sex education in high school | Environmental Context and Resources | Physical Opportunity | Opportunity | Moderate Barrier |
| 10 | p55 | Communication before first sex | Social Influences | Social Opportunity | Motivation | Major Barrier |
| 11 | p56 | Contraceptive use at first sex | Behavioral Regulation | Psychological Capability | Capability | Major Barrier |
| 12 | p57 | Contraceptive use at first sex | Behavioral Regulation | Psychological Capability | Capability | Descriptive use only |
| 13 | p58 | Main reason for condom use | Goals | Reflective Motivation | Motivation | Descriptive use only |
| 14 | p59 | Source of contraception | Environmental Context and Resources | Physical Opportunity | Opportunity | Descriptive use only |
| 15 | p73 | Condom use frequency (last year) | Behavioral Regulation | Psychological Capability | Capability | Major Barrier |
| 16 | p120 | Contraceptive methods used | Behavioral Regulation | Psychological Capability | Capability | Descriptive use only |
| 17 | p121 | Condom use motivation – STI/VIH prevention | Goals | Reflective Motivation | Motivation | Enabler |
| 18 | p122 | Source of contraception | Environmental Context and Resources | Physical Opportunity | Opportunity | Descriptive use only |
| 19 | p123 | Reason for not using contraception | Beliefs about Capabilities | Reflective Motivation | Motivation | Descriptive use only |
| 20 | p154 | Reason for first sexual health consultation | Goals | Reflective Motivation | Motivation | Descriptive use only |
| 21 | p155 | Place of sexual health care | Environmental Context and Resources | Physical Opportunity | Opportunity | Descriptive use only |
| 22 | p207 | Disclosure of STI diagnosis to partner | Behavioral Regulation | Psychological Capability | Capability | Enabler |

# Appendix Supplementary Table S3

**Table S3** Classification of HIV/STI testing-related survey items according to COM-B and TDF

| **Item** | **VARIABLE** | **Summary Label** | **Agreed TDF Domain** | **COM Sub-Constructs** | **COM-B** | **Final Classification** |
| --- | --- | --- | --- | --- | --- | --- |
| 1 | p37 | Perceived adequacy of sex education | Beliefs about Capacity | Reflective Motivation | Motivation | Major Barrier |
| 2 | p38 | Perceived quality of sex education | Beliefs about Capacity | Reflective Motivation | Motivation | Major Barrier |
| 3 | i_2_p39 | Knowledge of STI prevention | Knowledge | Psychological Capability | Capability | Moderate Barrier |
| 4 | i_1_p40_o1 | Information sources about sex | Environmental Context and Resources | Physical Opportunity | Motivation | Descriptive use only |
| 5 | i_3_p40_o1 | Information sources about STI prevention | Environmental Context and Resources | Physical Opportunity | Motivation | Descriptive use only |
| 6 | p89 | Condom use after reconciliation | Behavioral Regulation | Psychological Capability | Capability | Major Barrier |
| 7 | p103 | Substance use before sex | Reinforcement | Automatic Motivation | Motivation | Descriptive use only |
| 8 | p104 | Partner’s substance use before sex | Social Influences | Social Opportunity | Opportunity | Descriptive use only |
| 9 | p119 | Contraceptive use in last sex | Behavioral Regulation | Psychological Capability | Capability | Major Barrier |
| 10 | p151 | Ever sought sexual health consultation (women only) | Environmental Context and Resources | Physical Opportunity | Opportunity | Moderate Barrier |
| 11 | p152 | Ever sought consultation for STI (men only) | Environmental Context and Resources | Physical Opportunity | Opportunity | Major Barrier |
| 12 | p206 | Mode of STI diagnosis | Environmental Context and Resources | Physical Opportunity | Opportunity | Descriptive use only |
| 13 | p208 | HIV test in past 12 months | Behavioral Regulation | Psychological Capability | Capability | Major Barrier |
| 14 | p210 | Reason for HIV testing | Beliefs about Consequences | Reflective Motivation | Motivation | Descriptive use only |
| 15 | p211 | Reasons for not getting tested for HIV (12m) | Beliefs about Consequences | Reflective Motivation | Motivation | Descriptive use only |
| 16 | i_1_p212 | Knowledge of condom efficacy for HIV | Knowledge | Psychological Capability | Capability | Enabler |
| 17 | i_2_p212 | Knowledge of condom efficacy for HIV | Knowledge | Psychological Capability | Capability | Enabler |
| 18 | i_3_p212 | Knowledge that HIV may be asymptomatic | Knowledge | Psychological Capability | Capability | Enabler |
| 19 | i_4_p212 | Rejects HIV transmission via mosquitos | Knowledge | Psychological Capability | Capability | Enabler |
| 20 | i_5_p212 | Rejects HIV transmission via food sharing | Knowledge | Psychological Capability | Capability | Enabler |
| 21 | i_6_p212 | Aware of mother-to-child HIV transmission | Knowledge | Psychological Capability | Capability | Enabler |
| 22 | p213 | Awareness of PrEP for HIV prevention | Knowledge | Psychological Capability | Capability | Major Barrier |
